# It’s Getting Complicated—A Fresh Look at p53-MDM2-ARF Triangle in Tumorigenesis and Cancer Therapy

**DOI:** 10.3389/fcell.2022.818744

**Published:** 2022-01-26

**Authors:** Che-Pei Kung, Jason D. Weber

**Affiliations:** ^1^ ICCE Institute, St. Louis, MO, United States; ^2^ Division of Molecular Oncology, Department of Medicine, St. Louis, MO, United States; ^3^ Alvin J. Siteman Cancer Center, Washington University School of Medicine, St. Louis, MO, United States

**Keywords:** p53, MDM2, p14ARF, ARF, *CDKN2A*, tumor suppressor, oncogene, cancer therapy

## Abstract

Anti-tumorigenic mechanisms mediated by the tumor suppressor p53, upon oncogenic stresses, are our bodies’ greatest weapons to battle against cancer onset and development. Consequently, factors that possess significant p53-regulating activities have been subjects of serious interest from the cancer research community. Among them, MDM2 and ARF are considered the most influential p53 regulators due to their abilities to inhibit and activate p53 functions, respectively. MDM2 inhibits p53 by promoting ubiquitination and proteasome-mediated degradation of p53, while ARF activates p53 by physically interacting with MDM2 to block its access to p53. This conventional understanding of p53-MDM2-ARF functional triangle have guided the direction of p53 research, as well as the development of p53-based therapeutic strategies for the last 30 years. Our increasing knowledge of this triangle during this time, especially through identification of p53-independent functions of MDM2 and ARF, have uncovered many under-appreciated molecular mechanisms connecting these three proteins. Through recognizing both antagonizing and synergizing relationships among them, our consideration for harnessing these relationships to develop effective cancer therapies needs an update accordingly. In this review, we will re-visit the conventional wisdom regarding p53-MDM2-ARF tumor-regulating mechanisms, highlight impactful studies contributing to the modern look of their relationships, and summarize ongoing efforts to target this pathway for effective cancer treatments. A refreshed appreciation of p53-MDM2-ARF network can bring innovative approaches to develop new generations of genetically-informed and clinically-effective cancer therapies.

## Introduction

Discovered more than 40 years ago, tumor-suppressor p53 (encoded by *TP53* in human and *Trp53* in mouse) has become the most popular gene due to the fact that it is the most frequently altered gene in cancers (Vogelstein et al., 2010; Dolgin, 2017). Functioning as guardian of the genome, p53 responds to oncogenic stresses by inducing mechanisms like cell cycle arrest, senescence and programmed cell death (apoptosis) to allow damaged cells to either undergo necessary repairs or be eradicated from the environment before permanent transformation leading to malignant cancer progression (Kastenhuber and Lowe, 2017).

Decades of extensive studies have revealed tremendous complexity of the p53 universe. A master regulator of systemic homeostasis, p53 regulates pathogenesis of many diseases other than cancer, including neurodegeneration, cardiovascular diseases, metabolic disorders, autoimmune and infectious diseases (Takatori et al., 2014; Siegl and Rudel, 2015; Kung and Murphy, 2016; Szybinska and Lesniak, 2017; Aloni-Grinstein et al., 2018; Maor-Nof et al., 2021; Men et al., 2021). As if we need a further reminder about p53’s significance in human health, the culprit of the global COVID-19 pandemic, SARS-CoV-2, also targets p53 for its full pathogenic effects (Cardozo and Hainaut, 2021). Connections between p53 and these diverse physiological conditions led to expanded knowledge of many biological processes downstream of p53, such as metabolism, autophagy, translational control and epigenetic regulation, among others (Levine, 2019; Boutelle and Attardi, 2021).

Equally complicated is the network of mechanisms regulating p53 functions. Regulation of p53 is dictated by many factors, including mutation status and post-translational modification of p53, composition of response elements (REs) of p53 target genes, interaction between p53 and cofactors, and the heterogeneity in spatial and temporal dynamics of p53 activity (Hafner et al., 2019; Farkas et al., 2021). It is a highly choreographed process to control cell fate through the huge number (>3,500 by estimation) of p53 target genes and other p53-controlled mechanisms (Fischer, 2017; Sammons et al., 2020).

Amidst the tremendous complexity surrounding p53, one constant is the central hub formed by p53 and its key regulator, mouse double minute 2 (MDM2). The relationship between p53 and MDM2 is considered the final gatekeeper for majority of stress-induced signaling pathways whose main objective is to unlock the power of p53-mediated activities (Levine, 2020). The importance of p53-MDM2 hub also signifies the critical roles of direct MDM2 regulators, chief among them alternate open reading frame (ARF), in controlling p53 functions. We will herein summarize the conventional understanding of p53-MDM2-ARF relationships, unconventional and unique perspectives provided by recent studies, and implications for cancer therapeutics as our knowledge of this powerful triangle continues to evolve.

## The Simple Triangle Connecting p53, MDM2 and ARF

### Conventional Wisdom for p53-MDM2-ARF Relationship

**FIGURE 1:**
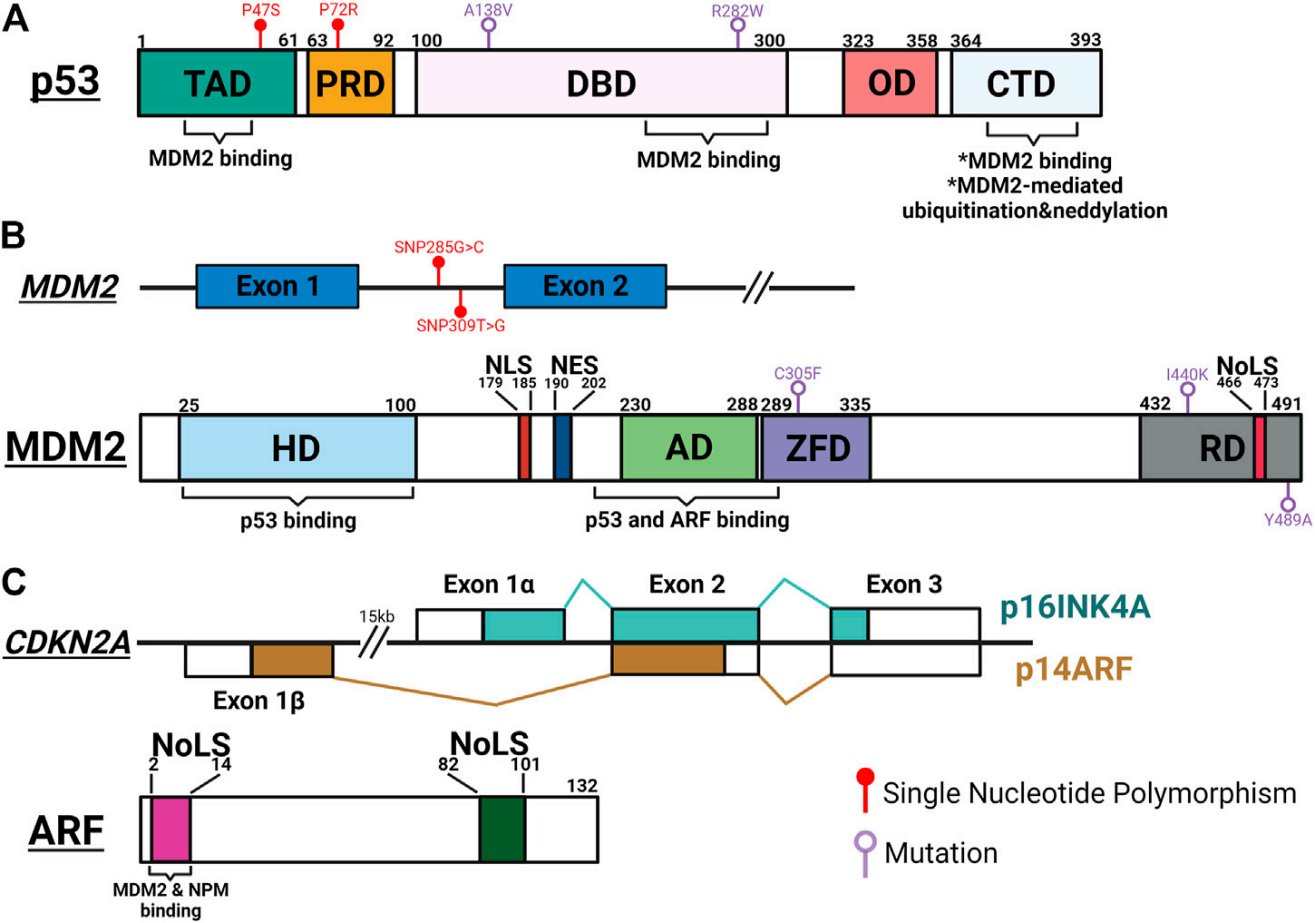
Schematic summary of important genetic and protein features of p53, MDM2 and ARF. **(A)** p53 interacts with MDM2 through its transactivation domain (TAD), DNA binding domain (DBD) and carboxy-terminal domain (CTD). MDM2-mediated post-translational modifications occur at the CTD, leading to inactivation and degradation of p53. Single nucleotide polymorphisms (SNP) in TAD (P47S; rs1800371) and proline-rich domain (PRD) (P72R; rs1042522) modulate p53’s ability to suppress tumorigenesis and regulate metabolic fitness. Temperature sensitive mutations of p53 in DBD (A138V and R282W) result in resistance to MDM2-mediated degradation. OD, oligomerization domain. **(B)** Upper panel: SNPs of *MDM2* regulate the functional oscillation between p53 and MDM2. SNP309T>G (rs2279744) results in higher MDM2 expression and inhibits p53-MDM2 oscillation. SNP285G>C (rs117039649) contributes to lower MDM2 expression and is associated with reduced risks for female reproductive cancers. Lower panel: MDM2 interacts with p53 through its N-terminal hydrophobic domain (HD) and acid domain (AD), and with ARF through AD. Interaction with ARF exposes the NoLS motif in the RING domain (RD) to sequester the ARF-MDM2 complex in the nucleolus. A cancer-associated single mutation, C305F, in the zinc finger domain (ZFD) mediates interaction between MDM2 and ribosomal proteins (RP) to regulate p53 function in response to metabolic stress. Two mutations in RD (I440K and Y489A) reduce MDM2-mediated p53 degradation but still limit p53 activity in response to DNA damage. **(C)** Upper panel: The *p16INK4A*/*p14ARF* locus. Each transcript utilizing a unique first exon, *p16INK4A* (Exon 1α) and *p14ARF* (Exon 1β) splice into common exon 2 and 3 in alternate reading frames to produce two distinctive amino acid sequences, resulting in two unrelated proteins. Lower panel: ARF interacts with MDM2 and NPM through its conserved N-terminal motif between amino acids 1 and 14. Both 1–14 and 82–101 arginine rich NoLS motifs are important for ARF’s ability to translocate to the nucleolus and activate p53. The figure was created with BioRender.com and not drawn to scale.

To deploy anti-tumorigenic functions, wild type (WT) p53 stands ready to be activated in short orders, while maintaining in the background of cellular machineries to prevent unnecessary damages. This fast-deployment system requires a simple mechanism for on and off switches, controlled mainly by a single protein, MDM2. Initially recognized as an oncogene overexpressed in transformed mouse cells, MDM2 was quickly found to promote tumorigenesis by inhibiting p53’s transcriptional activity (Fakharzadeh et al., 1991; Cahilly-Snyder et al., 1987; Oliner et al., 1993). The structure of MDM2 contains a main N-terminal p53-binding domain, a C-terminal RING domain and sequence motifs facilitating its localizations in (NLS: nuclear localization signal; NoLS: nucleolar localization signal) and out (NES: nuclear export signal) of nucleus (Figures 1A,B). The interaction between MDM2 and p53 is made particularly strong by p53’s ability to bind MDM2 through multiple interfaces (Chi et al., 2005; Yu et al., 2006; Poyurovsky et al., 2010). Functioning as a E3 ubiquitin ligase, MDM2 serves as a constant quencher of p53 activity by mediating ubiquitination of p53 on its C-terminus to promote proteasome-mediated degradation (Haupt et al., 1997; Midgley and Lane, 1997). To release the strong clamp of MDM2, stress-induced signaling pathways use a variety of mechanisms to probe and prod between p53 and MDM2 to free p53. These mechanisms mainly lead to post-translational modifications (PTM) of p53, such as phosphorylation at serine 15/20/37/106 and threonine 18 to weaken p53-MDM2 interaction, and acetylation at C-terminal domain (CTD) lysine residues to prevent MDM2-mediated ubiquitination (Shieh et al., 1997; Unger et al., 1999; Nakamura et al., 2000; Rodriguez et al., 2000; Sakaguchi et al., 2000; Li et al., 2002; Hsueh et al., 2013). The relative significance of these PTM events has been a subject of debates. For example, lysine-to-arginine (KR) substitutions at multiple CTD acetylation sites significantly altered expression of p53 target genes but resulted in few abnormal phenotypes in mouse models (Krummel et al., 2005; Tang et al., 2008). A nonsense mutation at serine 15 was found to reduce p53-mediated transactivation but have little effect on p53’s interaction with MDM2 and its stability (Dumaz and Meek, 1999). These discrepancies can be attributed to functional regulation of individual PTM sites, crosstalk between PTM sites, other MDM2-mediated p53 PTM such as neddylation, and complexity surrounding p53-MDM2 hub to calibrate p53 activities (Lambert et al., 1998; Xirodimas et al., 2004; Laptenko et al., 2015). To ensure that p53 functions are only activated in a transient manner, MDM2 is transcriptionally induced by WT p53 to form a regulatory feedback loop (Barak et al., 1993). p53-mediated regulation of MDM2 likely contributes to a system capable of fine-tuning p53 functions.

Another way to activate p53 functions is through direct inhibition of MDM2. Among pathways reported to date, ARF-mediated MDM2 inhibition is the most well studied mechanism. ARF (or p14 in human and p19 in mouse) is encoded by the *CDKN2A* locus, which also encodes another tumor suppressor, p16INK4A (Figure 1C). ARF and p16INK4A are transcribed from two partially overlapped open reading frames and translated to two unrelated proteins. ARF activates p53 by directly interacting with MDM2 to inhibit its functions (Kamijo et al., 1998; Pomerantz et al., 1998). Mechanistically, two arginine rich domains (amino acids, or aa 1–14 and 82–101) of ARF predispose its localization to the nucleolus (Zhang and Xiong, 1999; Rizos et al., 2000). The N-terminal 1–14 motif interacts with the central region of MDM2, exposing its NoLS motif to sequester ARF-MDM2 complex in the nucleolus (Weber et al., 1999; Weber et al., 2000a; Lohrum et al., 2000). This phenomenon prevents MDM2 from exporting p53 into the cytoplasm for degradation, thus preserving p53 functions (Tao and Levine, 1999; Weber et al., 1999). In addition to the spatial restriction, ARF also stabilizes p53 through inhibiting MDM2’s ubiquitin-ligase activity (Honda and Yasuda, 1999; Midgley et al., 2000). Interestingly, several studies have demonstrated disconnections between nucleolar localization of ARF-MDM2 complex, p53 stabilization, and p53-mediated functions, implicating additional complexity surrounding this linear relationship between ARF, MDM2 and p53 (Llanos et al., 2001; Korgaonkar et al., 2002). Mirroring the feed-back mechanism between MDM2 and p53, WT p53 recruits histone deacetylases (HDAC) and polycomb group (PcG) proteins to repress ARF expression (Zeng et al., 2011).

### Factors Known to Function Through p53-MDM2-ARF Triangle

**FIGURE 2:**
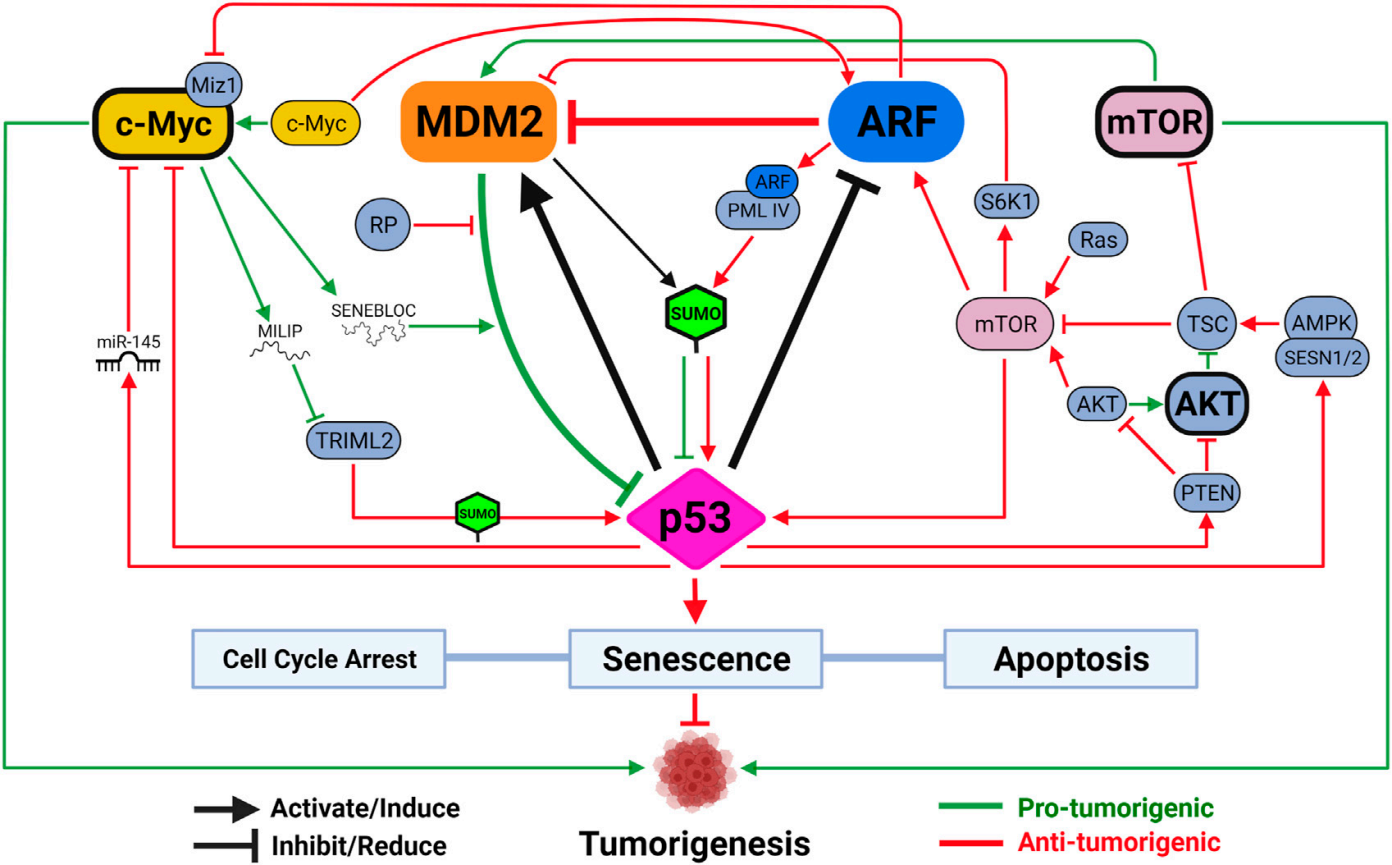
The functional triangle between p53, MDM2 and ARF. The MDM2-p53 duplex is considered the central hub controlling p53-mediated tumor-suppressive activities. Mechanisms disrupting MDM2-p53 interaction lead to p53 activation, which induces MDM2 expression through a negative feedback loop. ARF promotes p53 activation by inhibiting MDM2. Activated p53 reduces ARF expression through HDAC and PcG transcriptional repressors, and loss of p53 often leads to ARF induction. mTOR and c-Myc are two versatile signaling factors that mediate tumor-regulating mechanisms by engaging with all three members of the triangle. Both mTOR and c-Myc (unbolded) induce p53 activation when initially encountering DNA damage stress but are both capable of, upon overexpression or sustained activation (bolded), promoting tumorigenesis by inhibiting p53 activity. Created with BioRender.com.

Mechanisms regulating the expression and function of ARF, MDM2 and p53 have been extensively studied (See reviews by Maggi et al., 2014; Hafner et al., 2019; Klein et al., 2021). In the vast pool of regulators, a few unique players function through all three to control cancer development, such as cell proliferation factor mammalian target of rapamycin (mTOR) and oncogene c-Myc (Figure 2). In response to cellular stresses, p53 inhibits mTOR activity either by activating mTOR inhibitor the tuberous sclerosis (TSC)1/TSC2 complex through AMP-activated kinase (AMPK) and sestrin-1/2, or inducing transcription of phosphatase and tensin homolog (PTEN) to inhibit mTOR activator AKT (Stambolic et al., 2001; Feng et al., 2005; Budanov and Karin, 2008). A recent study demonstrated, using an acetylation-defective *p53-4KR* mouse model, that p53’s ability to suppress mTOR function is linked to distinctive tumor-suppressive activities independent of cell cycle arrest, senescence, and apoptosis (Kon et al., 2021). The ability of p53 to fine-tune mTOR activity has implications beyond tumor suppression. Recent studies showed that p53-regulated mTOR functions affect cells’ metabolic fitness during early development and dictate evolutionary advantages/disadvantages in our ancestors (Bowling et al., 2018; Gnanapradeepan et al., 2020).

As a negative feedback mechanism to integrate DNA damage response into cellular metabolism, mTOR activation increases p53 activity. In the event of PTEN loss, mTOR directly binds and phosphorylates p53 to promote senescence, a phenomenon previously known to be regulated by mTOR to counter DNA damage (Korotchkina et al., 2010; Jung et al., 2019). Miceli et al. (2012) showed that, in response to oncogenic Ras signaling or loss of TSC function, activated mTOR enhances translation of existing *ARF* mRNA to promote p53 activity and tumor suppression. In cases with loss of TSC function, mTOR also induces p53 activity by activating S6 kinase 1 (S6K1) to phosphorylate MDM2 and compromise its ability to move to the nucleolus (Lee et al., 2007; Lai et al., 2010). It is worth noting, however, that excessive activation of AKT/mTOR signaling results in p53 inhibition to promote tumorigenesis in some cancers due to AKT-mediated stimulation of MDM2 (Mayo and Donner, 2001). Combined inhibition of AKT/mTOR and MDM2 in these cancers, therefore, showed some promise as a therapeutic strategy (Kojima et al., 2008; Daniele et al., 2015). A novel pro-tumorigenic activity induced by mTOR-MDM2 pathway was recently described in tumor microenvironment (TME). Kamer et al. (2020) showed that lung cancer cells induce mTOR-dependent MDM2 translation in stromal cells, establishing a positive feedback loop to promote neighboring cancer cells’ metastatic potential. This mechanism was shown to be independent of stromal-p53, representing another dimension of mTOR’s tumor-promoting activity.

Endogenous c-Myc induces ARF expression and p53-dependent apoptotic programs upon initial response to DNA damage, but ultimately selects for spontaneous inactivation of ARF-MDM2-p53 pathway leading to tumorigenesis (Zindy et al., 1998; Eischen et al., 1999; Nieminen et al., 2013; Phesse et al., 2014). To suppress c-Myc-induced tumorigenesis, p53 can transcriptionally repress c-Myc directly through promoting histone deacetylation or indirectly through induction of microRNA (miR)-145 (Ho et al., 2005; Sachdeva et al., 2009). ARF directly interacts with c-Myc or its transcriptional cofactor Miz1 to inactivate pro-tumorigenic transcriptional programs and induce growth arrest and cell death even in the absence of p53 (Datta et al., 2004; Qi et al., 2004; Herkert et al., 2010). Two parallel pathways through MDM2 have also been described to sustain p53 activity to counter c-Myc’s pro-tumorigenic functions. In addition to ARF-MDM2 interaction, ribosomal protein (RP)-MDM2 interaction is also required to maximize p53 activity to inhibit c-Myc-induced tumorigenesis (Macias et al., 2010; Meng et al., 2015). Two recent studies demonstrated how c-Myc targets p53-MDM2-ARF tumor-suppressive axis by regulating two separate long noncoding RNAs (lncRNAs). Xu et al. (2020) identified SENEBLOC, a c-Myc-induced lncRNA involved in evasion of senescence by acting as a scaffold to increase association between p53 and MDM2, thus promoting p53 degradation. Another c-Myc responsive lncRNA, c-Myc-Inducible Long noncoding RNA Inactivating p53 (MILIP), was found to promote p53 turnover by reducing p53 SUMOylation through inhibiting tripartite-motif family-like 2 (TRIML2) (Feng et al., 2020). As TRIML2 has been found to influence cell fate decisions based on duration of p53-mediated response, the exact dynamic between c-Myc and p53 could dictate outcomes of c-Myc-induced tumorigenesis, including response to different therapies (Kung et al., 2015).

Interestingly, SUMOylation of p53 has been shown as a significant PTM mechanism through which MDM2 and ARF regulate p53 functions. Both MDM2 and ARF can mediate small ubiquitin-like modifier (SUMO)-1-mediated SUMOylation of p53 through their ability to target p53 to the nucleolus (Chen and Chen, 2003). Mechanistically, ARF interacts with a specific spliced variant of promyelocytic leukemia protein (PML), PML IV, to stabilize SUMO1-conjugating enzyme UBC9 in nuclear bodies (NB) to promote p53 SUMOylation and activation (Ivanschitz et al., 2015). ARF also mediates p53-independent functions by inducing SUMOylations of other targets, including NPM and MDM2 (Xirodimas et al., 2002; Tago et al., 2005). In human cells, MDM2-ARF complex, independent of their ability to relocate to nucleolus, also promotes SUMO-2/3-mediated SUMOylation of p53 to modulate its transcriptional activity (Stindt et al., 2011). As SUMOylation is emerging as a promising therapeutic target in cancer, its interaction with p53-MDM2-ARF pathway will be under increasing scrutiny (Kroonen and Vertegaal, 2021).

## The Curious Case Between MDM2 and p53

**FIGURE 3:**
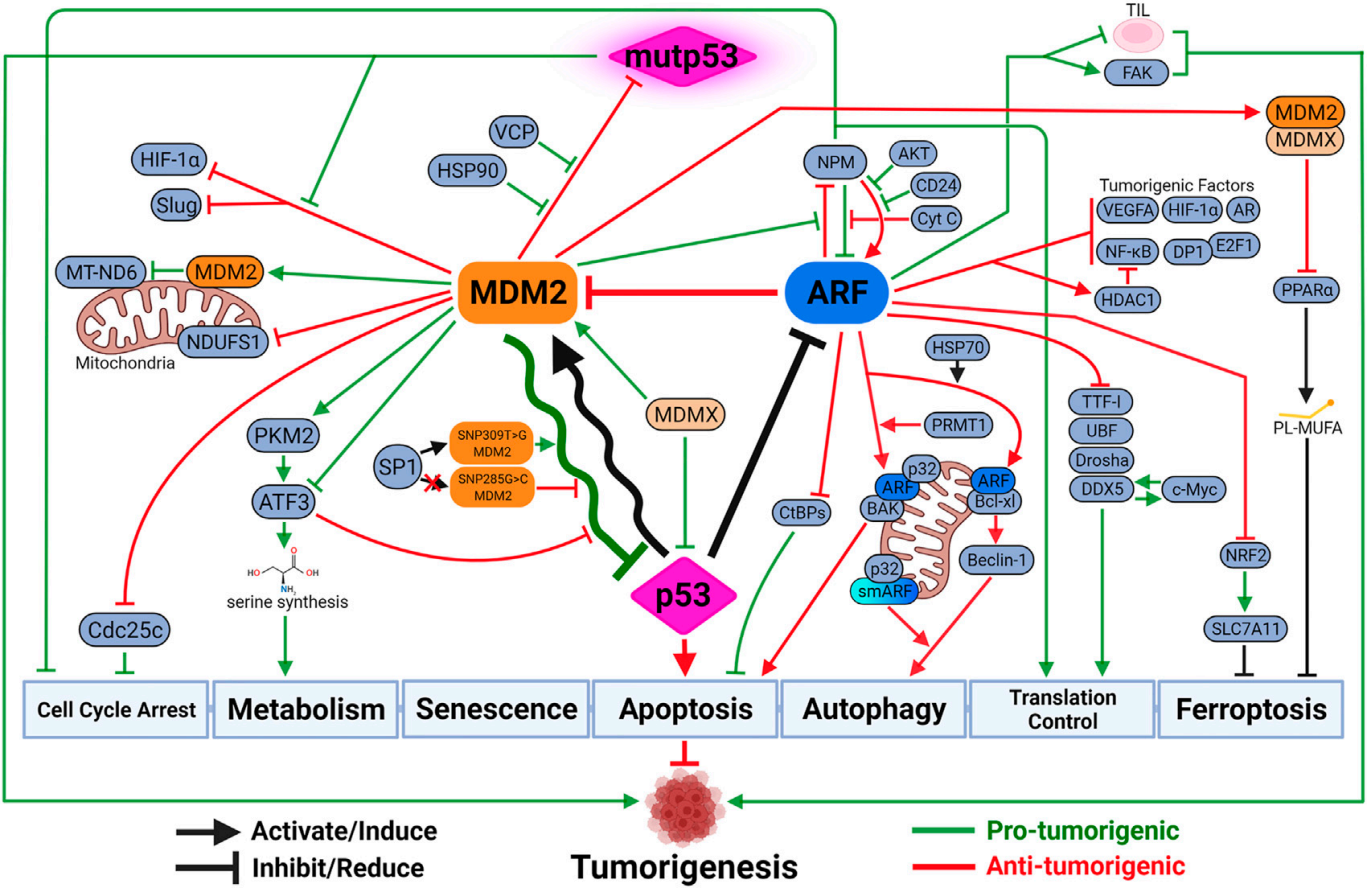
The updated look at the p53-MDM2-ARF functional complex. Both MDM2 and ARF mediate a variety of p53-independent functions to regulate tumorigenesis. These functions can be either synergistic with or antagonizing against p53-medaited tumor-suppressive activities. Due to the negative feedback loop, the oscillatory relationship between MDM2 and p53 dictates the intensity and duration of p53-mediated tumor-suppressive activities. The outcome of the oscillation depends on their respective expression and activity levels, which are influenced by genetic alterations such as SNP and mutations. Paradoxically, MDM2 sometimes exhibits anti-tumorigenic activity by inhibiting mutant p53 (mutp53) functions, inhibiting pro-tumorigenic factors upon p53 activation, or inducing other tumor-suppressive mechanisms like ferroptosis. ARF also possesses tumor-promoting capabilities when protecting cancer cells against specific types of cell death like anoikis, or exhausting functions of tumor-infiltrating lymphocytes (TIL) to promote cancer progression. Created with BioRender.com.

Evolutionarily, structural and functional features between MDM2 and p53 are highly conserved from multi-cellular eukaryotic organisms to mammals like mouse and human (Lane et al., 2010). It suggests a critical role of p53-MDM2 hub in consolidating diverse stress signaling pathways to determine cell fates. It is posited that one of the advantages of a biological central-hub like p53-MDM2 is ability to build functional complexity, including redundant, compensatory and feedback pathways, around it as needed (Levine, 2020). For example, DNA damage sensor activating transcription factor 3 (ATF3) activates p53 by preventing its degradation by MDM2, which in turn mediates ubiquitination and degradation of ATF3 to inactivate p53 (Yan et al., 2005; Mo et al., 2010) (Figure 3).

### Tug-of-War Oscillatory Relationship Between p53 and MDM2

Part of this complexity can be attributed to oscillatory relationship between MDM2 and p53. The regulatory feed-back loop, which allows p53 to upregulate its own inhibitor MDM2, is meant to suppress lethal p53 activity in normal cells and during development (de Oca Luna et al., 1995; Jones et al., 1995; Ringshausen et al., 2006). As the result, the mutual relationship between p53 and MDM2 can dictate physiological homeostasis outside the context of cancer development. For example, normal aging process relies on a balanced p53-MDM2 signaling network, of which dysregulations lead to premature aging or pathological conditions (Wu and Prives, 2018). Interestingly, it has been shown that p53 oscillates faster in mouse and rat cells than in cells from human, monkey, or dog. It is suggested that faster p53 oscillations in mouse might be due to subtle changes in p53 RE of mouse MDM2, leading to altered expression of MDM2 and a stronger feedback loop signal (Stewart-Ornstein et al., 2017). These variations could have significant consequences, due to the connected nature between p53-MDM2 oscillations and transcriptional regulations of p53 target genes (Hafner et al., 2017). In cancer cells, it is suggested that oscillatory p53-MDM2 activity is dictated by the intensity of stress signals, as well as expression level of p53 and MDM2 upon encountering stresses (Lev Bar-Or et al., 2000; Lahav et al., 2004; Ma et al., 2005). This hypothesis was demonstrated in cells with a single nucleotide polymorphism (SNP) of *MDM2* (SNP309T>G; rs2279744) that results in higher level of MDM2 and inhibition of coordinated p53-MDM2 oscillation (Hu et al., 2007). Higher expression of SNP309T>G *MDM2* is due to increased affinity of its transcriptional activator Sp1, which preferentially responds to estrogen signaling (Phelps et al., 2003; Bond et al., 2004). As the result, SNP309T>G *MDM2* is found to associate with accelerated tumor formation in a gender-specific and hormone-dependent manner (Bond et al., 2006; Post et al., 2010). In contrast, another *MDM2* SNP that is only 24 base pairs upstream of SNP309, SNP285G>C (rs117039649), disrupts an Sp1-binding site to decrease MDM2 expression. SNP285G>C is exclusively found in Caucasians and, when coexisting with SNP309T>G, associates with reduced risks for female reproductive cancers (Knappskog et al., 2011). Interestingly, increased longevity was observed in females with SNP309T>G *MDM2* if they didn’t suffer from cancer diagnoses (Gross et al., 2014). This phenomenon could be attributed to higher MDM2 expression leading to suppressed p53 stress response in stem cell populations. Similar paradoxical regulations between cancer susceptibility and metabolic fitness have been linked to other SNPs in p53-MDM2 pathway, including Proline72Arginine (P72R; rs1042522) and Proline47Serine (P47S; rs1800371) of p53 (Kung et al., 2015; Jennis et al., 2016; Kung et al., 2016; Kung et al., 2017; Gnanapradeepan et al., 2020). It remains to be seen if these SNPs also impact the fine balance separating tumorigenesis and homeostasis through regulating oscillatory activity between p53 and MDM2.

### Tumor-Suppressive Functions of MDM2

Negative feedback activity is not the only outcome for p53-mediated MDM2 induction. For example, in non-small-cell lung cancer (NSCLC), WT p53 suppresses cancer metastasis by facilitating MDM2-mediated degradation of metastatic promoter Slug (Wang et al., 2009). More recently, p53-induced MDM2 was found to slow down cell cycle progression by promoting degradation of mitosis-promoting factor Cdc25C, which is also a transcriptionally-repressed target of p53 (Clair et al., 2004; Giono et al., 2017). Considering that MDM2 can potentially reach many targets through its E3 ligase activity, the consequence of p53-induced MDM2 expression could be tumor-promoting or tumor-suppressing depending on the cell type and surrounding factors. Hypoxia-inducible factor 1-alpha (HIF-1α) is another pro-tumorigenic factor that can be degraded by MDM2 in a p53-dependent manner (Ravi et al., 2000). Interestingly, mutant p53 (mutp53) was found to exert its tumor-promoting activity by dissociating HIF-1α from MDM2, leading to HIF-1α upregulation (Kamat et al., 2007). The aforementioned metastatic promoter Slug can also be stabilized in the presence of mutp53, which represses MDM2 through inhibiting p73-mediated MDM2 transactivation (Wang et al., 2009). MDM2 can also mediate the degradation of mutp53 and keep it at basal levels in cancer cells (Haupt et al., 1997; Terzian et al., 2008). Since mutp53 is incapable of inducing MDM2 expression to complete the feedback loop, it can be stabilized through interacting with factors disrupting mutp53-MDM2 complex, such as heat shock protein (HSP) chaperones HSP90 and valosin-containing protein (VCP), to execute pro-tumorigenic activities (Midgley and Lane, 1997; Peng et al., 2001; Li et al., 2011a; Wang et al., 2021). The recent finding demonstrating functional plasticity of mutp53 between tumor-suppressor and tumor-promoter revealed new dimension in p53 biology, including MDM2’s potential role in regulating mutp53 activities (Kadosh et al., 2020).

### MDM2 Functions Antagonizing Against or Synergizing With p53 Activity

Not surprisingly, functional complexity evolving around MDM2 has drawn increasing attention in recent years (Klein et al., 2021). Many MDM2-mediated functions have been shown to operate independent of p53 but demonstrate capacities to synergize or antagonize p53-mediated pathways. It is well established that mitochondria p53 confers important biological functions, both in mediating mitochondria-based apoptosis and regulating mitochondrial respiration to control cancer development (Leu et al., 2004; Murphy et al., 2004; Matoba et al., 2006). It has been shown that upon oxygen deprivation, a fraction of MDM2 localizes to the mitochondria in p53-independent manner, inhibits mitochondrial respiration by reducing complex I subunit NADH-dehydrogenase 6 (MT-ND6), enhances reactive oxygen species (ROS) production, and promotes cancer cell migration and invasion (Arena et al., 2018). Interestingly, however, a more recent study showed that cytosolic MDM2, by sequestering mitochondria stabilizer NADH:ubiquinone oxidoreductase 75 kDa Fe-S protein 1 (NDUFS1), induces ROS to promote apoptosis (Elkholi et al., 2019). It remains to be seen what regulatory mechanisms differentiate MDM2’s ability to promote or inhibit tumorigenesis through regulating mitochondria functions.

Under metabolic stress, p53 can support cancer cell proliferation and survival by mediating metabolic reprogramming. One such mechanism manifests in the event of serine deprivation, during which p53 activates the synthesis of serine and glutathione, preserving anti-oxidant activity to reduce oxidative stress (Maddocks et al., 2013). MDM2 is also capable of triggering serine synthesis pathway upon serine starvation, independent of p53, through PKM2 (pyruvate kinase 2)-mediated recruitment to chromatin to facilitate a ATF3/4-mediated transcriptional program (Riscal et al., 2016). It will be interesting to dissect the regulatory mechanisms distinguishing this pathway and aforementioned p53-MDM2-ATF3 feedback loop upon DNA damage. In contrast to the pro-tumorigenic functions in response to serine depletion, MDM2 and p53 can also converge on anti-tumorigenic pathways, such as an iron-dependent form of nonapoptotic cell death, ferroptosis (Stockwell et al., 2017). Jiang et al. (2015) first showed that ferroptosis is a critical mechanism for p53-mediated tumor suppression. Their argument relies on the fact that an acetylation-defective p53 mutant, p53(3KR), retains ferroptosis-inducing and tumor-suppressing capabilities despite failing to promote cell-cycle arrest, senescence and apoptosis. This is supported by the discovery that a African-centric, cancer-predisposing p53 polymorphism P47S has impaired ability to promote ferroptosis by inducing levels of antioxidants coenzyme A (CoA) and glutathione (GSH) (Jennis et al., 2016; Leu et al., 2019). Interestingly, Liu et al. (2017a) showed that mutp53 can sensitize some cancer cells to ferroptosis by inhibiting the cystine/glutamate antiporter and glutathione biosynthesis, providing another mechanistic basis for p53 reactivation therapy. A recent finding by Venkatesh and colleagues showed that MDM2, working in a complex with Murine Double Minute X (MDMX), facilitates ferroptosis through altering cellular lipid profiles and preventing anti-oxidant responses (Venkatesh et al., 2020). Interestingly, MDM2’s positive regulation of ferroptosis may not be entirely p53-independent. It was shown in some cancer cells, stabilization of p53 by MDM2 inhibitor Nutlin-3 delays ferroptosis induced by cystine deprivation (Tarangelo et al., 2018). This phenomenon was found to depend on the p53 target gene p21, but the underlying mechanism is still unclear. Whether this effect is dictated by the p53-MDM2 relationship and sensitive to other forms of MDM2 regulations requires more extensive studies.

### MDMX Regulates MDM2-p53 Functions

Despite the recent discovery of its role collaborating with MDM2 to promote ferroptosis, MDMX (also known as MDM4) is mostly considered a pro-tumorigenic factor like MDM2 (Ramos et al., 2001). Similar to MDM2, MDMX can directly inhibit p53 functions through binding between their N-terminal domains (Shvarts et al., 1996; Danovi et al., 2004). The functional significance of MDMX-mediated p53 inhibition was demonstrated in a transgenic mouse model where loss of *Trp53* rescues embryonic lethality caused by *Mdm4* deletion (Parant et al., 2001).

In addition to inhibiting p53 functions directly, MDMX’s contribution to tumorigenesis could also be attributed to its ability to enhance MDM2 activity. Although MDMX does not possess intrinsic E3 ligase activity, early investigations showed that it can form heterodimers with MDM2 to increase MDM2 stability and promote MDM2-mediated ubiquitination and degradation of p53 (Sharp et al., 1999; Gu et al., 2002; Linares et al., 2003). Subsequent studies revealed that the C terminus of MDMX not only is required for the formation of MDM2/MDMX heterodimer, but also is able to rescue E3 ligase activity lost in MDM2 containing E3-defective C-terminal mutations (Singh et al., 2007; Uldrijan et al., 2007). Moreover, it was later shown that MDM2/MDMX heterodimer is a more efficient E3 ligase of p53 compared to MDM2 homodimer, suggesting that it could be the predominant form in cells regulating p53 functions (Kawai et al., 2007; Huang et al., 2011; Leslie et al., 2015). The functional relationship between p53, MDM2 and MDMX is evolutionarily conserved, highlighting their importance in maintaining physiological homeostasis (Momand et al., 2011; Dolezelova et al., 2012; Coffill et al., 2016). Interestingly, MDMX’s E3 ligase activity was found to be retained in some invertebrates and can be restored in the human ortholog by substituting a few amino acids (Iyappan et al., 2010; Coffill et al., 2016). It suggests that functions of MDMX have evolved to adapt to increasing environmental complexities, potentially through its interactions with MDM2 and p53 (Tan et al., 2017).

## The Expanding Universe Between ARF and p53

The importance of ARF in tumor suppression was readily demonstrated in mouse models. Transgenic mice homozygous for *Arf* loss (*p19Arf* ^null^) succumb to spontaneously-developed tumors of a wide spectrum, including sarcomas, lymphomas, carcinomas and nervous system cancers, within a year (Kamijo et al., 1997; Kamijo et al., 1999). Functional distinctions between Arf and p16Ink4a were also evident in mice, in which loss of both tumor suppressors results in significantly more severe phenotypes (Sharpless et al., 2004). The picture of ARF’s functional significance in human cancers is murkier. Despite the loss of *CDKN2A* being the most frequent genetic event second only to p53 mutations, it is difficult to dissect the respective contributions of p14ARF and p16INK4A to tumor suppressions in human. Despite the limitations, ARF-specific alterations, both proteogenomic and epigenetic, have been found in a wide variety of human cancers including central nervous system, bladder, colon, breast, prostate, ovarian, liver, gastric, lung, head and neck, as well as hematologic cancers. It unequivocally suggests that ARF plays a critical role in tumor suppression (Maggi et al., 2014; Inoue and Fry, 2018).

### ARF Mediates p53-independent Tumor Suppression

The first indication that ARF possesses p53-independent functions was revealed when *Arf*/*Trp53* double-knockout and *Arf*/*Trp53*/*Mdm2* triple-knockout mice developed tumors of distinctive origins compared to *Arf* ^null^ or *Trp53* ^null^ mice (Weber et al., 2000b). A similar conclusion has been reached in human cancers through demonstrations that 1) ARF is capable of suppressing tumor progression in the absence of active p53; and 2) loss of ARF often synergizes with dysregulated p53 to promote tumorigenesis (Eymin et al., 2003; Sandoval et al., 2004; Muniz et al., 2011; Forys et al., 2014). Moreover, high prevalence of *TP53* and *CDKN2A* co-inactivation has been identified in a variety of cancers, including glioblastoma, hepatocellular carcinoma, lung cancer, pancreatic cancer, bladder cancer, and triple-negative breast cancer (TNBC) among others (Cerami et al., 2012; Gao et al., 2013). It further implicates ARF’s p53-independent tumor-suppressive functions, although more studies are needed to define ARF’s roles apart from those of p16INK4A in each cancer type.

### ARF and NPM

Several p53-independent mechanisms have been associated with ARF-mediated tumor suppression. In response to hyperproliferative signals, ARF sequesters pro-tumorigenic nucleophosmin (NPM) in nucleolus to promote cell cycle arrest (Brady et al., 2004). The relationship between ARF and NPM appears to be mutual, but the impact of NPM on ARF function is context dependent. The interaction between ARF and NPM can preserve ARF function by preventing its degradation, while overexpressed NPM or cancer-associated NPM mutants have been shown to inhibit ARF functions by restricting its ability to translocate between nucleolus and cytoplasm (Bertwistle et al., 2004; Kuo et al., 2004; Korgaonkar et al., 2005; Colombo et al., 2006; Moulin et al., 2008). This unique relationship between ARF and NPM not only can be disrupted by MDM2, but is also sensitive to other factors, including AKT, cytochrome c, and CD24 to regulate p53-dependent and -independent functions of ARF (Brady et al., 2004; Hamilton et al., 2014; Wang et al., 2015; González-Arzola et al., 2020). ARF-NPM interaction also regulates ARF’s ability to promote apoptosis independent of p53 (Hemmati et al., 2002; Eymin et al., 2003).

### ARF Functions at the Mitochondria

ARF-mediated apoptosis relies on ARF’s ability to localize to mitochondria, and is regulated by the interaction between ARF and mitochondrial protein p32 (Itahana and Zhang, 2008). A recent study elucidated the underlying mechanism by showing that, under genotoxic stresses, PRMT1 (protein arginine methyltransferase 1) methylates arginine residues within the NLS/NoLS of ARF, resulting in the release of ARF from NPM and increased interaction between ARF and p32 (Repenning et al., 2021). Mitochondria-bound ARF induces apoptosis by activating BAK instead of BAX, suggesting a tightly-regulated process controlling ARF-mediated apoptosis in the absence of p53 (Müer et al., 2012).

BAK-dependent apoptosis is not the only anti-tumorigenic mechanism that ARF induces once reaching mitochondria. With the help of heat shock protein 70 (HSP70), ARF travels to mitochondria, interacts with Bcl-xl, disrupts the Bcl-xl/Beclin-1 complex to release autophagic factor Beclin-1 to induce autophagy (Abida and Gu, 2008; Pimkina et al., 2009; Pimkina and Murphy, 2011). This ability to induce autophagic cell death from mitochondria is interestingly shared by the full-length ARF and a shorter isoform of ARF, smARF (short mitochondrial ARF) (Reef et al., 2006; Budina-Kolomets et al., 2013). The contribution of smARF to tumor suppression remains controversial due to its low abundance and unstable nature, but its physiological function has been clearly demonstrated in a mouse model where expression of smArf significantly rescued developmental defects of *Arf*-null mice (van Oosterwijk et al., 2017). Interestingly, p32 was also found to interact with and stabilize smARF, raising the question that if p32 serves as an arbitrator at mitochondria to regulate both apoptosis and autophagy triggered by ARF and smARF (Reef et al., 2007). Both mitochondrial p32 and ARF have been shown to control metabolic programming between oxidative phosphorylation and glycolysis (Fogal et al., 2010; Christensen et al., 2014; Gotoh et al., 2018; Koss et al., 2020). Since the metabolic state of mitochondria is important in regulating both cancer cell-intrinsic and -extrinsic mechanisms, ARF’s influence on tumor metabolism independent of p53 warrants further investigation (Xiao et al., 2019; Yao et al., 2019).

### ARF and Translational Control

By virtue of being an nucleolar protein, ARF exerts p53-independent tumor suppression through regulating ribosome biogenesis, ribosomal RNA (rRNA) processing and translation (Sugimoto et al., 2003; Cottrell et al., 2020). ARF-mediated regulation of NPM is also involved in this process, as ARF reduces function and stability of NPM required for ribosome biogenesis (Itahana et al., 2003; Apicelli et al., 2008; Maggi et al., 2008). ARF has been shown to regulate ribosomal functions and translation through many other mechanisms, such as directly interacting with rRNA promoter, blocking nucleolar import of RNA polymerase I transcription termination factor (TTF-I), inactivating rRNA transcriptional factor upstream binding factor (UBF), downregulating rRNA-processing enzyme Drosha, and limiting nucleolar localization of RNA helicase DDX5 (Ayrault et al., 2004; Lessard et al., 2010; Saporita et al., 2011; Kuchenreuther and Weber, 2014). Interestingly, ARF’s ability to interact with DDX5 also prevents interaction between DDX5 and c-Myc, disrupting a oncogenic positive feedback loop that increases c-Myc-mediated transcription and cell transformation (Tago et al., 2015).

### Other p53-independent Tumor-Suppressive Functions of ARF

The reach of ARF’s p53-independent, tumor-suppressive functions extends to many other cancer-related pathways. To inhibit pro-tumorigenic machineries, ARF blocks E2F1’s transcriptional activity by interacting with E2F1 and E2F1 cofactor DP1; inhibits HIF-1α-mediated transcription by sequestering HIF-1α in nucleolus; attenuates NF-κB functions by recruiting transcriptional repressor histone deacetylase 1 (HDAC1) to NF-κB subunit RelA/p65; interacts with androgen receptor to repress its transactivation activity; and suppresses translation of tumor angiogenic factor vascular endothelial growth factor A (VEGFA) (Eymin et al., 2001; Fatyol and Szalay, 2001; Martelli et al., 2001; Datta et al., 2002; Rocha et al., 2003; Datta et al., 2005; Kawagishi et al., 2010; Lu et al., 2013). ARF also interacts directly with anti-apoptotic transcriptional corepressor C-terminal binding protein 1 (CtBP1) and 2 (CtBP2), leading to their degradation and p53-independent apoptosis (Paliwal et al., 2006; Kovi et al., 2010).

ARF also promotes other anti-tumorigenic mechanisms. In response to DNA damage, ARF induces both p53-dependent and -independent senescent response, the later through ATM/ATR/CHK signaling pathway (Eymin et al., 2006; Carlos et al., 2013; Monasor et al., 2013). Another binding partner of ARF is nuclear factor erythroid 2-related factor 2 (NRF2), which transcriptionally activates *SLC7A11*, a component of the cystine/glutamate antiporter complex. By importing cystine, SLC7A11 promotes biosynthesis of antioxidant glutathione (GSH), resulting in reduction of ROS and lipid peroxides (DeNicola et al., 2011; Ye et al., 2014). By interacting with NRF2, ARF inhibits SLC7A11 expression to promote lipid peroxidation and trigger ferroptosis (Chen et al., 2017). With the list of regulated cell death mechanisms ever-increasing, more p53-independent tumor-suppressive pathways induced by ARF could be discovered in the very near future (Tang et al., 2019).

### Co-Inactivation of p53 and ARF

Considering the plethora of p53-independent pathways described for ARF-mediated tumor suppression, there is surprisingly few mechanistic studies conducted in cancers with co-inactivation of p53 and ARF. Forys et al. used both mouse embryonic fibroblast (MEF) and human TNBC cell lines to show that co-inactivation of p53 and ARF induces an pro-tumorigenic signaling signature that includes induction of interferon-β (IFN-β) and activation of signal transducer and activator of transcription 1 (STAT1) (Forys et al., 2014). In a Eµ-Myc-driven lymphoma mouse model recapitulating late-stage p53 inactivation, Klimovich et al. (2019) showed that loss of ARF confers resistance to p53 restoration in established lymphoma. This result suggests that co-inactivation of p53 and ARF not only exacerbates tumorigenesis, but also compromises efficacy of p53 reactivation therapy. On the other hand, evidence is emerging to link co-inactivation of p53 and ARF to novel therapeutic opportunities. Co-deletion of *TP53* and *CDKN2A* was recently linked to gastric premalignancy and cancer progression mediated by dietary carcinogens (Sethi et al., 2020). Despite being a malignancy-driving event, co-deletion of *CDKN2A* following p53 inactivation also induces replication stress and sensitizes cancer cells to DNA damage response inhibitors. Since deletion of *CDKN2A* in this study didn’t distinguish between p16INK4A and ARF, the exact contribution of ARF needs to be further studied. The same caveat is applied to the same group’s another study in esophageal squamous cell carcinoma, where they found that *Trp53*/*Cdkn2a* loss synergizes with transcription factor Sox2 to promote chromatin remodeling, enhance Stat3 functions, activate endogenous retroviruses, and induce double-stranded RNA expression and dependence of RNA editing enzyme ADAR1 (Wu et al., 2021). Implication of this study could inform new strategies to develop therapies against cancers that display similar characteristics (p53/ARF co-inactivation and ADAR1 dependency), such as TNBC (Forys et al., 2014; Kung et al., 2021).

### Tumor-Promoting Functions of ARF

It is worth noting that ARF’s p53-independent functions have been suggested to promote cancer progression under certain circumstances. Overexpression of ARF in cancer has been mostly considered a byproduct of p53 mutation due to the previously mentioned negative feedback loop, and generally correlated with better prognosis (Kamijo et al., 1998; Silva et al., 2001; Song et al., 2014). Several studies, however, have provided mechanistic insights regarding how ARF’s presence might promote progression of some cancers. Humbey et al. (2008) first described, in a mouse model, that overexpression of ARF protects Eµ-Myc-driven lymphoma by inducing autophagy in response to nutrient starvation. Their data suggests that ARF, in a tumor-type specific manner, controls the switch between cyto-toxic and cyto-protective effects of autophagy in response to metabolic stress. Another cell-intrinsic pro-survival function of ARF was shown in spreading cancer cells in which ARF interacts with focal adhesion kinase (FAK) to stabilize cytoskeleton structure and protect cells from anoikis, a form of programmed cell death during cell detachment (Vivo et al., 2017). A recent study also shed some light on a cell-extrinsic mechanism through which ARF behaves as a tumor promoter. Koss et al. showed that during cancer development, tumor-induced metabolic stress suppresses function of epigenetic modifier enhancer of zeste homolog 2 (EZH2), leading to upregulation of ARF. Without affecting p53 function, ARF promotes mitochondrial dysfunction and metabolic exhaustion of tumor-infiltrating lymphocytes (TIL), resulting in cancer progression (Koss et al., 2020). More ARF-mediated pathways, p53-dependent and -independent, are expected to be identified in regulating TME functions.

## Therapeutic Opportunities to Target p53-MDM2-ARF Triangle

**FIGURE 4:**
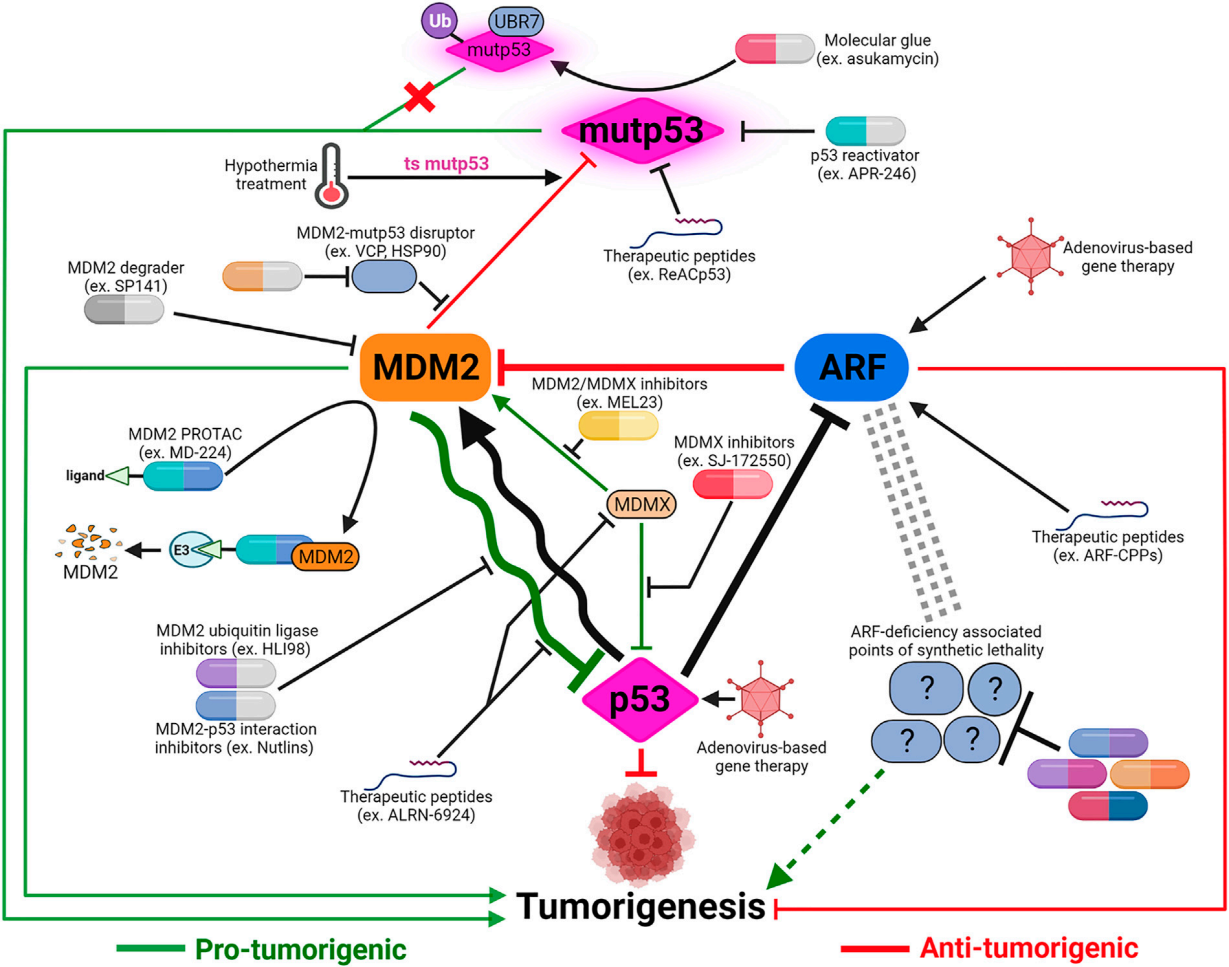
Cancer therapeutic strategies targeting the p53-MDM2-ARF signaling axis. The majority of p53-based therapeutic strategies are designed to reactivate mutant p53 or inhibit MDM2-p53 and MDM2/MDMX-p53 interactions using small molecules. Development of alternative strategies, such as applying hypothermal therapy to induce MDM2-mediated degradation of temperature-sensitive mutp53, or utilizing pan-MDM2 inhibitors, are meant to maximize anti-tumor potency depending on the context of MDM2-p53 relationship. In addition to small molecules, other modalities including therapeutic peptides and proteolysis targeting chimera (PROTAC) are also being explored to target both MDM2’s p53-dependent and -independent functions. The realization of ARF’s many p53-independent functions and the functional significance of p53-ARF co-inactivation in cancers necessitates development of ARF-based therapies. Virus-mediated gene therapy and therapeutic peptides are potential ways to restore ARF functions. Identification of synthetic lethality associated with ARF deficiency can uncover novel therapeutic targets to compensate for ARF loss and potentially synergize with p53-targeting treatments. Created with BioRender.com.

Potential therapies to activate p53 command the most attention in development of drugs targeting p53-MDM2-ARF network. Direct restoration of WT p53 expression using intra-tumoral injection of p53-delivering adenovirus has been used to treat cancers in China since 2003 (Xia et al., 2020). Extreme cautiousness towards gene therapy in general and p53-targeting gene therapy in particular casts a cloud over when this treatment will become clinically available worldwide. In contrast, tremendous amount of efforts have been devoted to develop pharmacological activators of p53, including activators of WT p53 and re-activators of mutp53 to restore p53 functions (Nguyen et al., 2017). Other than PRIMA-1^MET^ (also called APR-246), a small-molecule mutp53 re-activator, most p53-targeting drugs in active clinical development/trials are small molecules that stabilize WT p53 by interrupting p53-MDM2 interaction or inhibiting MDM2’s ubiquitin ligase activity (Figure 4).

### Therapeutic Peptides for p53 Activation

Although small molecules still dominate the drug discovery landscape, other modalities have been explored as p53 activators, such as therapeutic peptides (Marqus et al., 2017). Small peptides derived from N-terminal MDM2-binding domain of p53 were shown to induce p53-mediated anti-tumorigenic activities more than 20 years ago (Böttger et al., 1997; Kanovsky et al., 2001). Efforts to apply p53/MDM2-targeting therapeutic peptides for cancer treatment culminated in the development of a p53-derived stapled peptide, ALRN-6924. ALRN-6924 exhibits dual MDM2/MDMX inhibitory activities and has shown promise in preclinical studies and early-stage clinical trials to halt progression of cancers bearing WT p53 (Carvajal et al., 2018; Pairawan et al., 2021; Saleh et al., 2021). A recent study showed that ALRN-6924 induces inflammatory response in melanoma to alter TME and overcome tumor immune evasion, suggesting its potential utility in combination with immunotherapy (Zhou et al., 2021a). Therapeutic peptides also have potential to treat cancers with mutp53. Soragni et al. (2016) showed that a cell-penetrating peptide (CPP) derived from DNA binding domain of p53, ReACp53, inhibits mutp53 aggregation and rescues WT-like p53 functions in high-grade serous ovarian carcinomas. The concept of using targeted protein degradation (TPD) to treat cancers with mutp53 is also being explored (Dale et al., 2021). Isobe et al. (2020) identified a small molecule, asukamycin, that serves as a “molecular glue” linking mutp53 with E3 ubiquitin ligase UBR7. Interestingly, however, that instead of degrading mutp53, treatment of asukamycin results in non-proteolytic ubiquitination and activation of mutp53 to promote cell death in TNBC cells.

### Novel Therapeutic Strategies Targeting p53-MDM2 Hub

Despite the large number of candidate drugs at different stages of preclinical/clinical development, no MDM2-p53 antagonist has been approved for cancer treatment due to challenges regarding efficacy and undesired toxicity (Zanjirband and Rahgozar, 2019; Mullard, 2020). Other than identifying more drug candidates based on the similar concept, further understanding of intricate relationship between p53 and MDM2 could provide valuable insights. As mentioned previously, most MDM2-p53 antagonists were designed to disrupt N-terminal binding between MDM2 and p53 or inhibit MDM2’s ubiquitin ligase activity mediated through its C-terminal RING domain. A single residue in central zinc finger domain, cysteine 305, was shown to control p53 function through interaction with RP (Lindström et al., 2007; Macias et al., 2010; Meng et al., 2015). In a mouse model (*Mdm2* ^*C305F*^) carrying this human cancer-associated single mutation of MDM2, it was shown that RP-MDM2-p53 pathway plays important roles in lipid metabolism and cells’ response to metabolic stress (Liu et al., 2014; Liu et al., 2017b). These findings support increasing effort to develop therapies targeting zinc finger domain of MDM2, especially in the context of exploiting metabolic vulnerabilities associated with MDM2-p53 pathway. Multiple mouse models have also been utilized to suggest delicate distinctions between MDM2’s ubiquitin ligase activity and ability to control p53 function. Two separate mutants of *Mdm2* (*Y487A*–Y489A in human; *I438K*–I440K in human) have been demonstrated to partially restrain p53 response to DNA damage despite losing its ability to promote p53 degradation (Tollini et al., 2014; Humpton et al., 2021). Interestingly, while *Mdm2* ^*Y487A*^ causes no developmental defect yet promotes p53-dependent mortality in response to sub-lethal stress in adult mice, *Mdm2* ^*I438K*^ leads to embryonic lethality but is tolerated when only switched on in adult mice to allow enhanced p53 response to DNA damage. These confusing discrepancies reflect a delicate balance in p53-MDM2 relationship. How to therapeutically target this equilibrium in order to control p53 dynamics could be the key to achieve balance between maximum efficacy and minimum toxicity (Purvis et al., 2012).

Another potentially useful approach is to stratify patients based on predicted response to MDM2-based therapies. It has been shown that sensitivity of cancer cells to MDM2 inhibitors could be predicted by gene signatures containing subsets of p53 target genes (Jeay et al., 2015; Ishizawa et al., 2018). Applicability of this approach remains to be seen, depending on its ability to model oscillatory relationship between p53 and MDM2. In cancer cells with mutp53, MDM2’s pro-tumorigenic potential might be outweighed by its ability to suppress gain-of-function oncogenic activity of mutp53. This was recently demonstrated in pancreatic ductal adenocarcinoma, in which pharmacologic inhibition of valosin-containing protein (VCP) promotes MDM2-mediated degradation of mutp53 and cell death (Wang et al., 2021). This concept could be applied to 1) inhibit other factors disrupting MDM2-mutp53 interaction, such as HSP90 and BAG2 (Li et al., 2011b; Yue et al., 2015); or 2) tip the dynamics of MDM2-mutp53 interaction towards p53 degradation to amplify MDM2’s anti-tumorigenic functions in cancers possessing mutp53 (Yang et al., 2019). Intrinsic characteristics of mutp53 could also dictate the functional consequence of p53-MDM2 interaction. A recent study demonstrated that some temperature sensitive (ts) mutp53, such as R282W and A138V, are resistant to MDM2-mediated degradation despite their ability to induce MDM2 upon reactivation. This result predicts favorable outcome of p53 reactivation in cancers possessing ts mutp53, and rationalizes including hypothermia-based treatment as part of cancer therapeutic strategy (Lu et al., 2021).

### Potential Therapies Targeting Pan-MDM2 Functions

As more p53-independent functions of MDM2 are discovered, more efforts are devoted to identifying therapies targeting pan-MDM2 functions instead of MDM2-p53 interaction. A variety of small molecule inhibitors were identified to induce MDM2 auto-ubiquitination and degradation, or inhibit its interactions with non-p53 binding partners (Wang et al., 2014; Burgess et al., 2016; Singh et al., 2016; Xu et al., 2016; Nguyen et al., 2017). These inhibitors not only provide dual advantages inhibiting both p53-dependent and -independent functions of MDM2, but also demonstrate critical roles of MDM2 regulators and cofactors such as Nuclear Factor of Activated T cell (NFAT1), and X-Linked Inhibitor of Apoptosis (XIAP) (Gu et al., 2016; Gu et al., 2018; Wang et al., 2019). Inhibition of p53-independent functions of MDM2 also contributes to activities of established chemotherapeutic agents, including Adriamycin and Nilotinib (Ma et al., 2000; Zhang et al., 2014). Increasing knowledge in this field will facilitate repurposing and tailoring existing therapies towards cancers that can benefit from MDM2-targeting interventions.

TPD strategy has also been applied to develop MDM2-targeting therapies. A first-in-class MDM2 degrader using proteolysis targeting chimera (PROTAC) concept, MD-224, was shown to be highly potent in inducing MDM2 degradation and achieving durable tumor regression *in vivo* (Li et al., 2019). MD-224 consists of a modified MDM2 inhibitor conjugated with a small-molecule ligand (lenalidomide) of an E3 ligase (cereblon) degradation system. Interestingly, since MDM2 is an E3 ligase itself, MDM2-recruiting PROTAC are being developed to target itself and other pro-tumorigenic proteins to maximize p53-dependent and -independent effects in tumor suppression (Hines et al., 2019; He et al., 2021).

### MDMX-Targeting Therapeutic Approaches

MDMX has emerged as a viable target for cancer therapy, both for its own ability to inhibit p53 and its role in the MDM2/MDMX complex, especially in cancers where amplification of MDMX is more prevalent than MDM2 (Gembarska et al., 2012; Burgess et al., 2016). The highly homologous yet non-identical sequence comparison between MDM2 and MDMX (>50% identical amino acid sequence in both N-terminal p53-binding and C-terminal RING domains) provides opportunities to target either MDMX specifically or the MDM2/MDMX complex. A series of molecules have been identified through MDMX-specific screens, including imidazoline derivative SJ-172550 that competes with MDMX to release functional p53 (Reed et al., 2010). SJ-172550 and other molecules subsequently identified through this approach have shown anti-tumorigenic effects and more importantly, abilities to synergize with MDM2-specific inhibitors (Reed et al., 2010; Wang et al., 2011; Wang and Yan, 2011; Karan et al., 2016). MDMX-targeting inhibitors have also been identified through indirect discoveries. Originally found to reduce proto-oncogene Survivin expression, camptothecin analogue FL118 was shown to activate p53 by promoting degradation of MDMX (Ling et al., 2014). Hsp90 inhibitor 17AAG was also found to be a potent MDMX degrader and synergize with MDM2 inhibition to activate p53 (Vaseva et al., 2011).

Specific structural features and conformational alterations upon interacting with p53 or inhibitors can inform rational drug design targeting MDM2 or MDMX. Structures of p53-MDM2 and p53-MDMX complexes revealed that their respective binding pockets are significantly different in depth and shape (Kussie et al., 1996; Popowicz et al., 2007). Distinctive conformational changes of MDM2 and MDMX upon inhibitor bindings were also identified by performing computer-aided analysis of molecular dynamics simulations (Chen et al., 2013; Chen et al., 2015). Taking advantages of these unique characteristics has led to the identification of p53 activator Inauhzin using computational structure-based screening (Zhang et al., 2012). Although Inauhzin was later found to activate p53 through inhibiting SIRT1 instead of MDMX or MDM2, similar approaches could lead to development of inhibitors distinguishing or combining MDM2-and MDMX-targeting activities.

The close structural and functional relationship between MDM2 and MDMX means that some molecules, originally identified as MDM2 inhibitors, were found to exert their activities through interfering with the MDM2/MDMX complex. The examples include MEL23 and its analogs, a number of MMRi (MDM2-MDMX RING domain inhibitors), and a pyrrolidone derivative that inhibits E3 ligase activity of MDM2/MDMX complex to activate p53 (Herman et al., 2011; Zhuang et al., 2012; Wu et al., 2015). Graves et al. (2012) instead discovered a couple of indolyl hydantoin compounds that restore p53-mediated apoptotic activity by promoting formation of dimeric complexes between MDM2 and MDMX to sequester them away from p53.

The aforementioned p53-derived stapled peptide, ALRN-6924, represents another approach to disrupt protein-protein interactions between p53 and both MDM2 and MDMX (Bernal et al., 2010; Brown et al., 2013). Interestingly, recent data from early clinical trials of ALRN-6924 showed superior toxicity profiles compared to other MDM2 inhibitors (Konopleva et al., 2020; Saleh et al., 2021). It is speculated that this observation might be attributed to ALRN-6924’s dual MDM2/MDMX inhibitor status, making it a milder MDM2 inhibitor in certain tissues to minimize toxicity. It suggests that ALRN-6924, or other MDM2/MDMX dual-inhibitors, have potential as chemoprotective agents when used alongside other potent yet highly toxic chemotherapeutic drugs (Carvajal et al., 2005).

As our understanding of mechanisms surrounding MDM2 and MDMX grows (comprehensively reviewed by Klein et al*.*), so will our ability to design and develop cancer therapies based on disease/tissue-specific relationships between p53, MDM2 and MDMX (see reviews by Nguyen et al. and Burgess et al. for detailed overview of therapies targeting MDM2/MDMX-p53) (Burgess et al., 2016; Nguyen et al., 2017; Klein et al., 2021).

### Justify the Value of ARF-Targeting Cancer Therapies

Development of ARF-targeting therapies has been handicapped by following misperceptions: 1) Linear relationship between ARF and p53: ARF and p53 are often thought to act in a linear pathway to inhibit tumorigenesis. With progress already made in developing p53-activating therapies and general difficulties in activating tumor suppressors, there is little need to devote much attention on ARF. 2) Emergence of CDK4/6 inhibitors: Recent development of selective CDK4/6 inhibitors resulted in, compared with previous generations of CDK inhibitors, lower toxicities, higher tumor-suppressive activities, and enhanced tumor immunogenicity (O'Leary et al., 2016; Goel et al., 2017). These drugs are considered magic bullets against *CDKN2A*-deficient cancers and have demonstrated promising results in preclinical/clinical settings. 3) Promise of cancer immunotherapy: Harnessing patients’ own immune system to treat cancer has long been believed to be the holy grail in cancer therapy. Within the last 10 years, that belief has come to fruition with numbers of modern cancer immunotherapies, including checkpoint blockade and chimeric antigen receptor (CAR) T-cell therapies among others, dominate our attention in the fight against cancer. Cancer immunotherapy, theoretically, battles cancers in the most systematic way, bypassing needs to consider any specific aberrations in tumor-associated factors, such as tumor suppressors (Waldman et al., 2020).

A compelling argument can be made, however, to counter these misperceptions and support a strong pursuit of ARF-based cancer therapies: 1) With the number of p53-independent functions of ARF identified, there is a clear need to focus on developing therapies specifically targeting ARF-mediated pathways. ARF-based therapies have potential to synergize with p53-targeting drugs to inhibit tumorigenesis through shared tumor-suppressive mechanisms like apoptosis, autophagy and ferroptosis. 2) Despite early clinical promise, intrinsic and acquired resistance to CDK4/6 inhibitors have hindered their effectiveness (Xu et al., 2021). Mechanisms underlying resistance to CDK4/6 inhibitors, such as loss of retinoblastoma (RB1) function, are being investigated to develop complementary or combination strategies. There is little known, however, about the role of ARF in both resistance and potential complement to CDK4/6 inhibitors. It is reasonable to believe that ARF-based therapies can provide synergistic effects with CDK4/6 inhibitors, especially in *CDKN2A*-deficient cancers. 3) Cancer immunotherapy has brought great promise, but also inevitably raised significant questions. Among challenges faced by the future of cancer immunotherapy, is understanding cancer-intrinsic factors regulating TME leading to immune evasion (Wellenstein and de Visser, 2018; Hegde and Chen, 2020). Recent studies found that genomic *CDKN2A* loss-of-function is associated with worse clinical outcome in patients treated with cancer immunotherapy in multiple cancer types (Adib et al., 2021; Gutiontov et al., 2021; Zhu et al., 2021). The reduced benefit of cancer immunotherapy can be attributed to altered tumor-immune microenvironment and compromised immune cell functions. With previous studies demonstrating ARF’s ability to activate innate and adaptive immune responses within cancer cells to suppress tumorigenesis *in vivo*, ARF-targeting strategies present opportunities to augment existing cancer immunotherapies (Yetil et al., 2015; Cerqueira et al., 2020).

### Development of ARF-Based Therapeutic Strategies—Therapeutic Peptides

Strategies to develop ARF-based therapies have so far been limited to gene therapy and therapeutic peptides. Adenovirus-mediated delivery of ARF had mostly been used experimentally *in vitro*, until recent studies showing its potential application using *in vivo* mouse cancer models (Saadatmandi et al., 2002; Tango et al., 2002; Cerqueira et al., 2020). This approach is expected to encounter similar obstacles faced by other gene therapies, including safety concerns and regulatory challenges, before reaching clinics (AuthorAnonymous, 2021).

In the absence of pharmaceutically proven activators, therapeutic peptides are viewed as viable alternatives to restore ARF functions. Development of therapeutic peptides in cancer therapy has seen more success and broader applications recently (Marqus et al., 2017; Xie et al., 2020). Compared to small molecules, therapeutic peptides generally have advantages of high potency, high specificity, wider range of targets and low toxicity. Recent advance and maturation of technologies for peptide synthesis, modification and delivery have helped overcome many of therapeutic peptides’ shortcomings, such as poor metabolic stability, lack of oral bioavailability and high manufacturing cost (Muttenthaler et al., 2021). As peptides of 40 or less amino acids in length are regulated as small molecules for clinical applications, therapeutic peptides are uniquely positioned to fill the gap between small molecules and biologics for unmet medical needs (Rastogi et al., 2019). Peptide-based therapies also possess intrinsic characteristics, such as their propensity to cross blood-brain barrier, to make them superior drug candidates over small molecules for certain diseases (ex. central nervous system cancers) (Zhou et al., 2021b). Interference peptides designed to disrupt protein-protein interactions, like previously mentioned ALRN-6924, are relatively easy to design to achieve high target specificity and selectivity (Sorolla et al., 2020). To directly rescue or supplement for defective or deleted genes, such as tumor suppressors like ARF, peptide mimetics containing functionally significant motifs represent a new and flexible class of cancer therapeutic drugs.

A peptide containing N-terminal portion of ARF (aa 1–20) was found to bind MDM2 and inhibit p53 ubiquitination *in vitro* (Midgley et al., 2000). This observation confirmed functional significance of the N-terminal region of ARF, and synthetic peptides containing this region showed cytotoxic activity against cancer cells (Johansson et al., 2008). Although N-terminus ARF peptides display intrinsic cell-penetrating ability, potent CPPs of ARF have been generated by addition of a poly-arginine protein transduction domain (PTD) known to promote cell permeability, stability and efficacy of therapeutic peptides (Kondo et al., 2008; Allolio et al., 2018). Poly-arginine PTD has also been used to generate ARF-CPPs containing mitochondria-targeting domain (aa 38–65) to show tumor-suppressive activities in multiple cancer types (Saito et al., 2013; Saito et al., 2016). The main challenge for future development of ARF-CPPs is to achieve an acceptable balance between anti-tumor efficacy and undesired toxicity, often seen in arginine-rich CPPs to dampen confidence for their eventual clinical applications (Li et al., 2017; Lafarga et al., 2021).

### Development of ARF-Based Therapeutic Strategies—Functional Antagonists Against ARF Deficiency

Another approach to develop ARF-based therapies is to identify points of synthetic lethality associated with ARF deficiency. This concept is behind the clinical success of Poly-(ADP-ribose)-polymerase (PARP) inhibitors in treatment of *BRCA1/2*-mutant cancers and has been used to identify therapeutic targets associated with defective tumor suppressors, including p53 (Gurpinar and Vousden, 2015; Patel et al., 2021). With recent advancements in cancer cohort datasets and experimental toolkits, functional proteogenomic analysis has been used to discover synthetic lethality driven by loss-of-function tumor suppressors (Xiao et al., 2020; Lei and Zhang, 2021). This strategy has been applied in cancers with high level of *CDKN2A* deficiency, but further analyses and functional validations will be needed to delineate synthetic lethality associated specifically with ARF deficiency, as most efforts to identify therapeutic opportunities associated with *CDKN2A* deficiency begin and end at p16-CDK4/6-RB pathway intervention (Oh et al., 2020; Cao et al., 2021; Satpathy et al., 2021). Using a similar approach, Zhu et al. found that breast cancer cells with *CDKN2A* mutations are more sensitive to a TTK/CLK2 inhibitor, CC-671. Whether this discovery can be attributed to ARF deficiency requires further investigation (Zhu et al., 2018).

Alternatively, pharmacogenomic screens based on concept of functional antagonism can be used to identify targetable vulnerabilities associated with dysfunctional tumor suppressors. Functionally defined or novel/diverse drug libraries are used in high-throughput screening to determine if the presence/absence of tumor suppressor genes in cancer cells dictates their response. This strategy could be useful to identify novel therapeutic targets or repurpose existing drugs by matching pharmacological sensitivity and genetic alterations. Bitler et al. used this approach to identify EZH2 methyltransferase as a novel target in *ARID1A*-mutated ovarian cancers, and EZH2 inhibition has since been explored as a viable therapy in other cancers with *ARID1A* mutations (Bitler et al., 2015; Alldredge and Eskander, 2017; Ferguson et al., 2021; Yamada et al., 2021). The same approach was also utilized in recent studies to identify novel therapeutic opportunities against cancers with defective RB1 tumor suppressor. Witkiewicz et al. used TNBC cells treated with CDK4/6 inhibitors and an FDA-approved drug library (1,280 compounds) to identify CHK and PLK1 inhibitors specifically antagonized by functional RB, while Gong et al. applied a limited set of drugs (36 cell-cycle inhibitors) to show that inhibition of Aurora A kinase is synthetic lethal with *RB1* mutation in a panel of diverse cancer cell lines (Witkiewicz et al., 2018; Gong et al., 2019). Interestingly, Oser et al. (2019) showed that *RB1*-deficient cancer cells are dependent on Aurora B kinase for survival by performing a synthetic lethal CRISPR/Cas9 screen in lung and breast cancer cell lines. These studies demonstrated the value of using pharmacogenomic screens to identify novel therapeutic strategies against cancers with defective tumor suppressors. It is unclear if these findings can be linked to ARF deficiency in these cancers, as ARF and p16-CDK4/6-RB function through distinctive signaling pathways. What it highlights, however, is an opportunity to fill the scientific gap by applying similar approaches with ARF-specific screens, which have not been reported in the existing literature to the best of our knowledge.

The quest to identify ARF-associated synthetic lethality could benefit from publicly curated database, such as the Biological General Repository for Interaction Datasets (BioGRID) (Oughtred et al., 2021). BioGRID compiles literature-informed data for protein/genetic/chemical interactions and CRISPR-based screens. Although no *CDKN2A*/*ARF*-specific CRISPR screens have been reported, other functional interactions revealed by the literature curation could bring interesting insight. A combinatorial CRISPR/Cas9 screen identified functional/phenotypic links between *CDKN2A* and *PTEN*, *IGF1R* and *RRM2* (Shen et al., 2017). The functional relationship between *CDKN2A* and *PTEN* has been reported, while the roles of *IGF1R* and *RRM2* might shed new light in the functions of *CDKN2A* or ARF specifically upon further studies (Carrasco et al., 2006). In another example, TRIM28 interacts with ARF to maintain chromosome integrity (Neo et al., 2015). As TRIM28 was recently shown to regulate antitumor immunity, its role in ARF-mediated immune regulation could warrant further investigation (Lin et al., 2021). With ever-growing data from multi-omics analyses being fed into databases like BioGRID, artificial intelligence-aided literature mining tool, such as CompBio (https://gtac-compbio.wustl.edu/), could facilitate our ability to extract useful information more effectively (Sapkota et al., 2021).

## Conclusion

**FIGURE 5:**
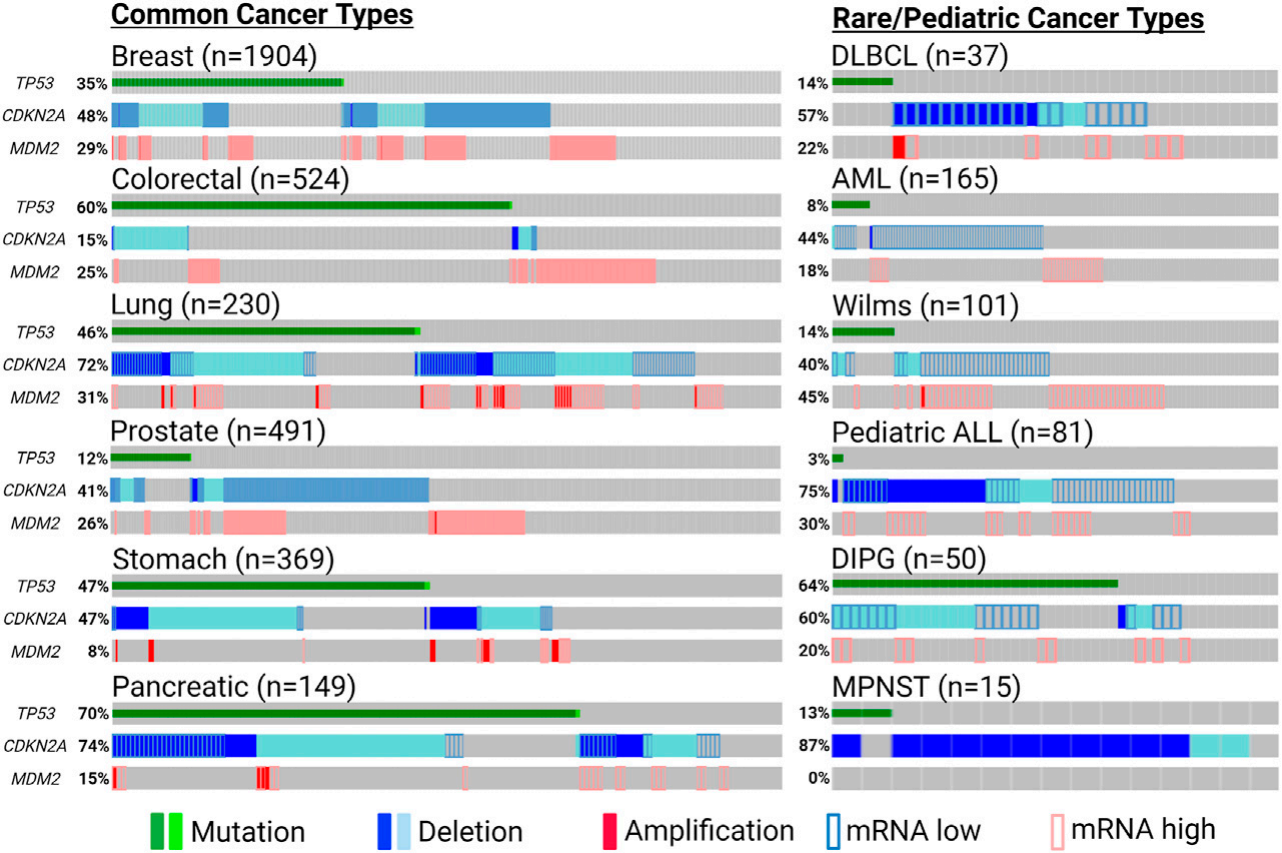
Significant genetic alterations of p53, MDM2 and ARF in cancers. Tumor-promoting genetic events of *TP53* (non-synonymous mutation), *MDM2* (amplification or induced mRNA expression) and *CDKN2A* (deletion or reduced mRNA expression) are summarized using publicly available patient data from cBioPortal (cbioportal.org) and pediatric cBioPortal (pedcbioportal.org). Percentages shown indicate accumulated fraction of patient samples with highlighted genetic alterations. The sources of the data presented are the following: Breast—METABRIC; Colorectal/DLBCL (diffuse large b cell lymphoma)/AML (acute myeloid leukemia)—TCGA PanCancer; Lung/Prostate/Stomach/Pancreatic—TCGA Firehose; DIPG (diffuse intrinsic pontine glioma)—PNOC; Wilms/pediatric ALL (acute lymphoblastic leukemia)—TARGET; MPNST (malignant peripheral nerve sheath tumor)—MSKCC. Expression of mRNA levels (except for MPNST) are shown based on expression z-scores relative to all available diploid samples (<−0.5: mRNA low; >0.5: mRNA high). Created with BioRender.com.

Countless discoveries are unquestionably to come dissecting functional interactions in and out of p53-MDM2-ARF pathway, and many questions remain regarding how to harness their relationship to maximize clinical benefits. Does co-inactivation of p53 and ARF warrant more attention as a defective tumor-suppressive entity, for which independent investigations should be conducted instead of inferring biological meanings from their loss-of-function individually? In addition to common cancer types in which p53/MDM2/ARF alterations are prevalent, could we unveil more clinical benefits in rare and pediatric malignancies targeting this axis? Pediatric cancers have much lower mutation burdens compared to adult tumors, but most of their mutations occur in a few significant cancer driver genes, such as *TP53* and *CDKN2A* (Ma et al., 2018) (Figure 5). Higher significance of these pathognomonic genetic alterations could translate to better response to targeted therapies in rare and pediatric cancers (Boyd et al., 2016; Laetsch et al., 2021). Does collective status of all three genes provide additional biomarker values in helping us tailor therapeutic strategies? For example, in cancers with functional p53 and ARF in addition to MDM2 amplification, would p53-MDM2 inhibitors sensitize tumors to ferroptosis-inducing treatments? In ARF-deficient cancers with mutant p53 and MDM2 amplification, could p53/ARF-based therapeutic peptides synergize with MDM2-targeting PROTAC? With their expanding roles identified in metabolism and TME, could p53/MDM2/ARF-based interventions synergize with metabolic and immunogenic regulations? For example, mitochondrial apoptotic priming through targeting Bcl-2/Bcl-xl was recently found to significantly enhance WT p53 activity, and might have similar effects on MDM2/ARF-targeting treatments (Sánchez-Rivera et al., 2021).

To develop genetically tailored therapeutic strategies targeting cancer vulnerabilities, open access databases play critical roles in providing up-to-date and customizable resources from cancer patients, such as The Cancer Genome Atlas (TCGA) Program (https://portal.gdc.cancer.gov/) and cBioPortal for Cancer Genomics (https://www.cbioportal.org/); from diverse mouse models of human cancer, like Mouse Models of Human Cancer Database (MMHCdb: http://tumor.informatics.jax.org/mtbwi/index.do); from patient derived xenograft (PDX) models, including PDX Finder (https://www.pdxfinder.org/) and Patient-Derived Models Repository (PDMR) Database (https://pdmr.cancer.gov/); and from cancer cell lines widely available to the research community at the Cancer Dependency Map (DepMap: https://depmap.org/portal/).

Among these resources, DepMap offers an easy-to-access, genome-scale catalog to enable research in genetic and pharmacological dependencies from hundreds of cancer cell lines. Genetic dependency scores were curated from a cohort of genome-wide RNAi and CRISPR loss-of-function screens, while pharmacological dependency data were obtained from publicly sourced drug sensitivity screens. More importantly, DepMap provides built-in analytical tools to highlight genetic co-dependencies and predict novel cancer cell vulnerabilities using multi-omics gene expression profiles (Dempster et al., 2020). For example, strong co-dependencies are identified between *TP53*-*MDM2*/*MDMX* (negatively correlated) and *MDM2*-*MDMX* (positively correlated), consistent with known biological functions. Therefore, vulnerabilities associated with the p53-MDM2-ARF pathway, such as unique targets in ARF-deficient cells, could be identified for further validations. Additionally, the consolidated database for genetic information (mRNA/protein expression, copy number, mutation, methylation … etc.) from an impressive number of cancer cell lines allows identification of cell models with the desired genetic composition to conduct relevant research.

It is worth recognizing, however, limitations with these datasets. With tissue-specific tumorigenic pathways, such as p53 signaling, data to inform cancer vulnerabilities need to be considered within the proper context and cancer indications (Schneider et al., 2017). Moreover, current genetic dependency data were mostly obtained from perturbation screens against individual genes. As the dataset grows with more input from combinatorial screens targeting multiple genes simultaneously, inaccurate/incomplete connections between genetic manipulations and their physiological significance could be better avoided (Zhao et al., 2021). The same improvement could be expected as more in-depth genetic information (ex. epitranscriptomic and epigenetic modifications) are included (Kan et al., 2021). It is also important to note that, despite its increasing applications in studying cancer vulnerability, multiple recent studies have shown that CRISPR/Cas9-based technology significantly alters p53-mediated functions and signaling pathways (Haapaniemi et al., 2018; Enache et al., 2020; Jiang et al., 2021; Sinha et al., 2021). These observations indicate that this approach might compromise the critical component of unbiasedness when identifying unique cancer vulnerabilities associated with p53-surrounding networks. Despite these caveats, these resources will continue to play important roles in developing novel cancer therapies informed by genetic signatures, including the p53-MDM2-ARF complex.

The once simple triangle between p53, MDM2 and ARF has steadily grown into a complicated network merely >20 years after it was first assembled. The only thing for certain is that our fascination with this (dys)functional complex will continue for years to come, and knowledge we gain from studying its expanded network will shape the future of cancer therapy.

## References

[B1] Abida W. M. Gu W. (2008). p53-Dependent and P53-independent Activation of Autophagy by ARF. Cancer Res. 68 (2), 352–357. PubMed PMID: 18199527; PubMed Central PMCID: PMCPMC3737745. 10.1158/0008-5472.CAN-07-2069 18199527 PMC3737745

[B2] Adib E. Nassar A. H. Akl E. W. Abou Alaiwi S. Nuzzo P. V. Mouhieddine T. H. (2021). CDKN2A Alterations and Response to Immunotherapy in Solid Tumors. Clin. Cancer Res. 27 (14), 4025–4035. PubMed PMID: 34074656. 10.1158/1078-0432.CCR-21-0575 34074656 PMC8900067

[B3] Alldredge J. K. Eskander R. N. (2017). EZH2 Inhibition in ARID1A Mutated clear Cell and Endometrioid Ovarian and Endometrioid Endometrial Cancers. Gynaecol. Oncol. Res. Pract. 4, 17. PubMed PMID: 29093822; PubMed Central PMCID: PMCPMC5663065. 10.1186/s40661-017-0052-y PMC5663065 29093822

[B4] Allolio C. Magarkar A. Jurkiewicz P. Baxová K. Javanainen M. Mason P. E. (2018). Arginine-rich Cell-Penetrating Peptides Induce Membrane Multilamellarity and Subsequently Enter via Formation of a Fusion Pore. Proc. Natl. Acad. Sci. USA 115 (47), 11923–11928. PubMed PMID: 30397112; PubMed Central PMCID: PMCPMC6255155. 10.1073/pnas.1811520115 30397112 PMC6255155

[B5] Aloni-Grinstein R. Charni-Natan M. Solomon H. Rotter V. (2018). p53 and the Viral Connection: Back into the Future ‡. Cancers (Basel) 10 (6), 1. PubMed PMID: 29866997; PubMed Central PMCID: PMCPMC6024945. 10.3390/cancers10060178 PMC6024945 29866997

[B6] Apicelli A. J. Maggi L. B. Jr. Hirbe A. C. Miceli A. P. Olanich M. E. Schulte-Winkeler C. L. (2008). A Non-tumor Suppressor Role for Basal P19 ARF in Maintaining Nucleolar Structure and Function. Mol. Cell Biol 28 (3), 1068–1080. PubMed PMID: 18070929; PubMed Central PMCID: PMCPMC2223401. 10.1128/MCB.00484-07 18070929 PMC2223401

[B7] Arena G. Cissé M. Y. Pyrdziak S. Chatre L. Riscal R. Fuentes M. (2018). Mitochondrial MDM2 Regulates Respiratory Complex I Activity Independently of P53. Mol. Cell 69 (4), 594–609. PubMed PMID: 29452639; PubMed Central PMCID: PMCPMC8215449. 10.1016/j.molcel.2018.01.023 29452639 PMC8215449

[B8] Editorial (2021). Gene Therapy Needs a Long-Term Approach. Nat. Med. 27 (4), 563. PubMed PMID: 33859432. 10.1038/s41591-021-01333-6 33859432

[B9] Ayrault O. Andrique L. Larsen C.-J. Seite P. (2004). Human Arf Tumor Suppressor Specifically Interacts with Chromatin Containing the Promoter of rRNA Genes. Oncogene 23 (49), 8097–8104. PubMed PMID: 15361825. 10.1038/sj.onc.1207968 15361825

[B10] Barak Y. Juven T. Haffner R. Oren M. (1993). mdm2 Expression Is Induced by Wild Type P53 Activity. EMBO J. 12 (2), 461–468. PubMed PMID: 8440237; PubMed Central PMCID: PMCPMC413229. 10.1002/j.1460-2075.1993.tb05678.x 8440237 PMC413229

[B11] Bernal F. Wade M. Godes M. Davis T. N. Whitehead D. G. Kung A. L. (2010). A Stapled P53 helix Overcomes HDMX-Mediated Suppression of P53. Cancer Cell 18 (5), 411–422. PubMed PMID: 21075307; PubMed Central PMCID: PMCPMC3050021. 10.1016/j.ccr.2010.10.024 21075307 PMC3050021

[B12] Bertwistle D. Sugimoto M. Sherr C. J. (2004). Physical and Functional Interactions of the Arf Tumor Suppressor Protein with nucleophosmin/B23. Mol. Cell Biol 24 (3), 985–996. PubMed PMID: 14729947; PubMed Central PMCID: PMCPMC321449. 10.1128/MCB.24.3.985-996.2004 14729947 PMC321449

[B13] Bitler B. G. Aird K. M. Garipov A. Li H. Amatangelo M. Kossenkov A. V. (2015). Synthetic Lethality by Targeting EZH2 Methyltransferase Activity in ARID1A-Mutated Cancers. Nat. Med. 21 (3), 231–238. 3799. PubMed PMID: 25686104; PubMed Central PMCID: PMCPMC4352133. 10.1038/nm10.1038/nm.3799 25686104 PMC4352133

[B14] Bond G. L. Hirshfield K. M. Kirchhoff T. Alexe G. Bond E. E. Robins H. (2006). MDM2 SNP309 Accelerates Tumor Formation in a Gender-specific and Hormone-dependent Manner. Cancer Res. 66 (10), 5104–5110. PubMed PMID: 16707433. 10.1158/0008-5472.CAN-06-0180 16707433

[B15] Bond G. L. Hu W. Bond E. E. Robins H. Lutzker S. G. Arva N. C. (2004). A Single Nucleotide Polymorphism in the MDM2 Promoter Attenuates the P53 Tumor Suppressor Pathway and Accelerates Tumor Formation in Humans. Cell 119 (5), 591–602. PubMed PMID: 15550242. 10.1016/j.cell.2004.11.022 15550242

[B16] Böttger A. Böttger V. Sparks A. Liu W.-L. Howard S. F. Lane D. P. (1997). Design of a Synthetic Mdm2-Binding Mini Protein that Activates the P53 Response *In Vivo* . Curr. Biol. 7 (11), 860–869. PubMed PMID: 9382809. 10.1016/s0960-9822(06)00374-5 9382809

[B17] Boutelle A. M. Attardi L. D. (2021). p53 and Tumor Suppression: It Takes a Network. Trends Cell Biol. 31 (4), 298–310. PubMed PMID: 33518400; PubMed Central PMCID: PMCPMC7954925. 10.1016/j.tcb.2020.12.011 33518400 PMC7954925

[B18] Bowling S. Di Gregorio A. Sancho M. Pozzi S. Aarts M. Signore M. (2018). P53 and mTOR Signalling Determine Fitness Selection through Cell Competition during Early Mouse Embryonic Development. Nat. Commun. 9 (1), 1763. PubMed PMID: 29720666; PubMed Central PMCID: PMCPMC5932021. 10.1038/s41467-018-04167-y 29720666 PMC5932021

[B19] Boyd N. Dancey J. E. Gilks C. B. Huntsman D. G. (2016). Rare Cancers: a Sea of Opportunity. Lancet Oncol. 17 (2), e52–e61. PubMed PMID: 26868354. 10.1016/S1470-2045(15)00386-1 26868354

[B20] Brady S. N. Yu Y. Maggi L. B. Jr. Weber J. D. (2004). ARF Impedes NPM/B23 Shuttling in an Mdm2-Sensitive Tumor Suppressor Pathway. Mol. Cell Biol 24 (21), 9327–9338. PubMed PMID: 15485902; PubMed Central PMCID: PMCPMC522235. 10.1128/MCB.24.21.9327-9338.2004 15485902 PMC522235

[B21] Brown C. J. Quah S. T. Jong J. Goh A. M. Chiam P. C. Khoo K. H. (2013). Stapled Peptides with Improved Potency and Specificity that Activate P53. ACS Chem. Biol. 8 (3), 506–512. PubMed PMID: 23214419. 10.1021/cb3005148 23214419

[B22] Budanov A. V. Karin M. (2008). p53 Target Genes Sestrin1 and Sestrin2 Connect Genotoxic Stress and mTOR Signaling. Cell 134 (3), 451–460. PubMed PMID: 18692468; PubMed Central PMCID: PMCPMC2758522. 10.1016/j.cell.2008.06.028 18692468 PMC2758522

[B23] Budina-Kolomets A. Hontz R. D. Pimkina J. Murphy M. E. (2013). A Conserved Domain in Exon 2 Coding for the Human and Murine ARF Tumor Suppressor Protein Is Required for Autophagy Induction. Autophagy 9 (10), 1553–1565. PubMed PMID: 23939042; PubMed Central PMCID: PMCPMC4623555. 10.4161/auto.25831 23939042 PMC4623555

[B24] Burgess A. Chia K. M. Haupt S. Thomas D. Haupt Y. Lim E. (2016). Clinical Overview of MDM2/X-Targeted Therapies. Front. Oncol. 6, 7. PubMed PMID: 26858935; PubMed Central PMCID: PMCPMC4728205. 10.3389/fonc.2016.00007 26858935 PMC4728205

[B25] Cahilly-Snyder L. Yang-Feng T. Francke U. George D. L. (1987). Molecular Analysis and Chromosomal Mapping of Amplified Genes Isolated from a Transformed Mouse 3T3 Cell Line. Somat Cell Mol Genet 13 (3), 235–244. PubMed PMID: 3474784. 10.1007/BF01535205 3474784

[B26] Cao L. Huang C. Cui Zhou D. Hu Y. Lih T. M. Savage S. R. (2021). Proteogenomic Characterization of Pancreatic Ductal Adenocarcinoma. Cell 184 (19), 5031–e26. PubMed PMID: 34534465; PubMed Central PMCID: PMCPMC8654574. 10.1016/j.cell.2021.08.023 34534465 PMC8654574

[B27] Cardozo C. M. Hainaut P. (2021). Viral Strategies for Circumventing P53: the Case of Severe Acute Respiratory Syndrome Coronavirus. Curr. Opin. Oncol. 33 (2), 149–158. PubMed PMID: 33405482; PubMed Central PMCID: PMCPMC7924916. 10.1097/CCO.0000000000000713 33405482 PMC7924916

[B28] Carlos A. R. Escandell J. M. Kotsantis P. Suwaki N. Bouwman P. Badie S. (2013). ARF Triggers Senescence in Brca2-Deficient Cells by Altering the Spectrum of P53 Transcriptional Targets. Nat. Commun. 4, 2697. PubMed PMID: 24162189. 10.1038/ncomms3697 24162189

[B29] Carrasco D. R. Fenton T. Sukhdeo K. Protopopova M. Enos M. You M. J. (2006). The PTEN and INK4A/ARF Tumor Suppressors Maintain Myelolymphoid Homeostasis and Cooperate to Constrain Histiocytic Sarcoma Development in Humans. Cancer Cell 9 (5), 379–390. PubMed PMID: 16697958. 10.1016/j.ccr.2006.03.028 16697958

[B30] Carvajal D. Tovar C. Yang H. Vu B. T. Heimbrook D. C. Vassilev L. T. (2005). Activation of P53 by MDM2 Antagonists Can Protect Proliferating Cells from Mitotic Inhibitors. Cancer Res. 65 (5), 1918–1924. PubMed PMID: 15753391. 10.1158/0008-5472.CAN-04-3576 15753391

[B31] Carvajal L. A. Neriah D. B. Senecal A. Benard L. Thiruthuvanathan V. Yatsenko T. (2018). Dual Inhibition of MDMX and MDM2 as a Therapeutic Strategy in Leukemia. Sci. Transl Med. 10, 10. PubMed PMID: 29643228; PubMed Central PMCID: PMCPMC6130841. 10.1126/scitranslmed.aao3003 PMC6130841 29643228

[B32] Cerami E. Gao J. Dogrusoz U. Gross B. E. Sumer S. O. Aksoy B. A. (2012). The cBio Cancer Genomics Portal: An Open Platform for Exploring Multidimensional Cancer Genomics Data: Figure 1. Cancer Discov. 2 (5), 401–404. PubMed PMID: 22588877; PubMed Central PMCID: PMCPMC3956037. 10.1158/2159-8290.CD-12-0095 22588877 PMC3956037

[B33] Cerqueira O. L. D. Clavijo-Salomon M. A. Cardoso E. C. Citrangulo Tortelli Junior T. Mendonça S. A. Barbuto J. A. M. (2020). Combined p14ARF and Interferon-β Gene Transfer to the Human Melanoma Cell Line SK-MEL-147 Promotes Oncolysis and Immune Activation. Front. Immunol. 11, 576658. PubMed PMID: 33193370; PubMed Central PMCID: PMCPMC7642851. 10.3389/fimmu.2020.576658 33193370 PMC7642851

[B34] Chen D. Tavana O. Chu B. Erber L. Chen Y. Baer R. (2017). NRF2 Is a Major Target of ARF in P53-independent Tumor Suppression. Mol. Cell 68 (1), 224–232. PubMed PMID: 28985506; PubMed Central PMCID: PMCPMC5683418. 10.1016/j.molcel.2017.09.009 28985506 PMC5683418

[B35] Chen J. Wang J. Zhang Q. Chen K. Zhu W. (2015). Probing Origin of Binding Difference of Inhibitors to MDM2 and MDMX by Polarizable Molecular Dynamics Simulation and QM/MM-GBSA Calculation. Sci. Rep. 5, 17421. PubMed PMID: 26616018; PubMed Central PMCID: PMCPMC4663504. 10.1038/srep17421 26616018 PMC4663504

[B36] Chen J. Wang J. Zhu W. Li G. (2013). A Computational Analysis of Binding Modes and Conformation Changes of MDM2 Induced by P53 and Inhibitor Bindings. J. Comput. Aided Mol. Des. 27 (11), 965–974. PubMed PMID: 24264557. 10.1007/s10822-013-9693-z 24264557

[B37] Chen L. Chen J. (2003). MDM2-ARF Complex Regulates P53 Sumoylation. Oncogene 22 (34), 5348–5357. PubMed PMID: 12917636. 10.1038/sj.onc.1206851 12917636

[B38] Chi S.-W. Lee S.-H. Kim D.-H. Ahn M.-J. Kim J.-S. Woo J.-Y. (2005). Structural Details on Mdm2-P53 Interaction. J. Biol. Chem. 280 (46), 38795–38802. PubMed PMID: 16159876. 10.1074/jbc.M508578200 16159876

[B39] Christensen C. Bartkova J. Mistrík M. Hall A. Lange M. K. Ralfkiær U. (2014). A Short Acidic Motif in ARF Guards against Mitochondrial Dysfunction and Melanoma Susceptibility. Nat. Commun. 5, 5348. PubMed PMID: 25370744. 10.1038/ncomms6348 25370744

[B40] Clair S. S. Giono L. Varmeh-Ziaie S. Resnick-Silverman L. Liu W.-j. Padi A. (2004). DNA Damage-Induced Downregulation of Cdc25C Is Mediated by P53 via Two Independent Mechanisms. Mol. Cell 16 (5), 725–736. PubMed PMID: 15574328. 10.1016/j.molcel.2004.11.002 15574328

[B41] Coffill C. R. Lee A. P. Siau J. W. Chee S. M. Joseph T. L. Tan Y. S. (2016). The P53-Mdm2 Interaction and the E3 Ligase Activity of Mdm2/Mdm4 Are Conserved from Lampreys to Humans. Genes Dev. 30 (3), 281–292. PubMed PMID: 26798135; PubMed Central PMCID: PMCPMC4743058. 10.1101/gad.274118.115 26798135 PMC4743058

[B42] Colombo E. Martinelli P. Zamponi R. Shing D. C. Bonetti P. Luzi L. (2006). Delocalization and Destabilization of the Arf Tumor Suppressor by the Leukemia-Associated NPM Mutant. Cancer Res. 66 (6), 3044–3050. PubMed PMID: 16540653. 10.1158/0008-5472.CAN-05-2378 16540653

[B43] Cottrell K. A. Chiou R. C. Weber J. D. (2020). Upregulation of 5′-terminal Oligopyrimidine mRNA Translation upon Loss of the ARF Tumor Suppressor. Sci. Rep. 10 (1), 22276. PubMed PMID: 33335292; PubMed Central PMCID: PMCPMC7747592. 10.1038/s41598-020-79379-8 33335292 PMC7747592

[B44] Dale B. Cheng M. Park K.-S. Kaniskan H. Ü. Xiong Y. Jin J. (2021). Advancing Targeted Protein Degradation for Cancer Therapy. Nat. Rev. Cancer 21 (10), 638–654. PubMed PMID: 34131295; PubMed Central PMCID: PMCPMC8463487. 10.1038/s41568-021-00365-x 34131295 PMC8463487

[B45] Daniele S. Costa B. Zappelli E. Da Pozzo E. Sestito S. Nesi G. (2015). Combined Inhibition of AKT/mTOR and MDM2 Enhances Glioblastoma Multiforme Cell Apoptosis and Differentiation of Cancer Stem Cells. Sci. Rep. 5, 9956. PubMed PMID: 25898313; PubMed Central PMCID: PMCPMC4404683. 10.1038/srep09956 25898313 PMC4404683

[B46] Danovi D. Meulmeester E. Pasini D. Migliorini D. Capra M. Frenk R. (2004). Amplification of Mdmx (Or Mdm4 ) Directly Contributes to Tumor Formation by Inhibiting P53 Tumor Suppressor Activity. Mol. Cell Biol 24 (13), 5835–5843. PubMed PMID: 15199139; PubMed Central PMCID: PMCPMC480894. 10.1128/MCB.24.13.5835-5843.2004 15199139 PMC480894

[B47] Datta A. Nag A. Pan W. Hay N. Gartel A. L. Colamonici O. (2004). Myc-ARF (Alternate reading Frame) Interaction Inhibits the Functions of Myc. J. Biol. Chem. 279 (35), 36698–36707. PubMed PMID: 15199070. 10.1074/jbc.M312305200 15199070

[B48] Datta A. Nag A. Raychaudhuri P. (2002). Differential Regulation of E2F1, DP1, and the E2F1/DP1 Complex by ARF. Mol. Cell Biol 22 (24), 8398–8408. PubMed PMID: 12446760; PubMed Central PMCID: PMCPMC139864. 10.1128/MCB.22.24.8398-8408.2002 12446760 PMC139864

[B49] Datta A. Sen J. Hagen J. Korgaonkar C. K. Caffrey M. Quelle D. E. (2005). ARF Directly Binds DP1: Interaction with DP1 Coincides with the G 1 Arrest Function of ARF. Mol. Cell Biol 25 (18), 8024–8036. PubMed PMID: 16135794; PubMed Central PMCID: PMCPMC1234342. 10.1128/MCB.25.18.8024-8036.2005 16135794 PMC1234342

[B50] de Oca Luna R. M. Wagner D. S. Lozano G. (1995). Rescue of Early Embryonic Lethality in Mdm2-Deficient Mice by Deletion of P53. Nature 378 (6553), 203–206. PubMed PMID: 7477326. 10.1038/378203a0 7477326

[B51] Dempster J. M. Krill-Burger J. M. McFarland J. M. Warren A. Boehm J. S. Vazquez F. (2020). Gene Expression Has More Power for Predicting <em>*in Vitro*</em> Cancer Cell Vulnerabilities than Genomics. Cold Spring Harbor, NY: bioRxiv, 959627. 10.1101/2020.02.21.959627

[B52] DeNicola G. M. Karreth F. A. Humpton T. J. Gopinathan A. Wei C. Frese K. (2011). Oncogene-induced Nrf2 Transcription Promotes ROS Detoxification and Tumorigenesis. Nature 475 (7354), 106–109. PubMed PMID: 21734707; PubMed Central PMCID: PMCPMC3404470. 10.1038/nature10189 21734707 PMC3404470

[B53] Dolezelova P. Cetkovska K. Vousden K. H. Uldrijan S. (2012). Mutational Analysis of Mdm2 C-Terminal Tail Suggests an Evolutionarily Conserved Role of its Length in Mdm2 Activity toward P53 and Indicates Structural Differences between Mdm2 Homodimers and Mdm2/MdmX Heterodimers. Cell Cycle 11 (5), 953–962. PubMed PMID: 22333590; PubMed Central PMCID: PMCPMC3323797. 10.4161/cc.11.5.19445 22333590 PMC3323797

[B54] Dolgin E. (2017). The Most Popular Genes in the Human Genome. Nature 551 (7681), 427–431. PubMed PMID: 32080600. 10.1038/d41586-017-07291-9 29168817

[B55] Dumaz N. Meek D. W. (1999). Serine15 Phosphorylation Stimulates P53 Transactivation but Does Not Directly Influence Interaction with HDM2. EMBO J. 18 (24), 7002–7010. PubMed PMID: 10601022; PubMed Central PMCID: PMCPMC1171763. 10.1093/emboj/18.24.7002 10601022 PMC1171763

[B56] Eischen C. M. Weber J. D. Roussel M. F. Sherr C. J. Cleveland J. L. (1999). Disruption of the ARF-Mdm2-P53 Tumor Suppressor Pathway in Myc-Induced Lymphomagenesis. Genes Dev. 13 (20), 2658–2669. PubMed PMID: 10541552; PubMed Central PMCID: PMCPMC317106. 10.1101/gad.13.20.2658 10541552 PMC317106

[B57] Elkholi R. Abraham-Enachescu I. Trotta A. P. Rubio-Patiño C. Mohammed J. N. Luna-Vargas M. P. A. (2019). MDM2 Integrates Cellular Respiration and Apoptotic Signaling through NDUFS1 and the Mitochondrial Network. Mol. Cell 74 (3), 452–465. PubMed PMID: 30879903; PubMed Central PMCID: PMCPMC6499641. 10.1016/j.molcel.2019.02.012 30879903 PMC6499641

[B58] Enache O. M. Rendo V. Abdusamad M. Lam D. Davison D. Pal S. (2020). Cas9 Activates the P53 Pathway and Selects for P53-Inactivating Mutations. Nat. Genet. 52 (7), 662–668. PubMed PMID: 32424350; PubMed Central PMCID: PMCPMC7343612. 10.1038/s41588-020-0623-4 32424350 PMC7343612

[B59] Eymin B. Claverie P. Salon C. Leduc C. Col E. Brambilla E. (2006). p14 ARF Activates a Tip60-dependent and P53-independent ATM/ATR/CHK Pathway in Response to Genotoxic Stress. Mol. Cell Biol 26 (11), 4339–4350. PubMed PMID: 16705183; PubMed Central PMCID: PMCPMC1489086. 10.1128/MCB.02240-05 16705183 PMC1489086

[B60] Eymin B. Karayan L. Séité P. Brambilla C. Brambilla E. Larsen C.-J. (2001). Human ARF Binds E2F1 and Inhibits its Transcriptional Activity. Oncogene 20 (9), 1033–1041. PubMed PMID: 11314038. 10.1038/sj.onc.1204220 11314038

[B61] Eymin B. Leduc C. Coll J.-L. Brambilla E. Gazzeri S. (2003). p14ARF Induces G2 Arrest and Apoptosis Independently of P53 Leading to Regression of Tumours Established in Nude Mice. Oncogene 22 (12), 1822–1835. PubMed PMID: 12660818. 10.1038/sj.onc.1206303 12660818

[B62] Fakharzadeh S. S. Trusko S. P. George D. L. (1991). Tumorigenic Potential Associated with Enhanced Expression of a Gene that Is Amplified in a Mouse Tumor Cell Line. EMBO J. 10 (6), 1565–1569. PubMed PMID: 2026149; PubMed Central PMCID: PMCPMC452821. 10.1002/j.1460-2075.1991.tb07676.x 2026149 PMC452821

[B63] Farkas M. Hashimoto H. Bi Y. Davuluri R. V. Resnick-Silverman L. Manfredi J. J. (2021). Distinct Mechanisms Control Genome Recognition by P53 at its Target Genes Linked to Different Cell Fates. Nat. Commun. 12 (1), 484. PubMed PMID: 33473123; PubMed Central PMCID: PMCPMC7817693. 10.1038/s41467-020-20783-z 33473123 PMC7817693

[B64] Fatyol K. Szalay A. A. (2001). The p14ARF Tumor Suppressor Protein Facilitates Nucleolar Sequestration of Hypoxia-Inducible Factor-1α (HIF-1α) and Inhibits HIF-1-Mediated Transcription. J. Biol. Chem. 276 (30), 28421–28429. PubMed PMID: 11382768. 10.1074/jbc.M102847200 11382768

[B65] Feng Y. C. Liu X. Y. Teng L. Ji Q. Wu Y. Li J. M. (2020). c-Myc Inactivation of P53 through the Pan-Cancer lncRNA MILIP Drives Cancer Pathogenesis. Nat. Commun. 11 (1), 4980. PubMed PMID: 33020477; PubMed Central PMCID: PMCPMC7536215. 10.1038/s41467-020-18735-8 33020477 PMC7536215

[B66] Feng Z. Zhang H. Levine A. J. Jin S. (2005). The Coordinate Regulation of the P53 and mTOR Pathways in Cells. Proc. Natl. Acad. Sci. 102 (23), 8204–8209. PubMed PMID: 15928081; PubMed Central PMCID: PMCPMC1142118. 10.1073/pnas.0502857102 15928081 PMC1142118

[B67] Ferguson J. E. Rehman H. Chandrashekar D. S. Chakravarthi B. V. S. K. Nepal S. Eich M-L. (2021). ARID1A-mutant and Deficient Bladder Cancer Is Sensitive to EZH2 Pharmacologic Inhibition. Cold Spring Harbor, NY: bioRxiv, 426383. 10.1101/2021.01.12.426383

[B68] Fischer M. (2017). Census and Evaluation of P53 Target Genes. Oncogene 36 (28), 3943–3956. PubMed PMID: 28288132; PubMed Central PMCID: PMCPMC5511239. 10.1038/onc.2016.502 28288132 PMC5511239

[B69] Fogal V. Richardson A. D. Karmali P. P. Scheffler I. E. Smith J. W. Ruoslahti E. (2010). Mitochondrial P32 Protein Is a Critical Regulator of Tumor Metabolism via Maintenance of Oxidative Phosphorylation. Mol. Cell Biol 30 (6), 1303–1318. PubMed PMID: 20100866; PubMed Central PMCID: PMCPMC2832503. 10.1128/MCB.01101-09 20100866 PMC2832503

[B70] Forys J. T. Kuzmicki C. E. Saporita A. J. Winkeler C. L. Maggi L. B. Jr. Weber J. D. (2014). ARF and P53 Coordinate Tumor Suppression of an Oncogenic IFN-β-STAT1-ISG15 Signaling Axis. Cell Rep. 7 (2), 514–526. PubMed PMID: 24726362; PubMed Central PMCID: PMCPMC4157460. 10.1016/j.celrep.2014.03.026 24726362 PMC4157460

[B71] Gao J. Aksoy B. A. Dogrusoz U. Dresdner G. Gross B. Sumer S. O. (2013). Integrative Analysis of Complex Cancer Genomics and Clinical Profiles Using the cBioPortal. Sci. Signal. 6, l1. pl1PubMed PMID: 23550210; PubMed Central PMCID: PMCPMC4160307. 10.1126/scisignal.2004088 PMC4160307 23550210

[B72] Gembarska A. Luciani F. Fedele C. Russell E. A. Dewaele M. Villar S. (2012). MDM4 Is a Key Therapeutic Target in Cutaneous Melanoma. Nat. Med. 18 (8), 1239–1247. 2863. PubMed PMID: 22820643; PubMed Central PMCID: PMCPMC3744207. 10.1038/nm10.1038/nm.2863 22820643 PMC3744207

[B73] Giono L. E. Resnick-Silverman L. Carvajal L. A. St Clair S. Manfredi J. J. (2017). Mdm2 Promotes Cdc25C Protein Degradation and Delays Cell Cycle Progression through the G2/M Phase. Oncogene 36 (49), 6762–6773. PubMed PMID: 28806397; PubMed Central PMCID: PMCPMC6002854. 10.1038/onc.2017.254 28806397 PMC6002854

[B74] Gnanapradeepan K. Leu J. I. Basu S. Barnoud T. Good M. Lee J. V. (2020). Increased mTOR Activity and Metabolic Efficiency in Mouse and Human Cells Containing the African-Centric Tumor-Predisposing P53 Variant Pro47Ser. Elife 9, 9. PubMed PMID: 33170774; PubMed Central PMCID: PMCPMC7661039. 10.7554/eLife.55994 PMC7661039 33170774

[B75] Goel S. DeCristo M. J. Watt A. C. BrinJones H. Sceneay J. Li B. B. (2017). CDK4/6 Inhibition Triggers Anti-tumour Immunity. Nature 548 (7668), 471–475. PubMed PMID: 28813415; PubMed Central PMCID: PMCPMC5570667. 10.1038/nature23465 28813415 PMC5570667

[B76] Gong X. Du J. Parsons S. H. Merzoug F. F. Webster Y. Iversen P. W. (2019). Aurora A Kinase Inhibition Is Synthetic Lethal with Loss of the RB1 Tumor Suppressor Gene. Cancer Discov. 9 (2), 248–263. PubMed PMID: 30373917. 10.1158/2159-8290.CD-18-0469 30373917

[B77] González-Arzola K. Díaz-Quintana A. Bernardo-García N. Casado-Combreras M. Elena-Real C. A. Velázquez-Cruz A. (2020). Mitochondrial Cytochrome <em>c</em> Liberates the Nucleophosmin-Sequestered ARF Tumor Suppressor in the Nucleolus. Cold Spring Harbor, NY: bioRxiv, 057075. 10.1101/2020.05.07.057075

[B78] Gotoh K. Morisaki T. Setoyama D. Sasaki K. Yagi M. Igami K. (2018). Mitochondrial p32/C1qbp Is a Critical Regulator of Dendritic Cell Metabolism and Maturation. Cell Rep. 25 (7), 1800–1815. PubMed PMID: 30428349. 10.1016/j.celrep.2018.10.057 30428349

[B79] Graves B. Thompson T. Xia M. Janson C. Lukacs C. Deo D. (2012). Activation of the P53 Pathway by Small-Molecule-Induced MDM2 and MDMX Dimerization. Proc. Natl. Acad. Sci. 109 (29), 11788–11793. PubMed PMID: 22745160; PubMed Central PMCID: PMCPMC3406834. 10.1073/pnas.1203789109 22745160 PMC3406834

[B80] Gross S. Immel U. D. Klintschar M. Bartel F. (2014). Germline Genetics of the P53 Pathway Affect Longevity in a Gender Specific Manner. Curr. Aging Sci. 7 (2), 91–100. PubMed PMID: 24654968. 10.2174/1874609807666140321150751 24654968

[B81] Gu J. Kawai H. Nie L. Kitao H. Wiederschain D. Jochemsen A. G. (2002). Mutual Dependence of MDM2 and MDMX in Their Functional Inactivation of P53. J. Biol. Chem. 277 (22), 19251–19254. PubMed PMID: 11953423. 10.1074/jbc.C200150200 11953423

[B82] Gu L. Zhang H. Liu T. Draganov A. Yi S. Wang B. (2018). Inhibition of MDM2 by a Rhein-Derived Compound AQ-101 Suppresses Cancer Development in SCID Mice. Mol. Cancer Ther. 17 (2), 497–507. PubMed PMID: 29282301; PubMed Central PMCID: PMCPMC6054458. 10.1158/1535-7163.MCT-17-0566 29282301 PMC6054458

[B83] Gu L. Zhang H. Liu T. Zhou S. Du Y. Xiong J. (2016). Discovery of Dual Inhibitors of MDM2 and XIAP for Cancer Treatment. Cancer Cell 30 (4), 623–636. PubMed PMID: 27666947; PubMed Central PMCID: PMCPMC5079537. 10.1016/j.ccell.2016.08.015 27666947 PMC5079537

[B84] Gurpinar E. Vousden K. H. (2015). Hitting Cancers' Weak Spots: Vulnerabilities Imposed by P53 Mutation. Trends Cell Biol. 25 (8), 486–495. PubMed PMID: 25960041. 10.1016/j.tcb.2015.04.001 25960041

[B85] Gutiontov S. I. Turchan W. T. Spurr L. F. Rouhani S. J. Chervin C. S. Steinhardt G. (2021). CDKN2A Loss-Of-Function Predicts Immunotherapy Resistance in Non-small Cell Lung Cancer. Sci. Rep. 11 (1), 20059. PubMed PMID: 34625620; PubMed Central PMCID: PMCPMC8501138. 10.1038/s41598-021-99524-1 34625620 PMC8501138

[B86] Haapaniemi E. Botla S. Persson J. Schmierer B. Taipale J. (2018). CRISPR-Cas9 Genome Editing Induces a P53-Mediated DNA Damage Response. Nat. Med. 24 (7), 927–930. PubMed PMID: 29892067. 10.1038/s41591-018-0049-z 29892067

[B87] Hafner A. Bulyk M. L. Jambhekar A. Lahav G. (2019). The Multiple Mechanisms that Regulate P53 Activity and Cell Fate. Nat. Rev. Mol. Cell Biol 20 (4), 199–210. PubMed PMID: 30824861. 10.1038/s41580-019-0110-x 30824861

[B88] Hafner A. Stewart-Ornstein J. Purvis J. E. Forrester W. C. Bulyk M. L. Lahav G. (2017). p53 Pulses lead to Distinct Patterns of Gene Expression albeit Similar DNA-Binding Dynamics. Nat. Struct. Mol. Biol. 24 (10), 840–847. 3452. PubMed PMID: 28825732; PubMed Central PMCID: PMCPMC5629117. 10.1038/nsmb10.1038/nsmb.3452 28825732 PMC5629117

[B89] Hamilton G. Abraham A. G. Morton J. Sampson O. Pefani D. E. Khoronenkova S. (2014). AKT Regulates NPM Dependent ARF Localization and P53mut Stability in Tumors. Oncotarget 5 (15), 6142–6167. 2178. PubMed PMID: 25071014; PubMed Central PMCID: PMCPMC4171619. 10.18632/oncotarget10.18632/oncotarget.2178 25071014 PMC4171619

[B90] Haupt Y. Maya R. Kazaz A. Oren M. (1997). Mdm2 Promotes the Rapid Degradation of P53. Nature 387 (6630), 296–299. PubMed PMID: 9153395. 10.1038/387296a0 9153395

[B91] He S. Ma J. Fang Y. Liu Y. Wu S. Dong G. (2021). Homo-PROTAC Mediated Suicide of MDM2 to Treat Non-small Cell Lung Cancer. Acta Pharmaceutica Sinica B 11 (6), 1617–1628. PubMed PMID: 34221872; PubMed Central PMCID: PMCPMC8245912. 10.1016/j.apsb.2020.11.022 34221872 PMC8245912

[B92] Hegde P. S. Chen D. S. (2020). Top 10 Challenges in Cancer Immunotherapy. Immunity 52 (1), 17–35. PubMed PMID: 31940268. 10.1016/j.immuni.2019.12.011 31940268

[B93] Hemmati P. G. Gillissen B. von Haefen C. Wendt J. Stärck L. Güner D. (2002). Adenovirus-mediated Overexpression of p14ARF Induces P53 and Bax-independent Apoptosis. Oncogene 21 (20), 3149–3161. PubMed PMID: 12082630. 10.1038/sj.onc.1205458 12082630

[B94] Herkert B. Dwertmann A. Herold S. Abed M. Naud J.-F. Finkernagel F. (2010). The Arf Tumor Suppressor Protein Inhibits Miz1 to Suppress Cell Adhesion and Induce Apoptosis. J. Cell Biol 188 (6), 905–918. PubMed PMID: 20308430; PubMed Central PMCID: PMCPMC2845071. 10.1083/jcb.200908103 20308430 PMC2845071

[B95] Herman A. G. Hayano M. Poyurovsky M. V. Shimada K. Skouta R. Prives C. (2011). Discovery of Mdm2-MdmX E3 Ligase Inhibitors Using a Cell-Based Ubiquitination Assay. Cancer Discov. 1 (4), 312–325. PubMed PMID: 22586610; PubMed Central PMCID: PMCPMC3353153. 10.1158/2159-8290.CD-11-0104 22586610 PMC3353153

[B96] Hines J. Lartigue S. Dong H. Qian Y. Crews C. M. (2019). MDM2-Recruiting PROTAC Offers Superior, Synergistic Antiproliferative Activity via Simultaneous Degradation of BRD4 and Stabilization of P53. Cancer Res. 79 (1), 251–262. PubMed PMID: 30385614; PubMed Central PMCID: PMCPMC6318015. 10.1158/0008-5472.CAN-18-2918 30385614 PMC6318015

[B97] Ho J. S. L. Ma W. Mao D. Y. L. Benchimol S. (2005). p53-Dependent Transcriptional Repression of C-Myc Is Required for G 1 Cell Cycle Arrest. Mol. Cell Biol 25 (17), 7423–7431. PubMed PMID: 16107691; PubMed Central PMCID: PMCPMC1190302. 10.1128/MCB.25.17.7423-7431.2005 16107691 PMC1190302

[B98] Honda R. Yasuda H. (1999). Association of p19ARF with Mdm2 Inhibits Ubiquitin Ligase Activity of Mdm2 for Tumor Suppressor P53. EMBO J. 18 (1), 22–27. PubMed PMID: 9878046; PubMed Central PMCID: PMCPMC1171098. 10.1093/emboj/18.1.22 9878046 PMC1171098

[B99] Hsueh K.-W. Fu S.-L. Chang C.-B. Chang Y.-L. Lin C.-H. (2013). A Novel Aurora-A-mediated Phosphorylation of P53 Inhibits its Interaction with MDM2. Biochim. Biophys. Acta (Bba) - Proteins Proteomics 1834 (2), 508–515. PubMed PMID: 23201157. 10.1016/j.bbapap.2012.11.005 23201157

[B100] Hu W. Feng Z. Ma L. Wagner J. Rice J. J. Stolovitzky G. (2007). A Single Nucleotide Polymorphism in theMDM2Gene Disrupts the Oscillation of P53 and MDM2 Levels in Cells. Cancer Res. 67 (6), 2757–2765. PubMed PMID: 17363597. 10.1158/0008-5472.CAN-06-2656 17363597

[B101] Huang L. Yan Z. Liao X. Li Y. Yang J. Wang Z.-G. (2011). The P53 Inhibitors MDM2/MDMX Complex Is Required for Control of P53 Activity *In Vivo* . Proc. Natl. Acad. Sci. 108 (29), 12001–12006. PubMed PMID: 21730163; PubMed Central PMCID: PMCPMC3141917. 10.1073/pnas.1102309108 21730163 PMC3141917

[B102] Humbey O. Pimkina J. Zilfou J. T. Jarnik M. Dominguez-Brauer C. Burgess D. J. (2008). The ARF Tumor Suppressor Can Promote the Progression of Some Tumors. Cancer Res. 68 (23), 9608–9613. PubMed PMID: 19047137; PubMed Central PMCID: PMCPMC2637809. 10.1158/0008-5472.CAN-08-2263 19047137 PMC2637809

[B103] Humpton T. J. Nomura K. Weber J. Magnussen H. M. Hock A. K. Nixon C. (2021). Differential Requirements for MDM2 E3 Activity during Embryogenesis and in Adult Mice. Genes Dev. 35 (1-2), 117–132. PubMed PMID: 33334825; PubMed Central PMCID: PMCPMC7778261. 10.1101/gad.341875.120 33334825 PMC7778261

[B104] Inoue K. Fry E. (2018). Aberrant Expression of p14ARF in Human Cancers: A New Biomarker? Tumor Microenviron 1 (2), 37–44. PubMed PMID: 30740529; PubMed Central PMCID: PMCPMC6364748. 10.4103/tme.tme10.4103/tme.tme_24_17 30740529 PMC6364748

[B105] Ishizawa J. Nakamaru K. Seki T. Tazaki K. Kojima K. Chachad D. (2018). Predictive Gene Signatures Determine Tumor Sensitivity to MDM2 Inhibition. Cancer Res. 78 (10), 2721–2731. PubMed PMID: 29490944; PubMed Central PMCID: PMCPMC5955838. 10.1158/0008-5472.CAN-17-0949 29490944 PMC5955838

[B106] Isobe Y. Okumura M. McGregor L. M. Brittain S. M. Jones M. D. Liang X. (2020). Manumycin Polyketides Act as Molecular Glues between UBR7 and P53. Nat. Chem. Biol. 16 (11), 1189–1198. PubMed PMID: 32572277; PubMed Central PMCID: PMCPMC7572527. 10.1038/s41589-020-0557-2 32572277 PMC7572527

[B107] Itahana K. Bhat K. P. Jin A. Itahana Y. Hawke D. Kobayashi R. (2003). Tumor Suppressor ARF Degrades B23, a Nucleolar Protein Involved in Ribosome Biogenesis and Cell Proliferation. Mol. Cell 12 (5), 1151–1164. PubMed PMID: 14636574. 10.1016/s1097-2765(03)00431-3 14636574

[B108] Itahana K. Zhang Y. (2008). Mitochondrial P32 Is a Critical Mediator of ARF-Induced Apoptosis. Cancer Cell 13 (6), 542–553. PubMed PMID: 18538737; PubMed Central PMCID: PMCPMC4504427. 10.1016/j.ccr.2008.04.002 18538737 PMC4504427

[B109] Ivanschitz L. Takahashi Y. Jollivet F. Ayrault O. Le Bras M. de Thé H. (2015). PML IV/ARF Interaction Enhances P53 SUMO-1 Conjugation, Activation, and Senescence. Proc. Natl. Acad. Sci. USA 112 (46), 14278–14283. PubMed PMID: 26578773; PubMed Central PMCID: PMCPMC4655504. 10.1073/pnas.1507540112 26578773 PMC4655504

[B110] Iyappan S. Wollscheid H.-P. Rojas-Fernandez A. Marquardt A. Tang H.-C. Singh R. K. (2010). Turning the RING Domain Protein MdmX into an Active Ubiquitin-Protein Ligase*. J. Biol. Chem. 285 (43), 33065–33072. PubMed PMID: 20705607; PubMed Central PMCID: PMCPMC2963378. 10.1074/jbc.M110.115113 20705607 PMC2963378

[B111] Jeay S. Gaulis S. Ferretti S. Bitter H. Ito M. Valat T. (2015). A Distinct P53 Target Gene Set Predicts for Response to the Selective P53-HDM2 Inhibitor NVP-Cgm097. Elife 4, 4. PubMed PMID: 25965177; PubMed Central PMCID: PMCPMC4468608. 10.7554/eLife.06498 PMC4468608 25965177

[B112] Jennis M. Kung C.-P. Basu S. Budina-Kolomets A. Leu J. I.-J. Khaku S. (2016). An African-specific Polymorphism in the TP53 Gene Impairs P53 Tumor Suppressor Function in a Mouse Model. Genes Dev. 30 (8), 918–930. PubMed PMID: 27034505; PubMed Central PMCID: PMCPMC4840298. 10.1101/gad.275891.115 27034505 PMC4840298

[B113] Jiang L. Ingelshed K. Shen Y. Boddul S. V. Iyer V. S. Kasza Z. (2021). CRISPR/Cas9-induced DNA Damage Enriches for Mutations in a P53-Linked Interactome: Implications for CRISPR-Based Therapies. Philadelphia: Cancer Res. PubMed PMID: 34750099. 10.1158/0008-5472.CAN-21-1692 PMC9397613 34750099

[B114] Jiang L. Kon N. Li T. Wang S.-J. Su T. Hibshoosh H. (2015). Ferroptosis as a P53-Mediated Activity during Tumour Suppression. Nature 520 (7545), 57–62. PubMed PMID: 25799988; PubMed Central PMCID: PMCPMC4455927. 10.1038/nature14344 25799988 PMC4455927

[B115] Johansson H. J. El-Andaloussi S. Holm T. Mäe M. Jänes J. Maimets T. (2008). Characterization of a Novel Cytotoxic Cell‐penetrating Peptide Derived from p14ARF Protein. Mol. Ther. 16 (1), 115–123. PubMed PMID: 28192705. 10.1038/sj.mt.6300346 17984975

[B116] Jones S. N. Roe A. E. Donehower L. A. Bradley A. (1995). Rescue of Embryonic Lethality in Mdm2-Deficient Mice by Absence of P53. Nature 378 (6553), 206–208. PubMed PMID: 7477327. 10.1038/378206a0 7477327

[B117] Jung S. H. Hwang H. J. Kang D. Park H. A. Lee H. C. Jeong D. (2019). mTOR Kinase Leads to PTEN-Loss-Induced Cellular Senescence by Phosphorylating P53. Oncogene 38 (10), 1639–1650. PubMed PMID: 30337688; PubMed Central PMCID: PMCPMC6755978. 10.1038/s41388-018-0521-8 30337688 PMC6755978

[B118] Kadosh E. Snir-Alkalay I. Venkatachalam A. May S. Lasry A. Elyada E. (2020). The Gut Microbiome Switches Mutant P53 from Tumour-Suppressive to Oncogenic. Nature 586 (7827), 133–138. PubMed PMID: 32728212; PubMed Central PMCID: PMCPMC7116712. 10.1038/s41586-020-2541-0 32728212 PMC7116712

[B119] Kamat C. D. Green D. E. Warnke L. Thorpe J. E. Ceriello A. Ihnat M. A. (2007). Mutant P53 Facilitates Pro-angiogenic, Hyperproliferative Phenotype in Response to Chronic Relative Hypoxia. Cancer Lett. 249 (2), 209–219. PubMed PMID: 16997458. 10.1016/j.canlet.2006.08.017 16997458

[B120] Kamer I. Daniel-Meshulam I. Zadok O. Bab-Dinitz E. Perry G. Feniger-Barish R. (2020). Stromal-MDM2 Promotes Lung Cancer Cell Invasion through Tumor-Host Feedback Signaling. Mol. Cancer Res. 18 (6), 926–937. PubMed PMID: 32169890. 10.1158/1541-7786.MCR-19-0395 32169890

[B121] Kamijo T. Bodner S. van de Kamp E. Randle D. H. Sherr C. J. (1999). Tumor Spectrum in ARF-Deficient Mice. Cancer Res. 59 (9), 2217–2222. PubMed PMID: 10232611. 10232611

[B122] Kamijo T. Weber J. D. Zambetti G. Zindy F. Roussel M. F. Sherr C. J. (1998). Functional and Physical Interactions of the ARF Tumor Suppressor with P53 and Mdm2. Proc. Natl. Acad. Sci. 95 (14), 8292–8297. PubMed PMID: 9653180; PubMed Central PMCID: PMCPMC20969. 10.1073/pnas.95.14.8292 9653180 PMC20969

[B123] Kamijo T. Zindy F. Roussel M. F. Quelle D. E. Downing J. R. Ashmun R. A. (1997). Tumor Suppression at the Mouse INK4a Locus Mediated by the Alternative Reading Frame Product P19 ARF. Cell 91 (5), 649–659. PubMed PMID: 9393858. 10.1016/s0092-8674(00)80452-3 9393858

[B124] Kan R. L. Chen J. Sallam T. (2021). Crosstalk between Epitranscriptomic and Epigenetic Mechanisms in Gene Regulation. Trends Genet. PubMed PMID: 34294427. 10.1016/j.tig.2021.06.014 PMC9093201 34294427

[B125] Kanovsky M. Raffo A. Drew L. Rosal R. Do T. Friedman F. K. (2001). Peptides from the Amino Terminal Mdm-2-Binding Domain of P53, Designed from Conformational Analysis, Are Selectively Cytotoxic to Transformed Cells. Proc. Natl. Acad. Sci. 98 (22), 12438–12443. PubMed PMID: 11606716; PubMed Central PMCID: PMCPMC60072. 10.1073/pnas.211280698 11606716 PMC60072

[B126] Karan G. Wang H. Chakrabarti A. Karan S. Liu Z. Xia Z. (2016). Identification of a Small Molecule that Overcomes HdmX-Mediated Suppression of P53. Mol. Cancer Ther. 15 (4), 574–582. PubMed PMID: 26883273; PubMed Central PMCID: PMCPMC4873346. 10.1158/1535-7163.MCT-15-0467 26883273 PMC4873346

[B127] Kastenhuber E. R. Lowe S. W. (2017). Putting P53 in Context. Cell 170 (6), 1062–1078. PubMed PMID: 28886379; PubMed Central PMCID: PMCPMC5743327. 10.1016/j.cell.2017.08.028 28886379 PMC5743327

[B128] Kawagishi H. Nakamura H. Maruyama M. Mizutani S. Sugimoto K. Takagi M. (2010). ARF Suppresses Tumor Angiogenesis through Translational Control of VEGFA mRNA. Cancer Res. 70 (11), 4749–4758. PubMed PMID: 20501856. 10.1158/0008-5472.CAN-10-0368 20501856

[B129] Kawai H. Lopez-Pajares V. Kim M. M. Wiederschain D. Yuan Z.-M. (2007). RING Domain-Mediated Interaction Is a Requirement for MDM2's E3 Ligase Activity. Cancer Res. 67 (13), 6026–6030. PubMed PMID: 17616658. 10.1158/0008-5472.CAN-07-1313 17616658

[B130] Klein A. M. de Queiroz R. M. Venkatesh D. Prives C. (2021). The Roles and Regulation of MDM2 and MDMX: it Is Not Just about P53. Genes Dev. 35 (9-10), 575–601. PubMed PMID: 33888565; PubMed Central PMCID: PMCPMC8091979. 10.1101/gad.347872.120 33888565 PMC8091979

[B131] Klimovich B. Mutlu S. Schneikert J. Elmshäuser S. Klimovich M. Nist A. (2019). Loss of P53 Function at Late Stages of Tumorigenesis Confers ARF-dependent Vulnerability to P53 Reactivation Therapy. Proc. Natl. Acad. Sci. USA 116 (44), 22288–22293. PubMed PMID: 31611375; PubMed Central PMCID: PMCPMC6825290. 10.1073/pnas.1910255116 31611375 PMC6825290

[B132] Knappskog S. Bjørnslett M. Myklebust L. M. Huijts P. E. A. Vreeswijk M. P. Edvardsen H. (2011). The MDM2 Promoter SNP285C/309G Haplotype Diminishes Sp1 Transcription Factor Binding and Reduces Risk for Breast and Ovarian Cancer in Caucasians. Cancer Cell 19 (2), 273–282. PubMed PMID: 21316605. 10.1016/j.ccr.2010.12.019 21316605

[B133] Kojima K. Shimanuki M. Shikami M. Samudio I. J. Ruvolo V. Corn P. (2008). The Dual PI3 kinase/mTOR Inhibitor PI-103 Prevents P53 Induction by Mdm2 Inhibition but Enhances P53-Mediated Mitochondrial Apoptosis in P53 Wild-type AML. Leukemia 22 (9), 1728–1736. PubMed PMID: 18548093. 10.1038/leu.2008.158 18548093

[B134] Kon N. Ou Y. Wang S. J. Li H. Rustgi A. K. Gu W. (2021). mTOR Inhibition Acts as an Unexpected Checkpoint in P53-Mediated Tumor Suppression. Genes Dev. 35 (1-2), 59–64. PubMed PMID: 33303641; PubMed Central PMCID: PMCPMC7778266. 10.1101/gad.340919.120 33303641 PMC7778266

[B135] Kondo E. Tanaka T. Miyake T. Ichikawa T. Hirai M. Adachi M. (2008). Potent Synergy of Dual Antitumor Peptides for Growth Suppression of Human Glioblastoma Cell Lines. Mol. Cancer Ther. 7 (6), 1461–1471. PubMed PMID: 18566217. 10.1158/1535-7163.MCT-07-2010 18566217

[B136] Konopleva M. Martinelli G. Daver N. Papayannidis C. Wei A. Higgins B. (2020). MDM2 Inhibition: an Important Step Forward in Cancer Therapy. Leukemia 34 (11), 2858–2874. PubMed PMID: 32651541. 10.1038/s41375-020-0949-z 32651541

[B137] Korgaonkar C. Hagen J. Tompkins V. Frazier A. A. Allamargot C. Quelle F. W. (2005). Nucleophosmin (B23) Targets ARF to Nucleoli and Inhibits its Function. Mol. Cell Biol 25 (4), 1258–1271. PubMed PMID: 15684379; PubMed Central PMCID: PMCPMC548001. 10.1128/MCB.25.4.1258-1271.2005 15684379 PMC548001

[B138] Korgaonkar C. Zhao L. Modestou M. Quelle D. E. (2002). ARF Function Does Not Require P53 Stabilization or Mdm2 Relocalization. Mol. Cell Biol 22 (1), 196–206. PubMed PMID: 11739734; PubMed Central PMCID: PMCPMC134207. 10.1128/MCB.22.1.196-206.2002 11739734 PMC134207

[B139] Korotchkina L. G. Leontieva O. V. Bukreeva E. I. Demidenko Z. N. Gudkov A. V. Blagosklonny M. V. (2010). The Choice between P53-Induced Senescence and Quiescence Is Determined in Part by the mTOR Pathway. Aging 2 (6), 344–352. PubMed PMID: 20606252; PubMed Central PMCID: PMCPMC2919254. 10.18632/aging.100160 20606252 PMC2919254

[B140] Koss B. Shields B. D. Taylor E. M. Storey A. J. Byrum S. D. Gies A. J. (2020). Epigenetic Control of Cdkn2a.Arf Protects Tumor-Infiltrating Lymphocytes from Metabolic Exhaustion. Cancer Res. 80 (21), 4707–4719. PubMed PMID: 33004350; PubMed Central PMCID: PMCPMC7642172. 10.1158/0008-5472.CAN-20-0524 33004350 PMC7642172

[B141] Kovi R. C. Paliwal S. Pande S. Grossman S. R. (2010). An ARF/CtBP2 Complex Regulates BH3-Only Gene Expression and P53-independent Apoptosis. Cell Death Differ 17 (3), 513–521. PubMed PMID: 19798104; PubMed Central PMCID: PMCPMC2924672. 10.1038/cdd.2009.140 19798104 PMC2924672

[B142] Kroonen J. S. Vertegaal A. C. O. (2021). Targeting SUMO Signaling to Wrestle Cancer. Trends Cancer 7 (6), 496–510. PubMed PMID: 33353838. 10.1016/j.trecan.2020.11.009 33353838

[B143] Krummel K. A. Lee C. J. Toledo F. Wahl G. M. (2005). The C-Terminal Lysines fine-tune P53 Stress Responses in a Mouse Model but Are Not Required for Stability Control or Transactivation. Proc. Natl. Acad. Sci. 102 (29), 10188–10193. PubMed PMID: 16006521; PubMed Central PMCID: PMCPMC1177381. 10.1073/pnas.0503068102 16006521 PMC1177381

[B144] Kuchenreuther M. J. Weber J. D. (2014). The ARF Tumor-Suppressor Controls Drosha Translation to Prevent Ras-Driven Transformation. Oncogene 33 (3), 300–307. PubMed PMID: 23318441; PubMed Central PMCID: PMCPMC3934099. 10.1038/onc.2012.601 23318441 PMC3934099

[B145] Kung C.-P. Cottrell K. A. Ryu S. Bramel E. R. Kladney R. D. Bao E. A. (2021). Evaluating the Therapeutic Potential of ADAR1 Inhibition for Triple-Negative Breast Cancer. Oncogene 40 (1), 189–202. PubMed PMID: 33110236; PubMed Central PMCID: PMCPMC7796950. 10.1038/s41388-020-01515-5 33110236 PMC7796950

[B146] Kung C.-P. Khaku S. Jennis M. Zhou Y. Murphy M. E. (2015). Identification of TRIML2, a Novel P53 Target, that Enhances P53 SUMOylation and Regulates the Transactivation of Proapoptotic Genes. Mol. Cancer Res. 13 (2), 250–262. PubMed PMID: 25256710; PubMed Central PMCID: PMCPMC4336799. 10.1158/1541-7786.MCR-14-0385 25256710 PMC4336799

[B147] Kung C.-P. Leu J. I.-J. Basu S. Khaku S. Anokye-Danso F. Liu Q. (2016). The P72R Polymorphism of P53 Predisposes to Obesity and Metabolic Dysfunction. Cell Rep. 14 (10), 2413–2425. PubMed PMID: 26947067; PubMed Central PMCID: PMCPMC4926645. 10.1016/j.celrep.2016.02.037 26947067 PMC4926645

[B148] Kung C.-P. Liu Q. Murphy M. E. (2017). The Codon 72 Polymorphism of P53 Influences Cell Fate Following Nutrient Deprivation. Cancer Biol. Ther. 18 (7), 484–491. PubMed PMID: 28475405; PubMed Central PMCID: PMCPMC5639853. 10.1080/15384047.2017.1323595 28475405 PMC5639853

[B149] Kung C.-P. Murphy M. E. (2016). The Role of the P53 Tumor Suppressor in Metabolism and Diabetes. J. Endocrinol. 231 (2), R61–R75. PubMed PMID: 27613337; PubMed Central PMCID: PMCPMC5148674. 10.1530/JOE-16-0324 27613337 PMC5148674

[B150] Kuo M.-L. den Besten W. Bertwistle D. Roussel M. F. Sherr C. J. (2004). N-terminal Polyubiquitination and Degradation of the Arf Tumor Suppressor. Genes Dev. 18 (15), 1862–1874. PubMed PMID: 15289458; PubMed Central PMCID: PMCPMC517406. 10.1101/gad.1213904 15289458 PMC517406

[B151] Kussie P. H. Gorina S. Marechal V. Elenbaas B. Moreau J. Levine A. J. (1996). Structure of the MDM2 Oncoprotein Bound to the P53 Tumor Suppressor Transactivation Domain. Science 274 (5289), 948–953. PubMed PMID: 8875929. 10.1126/science.274.5289.948 8875929

[B152] Laetsch T. W. DuBois S. G. Bender J. G. Macy M. E. Moreno L. (2021). Opportunities and Challenges in Drug Development for Pediatric Cancers. Cancer Discov. 11 (3), 545–559. PubMed PMID: 33277309; PubMed Central PMCID: PMCPMC7933059. 10.1158/2159-8290.CD-20-0779 33277309 PMC7933059

[B153] Lafarga V. Sirozh O. Díaz-López I. Galarreta A. Hisaoka M. Zarzuela E. (2021). Widespread Displacement of DNA- and RNA-Binding Factors Underlies Toxicity of Arginine-Rich Cell-Penetrating Peptides. EMBO J. 40 (13), e103311. PubMed PMID: 33978236; PubMed Central PMCID: PMCPMC8246256. 10.15252/embj.2019103311 33978236 PMC8246256

[B154] Lahav G. Rosenfeld N. Sigal A. Geva-Zatorsky N. Levine A. J. Elowitz M. B. (2004). Dynamics of the P53-Mdm2 Feedback Loop in Individual Cells. Nat. Genet. 36 (2), 147–150. PubMed PMID: 14730303. 10.1038/ng1293 14730303

[B155] Lai K. P. Leong W. F. Chau J. F. L. Jia D. Zeng L. Liu H. (2010). S6K1 Is a Multifaceted Regulator of Mdm2 that Connects Nutrient Status and DNA Damage Response. EMBO J. 29 (17), 2994–3006. PubMed PMID: 20657550; PubMed Central PMCID: PMCPMC2944047. 10.1038/emboj.2010.166 20657550 PMC2944047

[B156] Lambert P. F. Kashanchi F. Radonovich M. F. Shiekhattar R. Brady J. N. (1998). Phosphorylation of P53 Serine 15 Increases Interaction with CBP. J. Biol. Chem. 273 (49), 33048–33053. PubMed PMID: 9830059. 10.1074/jbc.273.49.33048 9830059

[B157] Lane D. P. Cheok C. F. Brown C. Madhumalar A. Ghadessy F. J. Verma C. (2010). Mdm2 and P53 Are Highly Conserved from Placozoans to Man. Cell Cycle 9 (3), 540–547. PubMed PMID: 20081368. 10.4161/cc.9.3.10516 20081368

[B158] Laptenko O. Shiff I. Freed-Pastor W. Zupnick A. Mattia M. Freulich E. (2015). The P53 C Terminus Controls Site-specific DNA Binding and Promotes Structural Changes within the central DNA Binding Domain. Mol. Cell 57 (6), 1034–1046. PubMed PMID: 25794615; PubMed Central PMCID: PMCPMC6221458. 10.1016/j.molcel.2015.02.015 25794615 PMC6221458

[B159] Lee C.-H. Inoki K. Karbowniczek M. Petroulakis E. Sonenberg N. Henske E. P. (2007). Constitutive mTOR Activation in TSC Mutants Sensitizes Cells to Energy Starvation and Genomic Damage via P53. EMBO J. 26 (23), 4812–4823. PubMed PMID: 17962806; PubMed Central PMCID: PMCPMC2099465. 10.1038/sj.emboj.7601900 17962806 PMC2099465

[B160] Lei J. T. Zhang B. (2021). Proteogenomics Drives Therapeutic Hypothesis Generation for Precision Oncology. Br. J. Cancer 125 (1), 1–3. PubMed PMID: 33767418; PubMed Central PMCID: PMCPMC8257710. 10.1038/s41416-021-01346-5 33767418 PMC8257710

[B161] Leslie P. L. Ke H. Zhang Y. (2015). The MDM2 RING Domain and central Acidic Domain Play Distinct Roles in MDM2 Protein Homodimerization and MDM2-MDMX Protein Heterodimerization. J. Biol. Chem. 290 (20), 12941–12950. PubMed PMID: 25809483; PubMed Central PMCID: PMCPMC4432308. 10.1074/jbc.M115.644435 25809483 PMC4432308

[B162] Lessard F. Morin F. Ivanchuk S. Langlois F. Stefanovsky V. Rutka J. (2010). The ARF Tumor Suppressor Controls Ribosome Biogenesis by Regulating the RNA Polymerase I Transcription Factor TTF-I. Mol. Cell 38 (4), 539–550. PubMed PMID: 20513429. 10.1016/j.molcel.2010.03.015 20513429

[B163] Leu J. I.-J. Dumont P. Hafey M. Murphy M. E. George D. L. (2004). Mitochondrial P53 Activates Bak and Causes Disruption of a Bak-Mcl1 Complex. Nat. Cell Biol 6 (5), 443–450. PubMed PMID: 15077116. 10.1038/ncb1123 15077116

[B164] Leu J. I.-J. Murphy M. E. George D. L. (2019). Mechanistic Basis for Impaired Ferroptosis in Cells Expressing the African-Centric S47 Variant of P53. Proc. Natl. Acad. Sci. USA 116 (17), 8390–8396. PubMed PMID: 30962386; PubMed Central PMCID: PMCPMC6486733. 10.1073/pnas.1821277116 30962386 PMC6486733

[B165] Lev Bar-Or R. Maya R. Segel L. A. Alon U. Levine A. J. Oren M. (2000). Generation of Oscillations by the P53-Mdm2 Feedback Loop: a Theoretical and Experimental Study. Proc. Natl. Acad. Sci. 97 (21), 11250–11255. PubMed PMID: 11016968; PubMed Central PMCID: PMCPMC17186. 10.1073/pnas.210171597 11016968 PMC17186

[B166] Levine A. J. (2020). p53: 800 Million Years of Evolution and 40 Years of Discovery. Nat. Rev. Cancer 20 (8), 471–480. PubMed PMID: 32404993. 10.1038/s41568-020-0262-1 32404993

[B167] Levine A. J. (2019). The many Faces of P53: Something for Everyone. J. Mol. Cell Biol 11 (7), 524–530. PubMed PMID: 30925588; PubMed Central PMCID: PMCPMC6736316. 10.1093/jmcb/mjz026 30925588 PMC6736316

[B168] Li D. Marchenko N. D. Moll U. M. (2011). SAHA Shows Preferential Cytotoxicity in Mutant P53 Cancer Cells by Destabilizing Mutant P53 through Inhibition of the HDAC6-Hsp90 Chaperone axis. Cell Death Differ 18 (12), 1904–1913. PubMed PMID: 21637290; PubMed Central PMCID: PMCPMC3170683. 10.1038/cdd.2011.71 21637290 PMC3170683

[B169] Li D. Marchenko N. D. Schulz R. Fischer V. Velasco-Hernandez T. Talos F. (2011). Functional Inactivation of Endogenous MDM2 and CHIP by HSP90 Causes Aberrant Stabilization of Mutant P53 in Human Cancer Cells. Mol. Cancer Res. 9 (5), 577–588. PubMed PMID: 21478269; PubMed Central PMCID: PMCPMC3097033. 10.1158/1541-7786.MCR-10-0534 21478269 PMC3097033

[B170] Li M. Luo J. Brooks C. L. Gu W. (2002). Acetylation of P53 Inhibits its Ubiquitination by Mdm2. J. Biol. Chem. 277 (52), 50607–50611. PubMed PMID: 12421820. 10.1074/jbc.C200578200 12421820

[B171] Li Q. Xu M. Cui Y. Huang C. Sun M. (2017). Arginine-rich Membrane-Permeable Peptides Are Seriously Toxic. Pharmacol. Res. Perspect. 5, 5. PubMed PMID: 28971613; PubMed Central PMCID: PMCPMC5625148. 10.1002/prp2.334 PMC5625148 28971613

[B172] Li Y. Yang J. Aguilar A. McEachern D. Przybranowski S. Liu L. (2019). Discovery of MD-224 as a First-In-Class, Highly Potent, and Efficacious Proteolysis Targeting Chimera Murine Double Minute 2 Degrader Capable of Achieving Complete and Durable Tumor Regression. J. Med. Chem. 62 (2), 448–466. PubMed PMID: 30525597; PubMed Central PMCID: PMCPMC6545112. 10.1021/acs.jmedchem.8b00909 30525597 PMC6545112

[B173] Lin J. Guo D. Liu H. Zhou W. Wang C. Müller I. (2021). The SETDB1-TRIM28 Complex Suppresses Antitumor Immunity. Cancer Immunol. Res. 9 (12), 1413–1424. PubMed PMID: 34848497; PubMed Central PMCID: PMCPMC8647838. 10.1158/2326-6066.CIR-21-0754 34848497 PMC8647838

[B174] Linares L. K. Hengstermann A. Ciechanover A. Muller S. Scheffner M. (2003). HdmX Stimulates Hdm2-Mediated Ubiquitination and Degradation of P53. Proc. Natl. Acad. Sci. 100 (21), 12009–12014. PubMed PMID: 14507994; PubMed Central PMCID: PMCPMC218704. 10.1073/pnas.2030930100 14507994 PMC218704

[B175] Lindström M. S. Jin A. Deisenroth C. White Wolf G. Zhang Y. (2007). Cancer-associated Mutations in the MDM2 Zinc finger Domain Disrupt Ribosomal Protein Interaction and Attenuate MDM2-Induced P53 Degradation. Mol. Cell Biol 27 (3), 1056–1068. PubMed PMID: 17116689; PubMed Central PMCID: PMCPMC1800693. 10.1128/MCB.01307-06 17116689 PMC1800693

[B176] Ling X. Xu C. Fan C. Zhong K. Li F. Wang X. (2014). FL118 Induces P53-dependent Senescence in Colorectal Cancer Cells by Promoting Degradation of MdmX. Cancer Res. 74 (24), 7487–7497. PubMed PMID: 25512388; PubMed Central PMCID: PMCPMC4448973. 10.1158/0008-5472.CAN-14-0683 25512388 PMC4448973

[B177] Liu D. S. Duong C. P. Haupt S. Montgomery K. G. House C. M. Azar W. J. (2017). Inhibiting the System xC−/glutathione axis Selectively Targets Cancers with Mutant-P53 Accumulation. Nat. Commun. 8, 14844. PubMed PMID: 28348409; PubMed Central PMCID: PMCPMC5379068. 10.1038/ncomms14844 28348409 PMC5379068

[B178] Liu S. Kim T.-H. Franklin D. A. Zhang Y. (2017). Protection against High-Fat-Diet-Induced Obesity in MDM2 C305F Mice Due to Reduced P53 Activity and Enhanced Energy Expenditure. Cell Rep. 18 (4), 1005–1018. PubMed PMID: 28122227; PubMed Central PMCID: PMCPMC5560502. 10.1016/j.celrep.2016.12.086 28122227 PMC5560502

[B179] Liu Y. He Y. Jin A. Tikunov A. P. Zhou L. Tollini L. A. (2014). Ribosomal Protein-Mdm2-P53 Pathway Coordinates Nutrient Stress with Lipid Metabolism by Regulating MCD and Promoting Fatty Acid Oxidation. Proc. Natl. Acad. Sci. 111 (23), E2414–E2422. PubMed PMID: 24872453; PubMed Central PMCID: PMCPMC4060669. 10.1073/pnas.1315605111 24872453 PMC4060669

[B180] Llanos S. Clark P. A. Rowe J. Peters G. (2001). Stabilization of P53 by p14ARF without Relocation of MDM2 to the Nucleolus. Nat. Cell Biol 3 (5), 445–452. PubMed PMID: 11331871. 10.1038/35074506 11331871

[B181] Lohrum M. A. E. Ashcroft M. Kubbutat M. H. G. Vousden K. H. (2000). Identification of a Cryptic Nucleolar-Localization Signal in MDM2. Nat. Cell Biol 2 (3), 179–181. PubMed PMID: 10707090. 10.1038/35004057 10707090

[B182] Lu J. Chen L. Song Z. Das M. Chen J. (2021). Hypothermia Effectively Treats Tumors with Temperature-Sensitive P53 Mutations. Cancer Res. 81 (14), 3905–3915. PubMed PMID: 33687951; PubMed Central PMCID: PMCPMC8286308. 10.1158/0008-5472.CAN-21-0033 33687951 PMC8286308

[B183] Lu W. Xie Y. Ma Y. Matusik R. J. Chen Z. (2013). ARF Represses Androgen Receptor Transactivation in Prostate Cancer. Mol. Endocrinol. 27 (4), 635–648. PubMed PMID: 23449888; PubMed Central PMCID: PMCPMC3607697. 10.1210/me.2012-1294 23449888 PMC3607697

[B184] Ma L. Wagner J. Rice J. J. Hu W. Levine A. J. Stolovitzky G. A. (2005). A Plausible Model for the Digital Response of P53 to DNA Damage. Proc. Natl. Acad. Sci. 102 (40), 14266–14271. PubMed PMID: 16186499; PubMed Central PMCID: PMCPMC1242279. 10.1073/pnas.0501352102 16186499 PMC1242279

[B185] Ma X. Liu Y. Liu Y. Alexandrov L. B. Edmonson M. N. Gawad C. (2018). Pan-cancer Genome and Transcriptome Analyses of 1,699 Paediatric Leukaemias and Solid Tumours. Nature 555 (7696), 371–376. PubMed PMID: 29489755; PubMed Central PMCID: PMCPMC5854542. 10.1038/nature25795 29489755 PMC5854542

[B186] Ma Y. Yuan R. Meng Q. Goldberg I. D. Rosen E. M. Fan S. (2000). P53-independent Down-Regulation of Mdm2 in Human Cancer Cells Treated with Adriamycin. Mol. Cell Biol. Res. Commun. 3 (2), 122–128. PubMed PMID: 10775510. 10.1006/mcbr.2000.0201 10775510

[B187] Macias E. Jin A. Deisenroth C. Bhat K. Mao H. Lindström M. S. (2010). An ARF-independent C-MYC-Activated Tumor Suppression Pathway Mediated by Ribosomal Protein-Mdm2 Interaction. Cancer Cell 18 (3), 231–243. PubMed PMID: 20832751; PubMed Central PMCID: PMCPMC4400806. 10.1016/j.ccr.2010.08.007 20832751 PMC4400806

[B188] Maddocks O. D. K. Berkers C. R. Mason S. M. Zheng L. Blyth K. Gottlieb E. (2013). Serine Starvation Induces Stress and P53-dependent Metabolic Remodelling in Cancer Cells. Nature 493 (7433), 542–546. PubMed PMID: 23242140; PubMed Central PMCID: PMCPMC6485472. 10.1038/nature11743 23242140 PMC6485472

[B189] Maggi L. B. Jr. Kuchenruether M. Dadey D. Y. A. Schwope R. M. Grisendi S. Townsend R. R. (2008). Nucleophosmin Serves as a Rate-Limiting Nuclear export Chaperone for the Mammalian Ribosome. Mol. Cell Biol 28 (23), 7050–7065. PubMed PMID: 18809582; PubMed Central PMCID: PMCPMC2593371. 10.1128/MCB.01548-07 18809582 PMC2593371

[B190] Maggi L. B. Jr. Winkeler C. L. Miceli A. P. Apicelli A. J. Brady S. N. Kuchenreuther M. J. (2014). ARF Tumor Suppression in the Nucleolus. Biochim. Biophys. Acta (Bba) - Mol. Basis Dis. 1842 (6), 831–839. PubMed PMID: 24525025. 10.1016/j.bbadis.2014.01.016 24525025

[B191] Maor-Nof M. Shipony Z. Lopez-Gonzalez R. Nakayama L. Zhang Y.-J. Couthouis J. (2021). p53 Is a central Regulator Driving Neurodegeneration Caused by C9orf72 Poly(PR). Cell 184 (3), 689–708. e20PubMed PMID: 33482083; PubMed Central PMCID: PMCPMC7886018. 10.1016/j.cell.2020.12.025 33482083 PMC7886018

[B192] Marqus S. Pirogova E. Piva T. J. (2017). Evaluation of the Use of Therapeutic Peptides for Cancer Treatment. J. Biomed. Sci. 24 (1), 21. PubMed PMID: 28320393; PubMed Central PMCID: PMCPMC5359827. 10.1186/s12929-017-0328-x 28320393 PMC5359827

[B193] Martelli F. Hamilton T. Silver D. P. Sharpless N. E. Bardeesy N. Rokas M. (2001). p19ARF Targets Certain E2F Species for Degradation. Proc. Natl. Acad. Sci. 98 (8), 4455–4460. PubMed PMID: 11274364; PubMed Central PMCID: PMCPMC31856. 10.1073/pnas.081061398 11274364 PMC31856

[B194] Matoba S. Kang J.-G. Patino W. D. Wragg A. Boehm M. Gavrilova O. (2006). p53 Regulates Mitochondrial Respiration. Science 312 (5780), 1650–1653. PubMed PMID: 16728594. 10.1126/science.1126863 16728594

[B195] Mayo L. D. Donner D. B. (2001). A Phosphatidylinositol 3-kinase/Akt Pathway Promotes Translocation of Mdm2 from the Cytoplasm to the Nucleus. Proc. Natl. Acad. Sci. 98 (20), 11598–11603. PubMed PMID: 11504915; PubMed Central PMCID: PMCPMC58775. 10.1073/pnas.181181198 11504915 PMC58775

[B196] Men H. Cai H. Cheng Q. Zhou W. Wang X. Huang S. (2021). The Regulatory Roles of P53 in Cardiovascular Health and Disease. Cell. Mol. Life Sci. 78 (5), 2001–2018. PubMed PMID: 33179140. 10.1007/s00018-020-03694-6 33179140 PMC11073000

[B197] Meng X. Carlson N. R. Dong J. Zhang Y. (2015). Oncogenic C-Myc-Induced Lymphomagenesis Is Inhibited Non-redundantly by the p19Arf-Mdm2-P53 and RP-Mdm2-P53 Pathways. Oncogene 34 (46), 5709–5717. PubMed PMID: 25823025; PubMed Central PMCID: PMCPMC4844013. 10.1038/onc.2015.39 25823025 PMC4844013

[B198] Miceli A. P. Saporita A. J. Weber J. D. (2012). Hypergrowth mTORC1 Signals Translationally Activate the ARF Tumor Suppressor Checkpoint. Mol. Cell Biol 32 (2), 348–364. PubMed PMID: 22064482; PubMed Central PMCID: PMCPMC3255786. 10.1128/MCB.06030-11 22064482 PMC3255786

[B199] Midgley C. A. Desterro J. M. Saville M. K. Howard S. Sparks A. Hay R. T. (2000). An N-Terminal p14ARF Peptide Blocks Mdm2-dependent Ubiquitination *In Vitro* and Can Activate P53 *In Vivo* . Oncogene 19 (19), 2312–2323. PubMed PMID: 10822382. 10.1038/sj.onc.1203593 10822382

[B200] Midgley C. A. Lane D. P. (1997). p53 Protein Stability in Tumour Cells Is Not Determined by Mutation but Is Dependent on Mdm2 Binding. Oncogene 15 (10), 1179–1189. PubMed PMID: 9294611. 10.1038/sj.onc.1201459 9294611

[B201] Mo P. Wang H. Lu H. Boyd D. D. Yan C. (2010). MDM2 Mediates Ubiquitination and Degradation of Activating Transcription Factor 3. J. Biol. Chem. 285 (35), 26908–26915. PubMed PMID: 20592017; PubMed Central PMCID: PMCPMC2930690. 10.1074/jbc.M110.132597 20592017 PMC2930690

[B202] Momand J. Villegas A. Belyi V. A. (2011). The Evolution of MDM2 Family Genes. Gene 486 (1-2), 23–30. PubMed PMID: 21762762; PubMed Central PMCID: PMCPMC3162079. 10.1016/j.gene.2011.06.030 21762762 PMC3162079

[B203] Monasor A. Murga M. Lopez-Contreras A. Navas C. Gomez G. Pisano D. G. (2013). INK4a/ARF Limits the Expansion of Cells Suffering from Replication Stress. Cell Cycle 12 (12), 1948–1954. PubMed PMID: 23676215; PubMed Central PMCID: PMCPMC3712892. 10.4161/cc.25017 23676215 PMC3712892

[B204] Moulin S. Llanos S. Kim S.-H. Peters G. (2008). Binding to Nucleophosmin Determines the Localization of Human and Chicken ARF but Not its Impact on P53. Oncogene 27 (17), 2382–2389. PubMed PMID: 17968318. 10.1038/sj.onc.1210887 17968318

[B205] Müer A. Overkamp T. Gillissen B. Richter A. Pretzsch T. Milojkovic A. (2012). p14ARF-induced Apoptosis in P53 Protein-Deficient Cells Is Mediated by BH3-Only Protein-independent Derepression of Bak Protein through Down-Regulation of Mcl-1 and Bcl-xL Proteins. J. Biol. Chem. 287 (21), 17343–17352. PubMed PMID: 22354970; PubMed Central PMCID: PMCPMC3366797. 10.1074/jbc.M111.314898 22354970 PMC3366797

[B206] Mullard A. (2020). p53 Programmes Plough on. Nat. Rev. Drug Discov. 19 (8), 497–500. PubMed PMID: 32665592. 10.1038/d41573-020-00130-z 32665592

[B207] Muniz V. P. Barnes J. M. Paliwal S. Zhang X. Tang X. Chen S. (2011). The ARF Tumor Suppressor Inhibits Tumor Cell Colonization Independent of P53 in a Novel Mouse Model of Pancreatic Ductal Adenocarcinoma Metastasis. Mol. Cancer Res. 9 (7), 867–877. PubMed PMID: 21636682; PubMed Central PMCID: PMCPMC3140613. 10.1158/1541-7786.MCR-10-0475 21636682 PMC3140613

[B208] Murphy M. E. Leu J. I. George D. L. (2004). p53 Moves to Mitochondria: a Turn on the Path to Apoptosis. Cell Cycle 3 (7), 836–839. PubMed PMID: 15190209. 10.4161/cc.3.7.956 15190209

[B209] Muttenthaler M. King G. F. Adams D. J. Alewood P. F. (2021). Trends in Peptide Drug Discovery. Nat. Rev. Drug Discov. 20 (4), 309–325. PubMed PMID: 33536635. 10.1038/s41573-020-00135-8 33536635

[B210] Nakamura S. Roth J. A. Mukhopadhyay T. (2000). Multiple Lysine Mutations in the C-Terminal Domain of P53 Interfere with MDM2-dependent Protein Degradation and Ubiquitination. Mol. Cell Biol 20 (24), 9391–9398. PubMed PMID: 11094089; PubMed Central PMCID: PMCPMC102195. 10.1128/MCB.20.24.9391-9398.2000 11094089 PMC102195

[B211] Neo S. H. Itahana Y. Alagu J. Kitagawa M. Guo A. K. Lee S. H. (2015). TRIM28 Is an E3 Ligase for ARF-Mediated NPM1/B23 SUMOylation that Represses Centrosome Amplification. Mol. Cel. Biol. 35 (16), 2851–2863. PubMed PMID: 26055329; PubMed Central PMCID: PMCPMC4508312. 10.1128/MCB.01064-14 PMC4508312 26055329

[B212] Nguyen D. Liao W. Zeng S. X. Lu H. (2017). Reviving the Guardian of the Genome: Small Molecule Activators of P53. Pharmacol. Ther. 178, 92–108. PubMed PMID: 28351719; PubMed Central PMCID: PMCPMC5601031. 10.1016/j.pharmthera.2017.03.013 28351719 PMC5601031

[B213] Nieminen A. I. Eskelinen V. M. Haikala H. M. Tervonen T. A. Yan Y. Partanen J. I. (2013). Myc-induced AMPK-Phospho P53 Pathway Activates Bak to Sensitize Mitochondrial Apoptosis. Proc. Natl. Acad. Sci. 110 (20), E1839–E1848. PubMed PMID: 23589839; PubMed Central PMCID: PMCPMC3657814. 10.1073/pnas.1208530110 23589839 PMC3657814

[B214] O'Leary B. Finn R. S. Turner N. C. (2016). Treating Cancer with Selective CDK4/6 Inhibitors. Nat. Rev. Clin. Oncol. 13 (7), 417–430. PubMed PMID: 27030077. 10.1038/nrclinonc.2016.26 27030077

[B215] Oh S. Yeom J. Cho H. J. Kim J.-H. Yoon S.-J. Kim H. (2020). Integrated Pharmaco-Proteogenomics Defines Two Subgroups in Isocitrate Dehydrogenase Wild-type Glioblastoma with Prognostic and Therapeutic Opportunities. Nat. Commun. 11 (1), 3288. PubMed PMID: 32620753; PubMed Central PMCID: PMCPMC7335111. 10.1038/s41467-020-17139-y 32620753 PMC7335111

[B216] Oliner J. D. Pietenpol J. A. Thiagalingam S. Gyuris J. Kinzler K. W. Vogelstein B. (1993). Oncoprotein MDM2 Conceals the Activation Domain of Tumour Suppressor P53. Nature 362 (6423), 857–860. PubMed PMID: 8479525. 10.1038/362857a0 8479525

[B217] Oser M. G. Fonseca R. Chakraborty A. A. Brough R. Spektor A. Jennings R. B. (2019). Cells Lacking the RB1 Tumor Suppressor Gene Are Hyperdependent on Aurora B Kinase for Survival. Cancer Discov. 9 (2), 230–247. PubMed PMID: 30373918; PubMed Central PMCID: PMCPMC6368871. 10.1158/2159-8290.CD-18-0389 30373918 PMC6368871

[B218] Oughtred R. Rust J. Chang C. Breitkreutz B. J. Stark C. Willems A. (2021). TheBioGRIDdatabase: A Comprehensive Biomedical Resource of Curated Protein, Genetic, and Chemical Interactions. Protein Sci. 30 (1), 187–200. 3978. PubMed PMID: 33070389; PubMed Central PMCID: PMCPMC7737760. 10.1002/pro10.1002/pro.3978 33070389 PMC7737760

[B219] Pairawan S. Zhao M. Yuca E. Annis A. Evans K. Sutton D. (2021). First in Class Dual MDM2/MDMX Inhibitor ALRN-6924 Enhances Antitumor Efficacy of Chemotherapy in TP53 Wild-type Hormone Receptor-Positive Breast Cancer Models. Breast Cancer Res. 23 (1), 29. PubMed PMID: 33663585; PubMed Central PMCID: PMCPMC7934277. 10.1186/s13058-021-01406-x 33663585 PMC7934277

[B220] Paliwal S. Pande S. Kovi R. C. Sharpless N. E. Bardeesy N. Grossman S. R. (2006). Targeting of C-Terminal Binding Protein (CtBP) by ARF Results in P53-independent Apoptosis. Mol. Cell Biol 26 (6), 2360–2372. PubMed PMID: 16508011; PubMed Central PMCID: PMCPMC1430274. 10.1128/MCB.26.6.2360-2372.2006 16508011 PMC1430274

[B221] Parant J. Chavez-Reyes A. Little N. A. Yan W. Reinke V. Jochemsen A. G. (2001). Rescue of Embryonic Lethality in Mdm4-Null Mice by Loss of Trp53 Suggests a Nonoverlapping Pathway with MDM2 to Regulate P53. Nat. Genet. 29 (1), 92–95. PubMed PMID: 11528400. 10.1038/ng714 11528400

[B222] Patel P. S. Algouneh A. Hakem R. (2021). Exploiting Synthetic Lethality to Target BRCA1/2-Deficient Tumors: where We Stand. Oncogene 40 (17), 3001–3014. PubMed PMID: 33716297. 10.1038/s41388-021-01744-2 33716297

[B223] Peng Y. Chen L. Li C. Lu W. Chen J. (2001). Inhibition of MDM2 by Hsp90 Contributes to Mutant P53 Stabilization. J. Biol. Chem. 276 (44), 40583–40590. PubMed PMID: 11507088. 10.1074/jbc.M102817200 11507088

[B224] Phelps M. Darley M. Primrose J. N. Blaydes J. P. (2003). p53-independent Activation of the Hdm2-P2 Promoter through Multiple Transcription Factor Response Elements Results in Elevated Hdm2 Expression in Estrogen Receptor Alpha-Positive Breast Cancer Cells. Cancer Res. 63 (10), 2616–2623. PubMed PMID: 12750288. 12750288

[B225] Phesse T. J. Myant K. B. Cole A. M. Ridgway R. A. Pearson H. Muncan V. (2014). Endogenous C-Myc Is Essential for P53-Induced Apoptosis in Response to DNA Damage *In Vivo* . Cell Death Differ 21 (6), 956–966. PubMed PMID: 24583641; PubMed Central PMCID: PMCPMC4013513. 10.1038/cdd.2014.15 24583641 PMC4013513

[B226] Pimkina J. Humbey O. Zilfou J. T. Jarnik M. Murphy M. E. (2009). ARF Induces Autophagy by Virtue of Interaction with Bcl-Xl. J. Biol. Chem. 284 (5), 2803–2810. PubMed PMID: 19049976; PubMed Central PMCID: PMCPMC2631963. 10.1074/jbc.M804705200 19049976 PMC2631963

[B227] Pimkina J. Murphy M. E. (2011). Interaction of the ARF Tumor Suppressor with Cytosolic HSP70 Contributes to its Autophagy Function. Cancer Biol. Ther. 12 (6), 503–509. PubMed PMID: 21738007; PubMed Central PMCID: PMCPMC3218591. 10.4161/cbt.12.6.15976 21738007 PMC3218591

[B228] Pomerantz J. Schreiber-Agus N. Liégeois N. J. Silverman A. Alland L. Chin L. (1998). The Ink4a Tumor Suppressor Gene Product, p19Arf, Interacts with MDM2 and Neutralizes MDM2's Inhibition of P53. Cell 92 (6), 713–723. PubMed PMID: 9529248. 10.1016/s0092-8674(00)81400-2 9529248

[B229] Popowicz G. M. Czarna A. Rothweiler U. Szwagierczak A. Krajewski M. Weber L. (2007). Molecular Basis for the Inhibition of P53 by Mdmx. Cell Cycle 6 (19), 2386–2392. PubMed PMID: 17938582. 10.4161/cc.6.19.4740 17938582

[B230] Post S. M. Quintás-Cardama A. Pant V. Iwakuma T. Hamir A. Jackson J. G. (2010). A High-Frequency Regulatory Polymorphism in the P53 Pathway Accelerates Tumor Development. Cancer Cell 18 (3), 220–230. PubMed PMID: 20832750; PubMed Central PMCID: PMCPMC2944041. 10.1016/j.ccr.2010.07.010 20832750 PMC2944041

[B231] Poyurovsky M. V. Katz C. Laptenko O. Beckerman R. Lokshin M. Ahn J. (2010). The C Terminus of P53 Binds the N-Terminal Domain of MDM2. Nat. Struct. Mol. Biol. 17 (8), 982–989. 1872. PubMed PMID: 20639885; PubMed Central PMCID: PMCPMC2922928. 10.1038/nsmb10.1038/nsmb.1872 20639885 PMC2922928

[B232] Purvis J. E. Karhohs K. W. Mock C. Batchelor E. Loewer A. Lahav G. (2012). p53 Dynamics Control Cell Fate. Science 336 (6087), 1440–1444. PubMed PMID: 22700930; PubMed Central PMCID: PMCPMC4162876. 10.1126/science.1218351 22700930 PMC4162876

[B233] Qi Y. Gregory M. A. Li Z. Brousal J. P. West K. Hann S. R. (2004). p19ARF Directly and Differentially Controls the Functions of C-Myc Independently of P53. Nature 431 (7009), 712–717. PubMed PMID: 15361884. 10.1038/nature02958 15361884

[B234] Ramos Y. F. Stad R. Attema J. Peltenburg L. T. van der Eb A. J. Jochemsen A. G. (2001). Aberrant Expression of HDMX Proteins in Tumor Cells Correlates with Wild-type P53. Cancer Res. 61 (5), 1839–1842. PubMed PMID: 11280734. 11280734

[B235] Rastogi S. Shukla S. Kalaivani M. Singh G. N. (2019). Peptide-based Therapeutics: Quality Specifications, Regulatory Considerations, and Prospects. Drug Discov. Today 24 (1), 148–162. PubMed PMID: 30296551. 10.1016/j.drudis.2018.10.002 30296551

[B236] Ravi R. Mookerjee B. Bhujwalla Z. M. Sutter C. H. Artemov D. Zeng Q. (2000). Regulation of Tumor Angiogenesis by P53-Induced Degradation of Hypoxia-Inducible Factor 1α. Genes Dev. 14 (1), 34–44. PubMed PMID: 10640274; PubMed Central PMCID: PMCPMC316350. 10.1101/gad.14.1.34 10640274 PMC316350

[B237] Reed D. Shen Y. Shelat A. A. Arnold L. A. Ferreira A. M. Zhu F. (2010). Identification and Characterization of the First Small Molecule Inhibitor of MDMX. J. Biol. Chem. 285 (14), 10786–10796. PubMed PMID: 20080970; PubMed Central PMCID: PMCPMC2856285. 10.1074/jbc.M109.056747 20080970 PMC2856285

[B238] Reef S. Shifman O. Oren M. Kimchi A. (2007). The Autophagic Inducer smARF Interacts with and Is Stabilized by the Mitochondrial P32 Protein. Oncogene 26 (46), 6677–6683. PubMed PMID: 17486078. 10.1038/sj.onc.1210485 17486078

[B239] Reef S. Zalckvar E. Shifman O. Bialik S. Sabanay H. Oren M. (2006). A Short Mitochondrial Form of p19ARF Induces Autophagy and Caspase-independent Cell Death. Mol. Cell 22 (4), 463–475. PubMed PMID: 16713577. 10.1016/j.molcel.2006.04.014 16713577

[B240] Repenning A. Happel D. Bouchard C. Meixner M. Verel-Yilmaz Y. Raifer H. (2021). PRMT1 Promotes the Tumor Suppressor Function of p14ARF and Is Indicative for Pancreatic Cancer Prognosis. EMBO J. 40 (13), e106777. PubMed PMID: 33999432; PubMed Central PMCID: PMCPMC8246066. 10.15252/embj.2020106777 33999432 PMC8246066

[B241] Ringshausen I. O'Shea C. C. Finch A. J. Swigart L. B. Evan G. I. (2006). Mdm2 Is Critically and Continuously Required to Suppress Lethal P53 Activity *In Vivo* . Cancer Cell 10 (6), 501–514. PubMed PMID: 17157790. 10.1016/j.ccr.2006.10.010 17157790

[B242] Riscal R. Schrepfer E. Arena G. Cissé M. Y. Bellvert F. Heuillet M. (2016). Chromatin-Bound MDM2 Regulates Serine Metabolism and Redox Homeostasis Independently of P53. Mol. Cell 62 (6), 890–902. PubMed PMID: 27264869. 10.1016/j.molcel.2016.04.033 27264869

[B243] Rizos H. Darmanian A. P. Mann G. J. Kefford R. F. (2000). Two Arginine Rich Domains in the p14ARF Tumour Suppressor Mediate Nucleolar Localization. Oncogene 19 (26), 2978–2985. PubMed PMID: 10871849. 10.1038/sj.onc.1203629 10871849

[B244] Rocha S. Campbell K. J. Perkins N. D. (2003). p53- and Mdm2-independent Repression of NF-Κb Transactivation by the ARF Tumor Suppressor. Mol. Cell 12 (1), 15–25. PubMed PMID: 12887889. 10.1016/s1097-2765(03)00223-5 12887889

[B245] Rodriguez M. S. Desterro J. M. P. Lain S. Lane D. P. Hay R. T. (2000). Multiple C-Terminal Lysine Residues Target P53 for Ubiquitin-Proteasome-Mediated Degradation. Mol. Cell Biol 20 (22), 8458–8467. PubMed PMID: 11046142; PubMed Central PMCID: PMCPMC102152. 10.1128/MCB.20.22.8458-8467.2000 11046142 PMC102152

[B246] Saadatmandi N. Tyler T. Huang Y. Haghighi A. Frost G. Borgstrom P. (2002). Growth Suppression by a p14ARF Exon 1β Adenovirus in Human Tumor Cell Lines of Varying P53 and Rb Status. Cancer Gene Ther. 9 (10), 830–839. PubMed PMID: 12224024. 10.1038/sj.cgt.7700505 12224024

[B247] Sachdeva M. Zhu S. Wu F. Wu H. Walia V. Kumar S. (2009). p53 Represses C-Myc through Induction of the Tumor Suppressor miR-145. Proc. Natl. Acad. Sci. 106 (9), 3207–3212. PubMed PMID: 19202062; PubMed Central PMCID: PMCPMC2651330. 10.1073/pnas.0808042106 19202062 PMC2651330

[B248] Saito K. Iioka H. Kojima C. Ogawa M. Kondo E. (2016). Peptide‐based Tumor Inhibitor Encoding Mitochondrial P14 ARF Is Highly Efficacious to Diverse Tumors. Cancer Sci. 107 (9), 1290–1301. PubMed PMID: 27317619; PubMed Central PMCID: PMCPMC5021028. 10.1111/cas.12991 27317619 PMC5021028

[B249] Saito K. Takigawa N. Ohtani N. Iioka H. Tomita Y. Ueda R. (2013). Antitumor Impact of p14ARF on Gefitinib-Resistant Non-small Cell Lung Cancers. Mol. Cancer Ther. 12 (8), 1616–1628. PubMed PMID: 23761220. 10.1158/1535-7163.MCT-12-1239 23761220

[B250] Sakaguchi K. Saito S. i. Higashimoto Y. Roy S. Anderson C. W. Appella E. (2000). Damage-mediated Phosphorylation of Human P53 Threonine 18 through a Cascade Mediated by a Casein 1-like Kinase. J. Biol. Chem. 275 (13), 9278–9283. PubMed PMID: 10734067. 10.1074/jbc.275.13.9278 10734067

[B251] Saleh M. N. Patel M. R. Bauer T. M. Goel S. Falchook G. S. Shapiro G. I. (2021). Phase 1 Trial of ALRN-6924, a Dual Inhibitor of MDMX and MDM2, in Patients with Solid Tumors and Lymphomas Bearing Wild-type TP53. Philadelphia: Clin Cancer Res. PubMed PMID: 34301750. 10.1158/1078-0432.CCR-21-0715 PMC9401461 34301750

[B252] Sammons M. A. Nguyen T.-A. T. McDade S. S. Fischer M. (2020). Tumor Suppressor P53: from Engaging DNA to Target Gene Regulation. Nucleic Acids Res. 48 (16), 8848–8869. PubMed PMID: 32797160; PubMed Central PMCID: PMCPMC7498329. 10.1093/nar/gkaa666 32797160 PMC7498329

[B253] Sánchez-Rivera F. J. Ryan J. Soto-Feliciano Y. M. Clare Beytagh M. Xuan L. Feldser D. M. (2021). Mitochondrial Apoptotic Priming Is a Key Determinant of Cell Fate upon P53 Restoration. Proc. Natl. Acad. Sci. U S A. 118, 118. PubMed PMID: 34074758; PubMed Central PMCID: PMCPMC8201929. 10.1073/pnas.2019740118 PMC8201929 34074758

[B254] Sandoval R. Xue J. Pilkinton M. Salvi D. Kiyokawa H. Colamonici O. R. (2004). Different Requirements for the Cytostatic and Apoptotic Effects of Type I Interferons. J. Biol. Chem. 279 (31), 32275–32280. PubMed PMID: 15169789. 10.1074/jbc.M313830200 15169789

[B255] Sapkota D. Kater M. S. J. Sakers K. Nygaard K. R. Liu Y. Lake A. M. (2021). Activity Dependent Translation in Astrocytes Dynamically Alters the Proteome of the Perisynaptic Astrocyte Process. Cold Spring Harbor, NY: bioRxiv, 033027. 10.1101/2020.04.08.033027 PMC9624251 36261025

[B256] Saporita A. J. Chang H.-C. Winkeler C. L. Apicelli A. J. Kladney R. D. Wang J. (2011). RNA Helicase DDX5 Is a P53-independent Target of ARF that Participates in Ribosome Biogenesis. Cancer Res. 71 (21), 6708–6717. PubMed PMID: 21937682; PubMed Central PMCID: PMCPMC3206203. 10.1158/0008-5472.CAN-11-1472 21937682 PMC3206203

[B257] Satpathy S. Krug K. Jean Beltran P. M. Savage S. R. Petralia F. Kumar-Sinha C. (2021). A Proteogenomic Portrait of Lung Squamous Cell Carcinoma. Cell 184 (16), 4348–e40. PubMed PMID: 34358469; PubMed Central PMCID: PMCPMC8475722. 10.1016/j.cell.2021.07.016 34358469 PMC8475722

[B258] Schneider G. Schmidt-Supprian M. Rad R. Saur D. (2017). Tissue-specific Tumorigenesis: Context Matters. Nat. Rev. Cancer 17 (4), 239–253. PubMed PMID: 28256574; PubMed Central PMCID: PMCPMC5823237. 10.1038/nrc.2017.5 28256574 PMC5823237

[B259] Sethi N. S. Kikuchi O. Duronio G. N. Stachler M. D. McFarland J. M. Ferrer-Luna R. (2020). Early TP53 Alterations Engage Environmental Exposures to Promote Gastric Premalignancy in an Integrative Mouse Model. Nat. Genet. 52 (2), 219–230. PubMed PMID: 32025000; PubMed Central PMCID: PMCPMC7031028. 10.1038/s41588-019-0574-9 32025000 PMC7031028

[B260] Sharp D. A. Kratowicz S. A. Sank M. J. George D. L. (1999). Stabilization of the MDM2 Oncoprotein by Interaction with the Structurally Related MDMX Protein. J. Biol. Chem. 274 (53), 38189–38196. PubMed PMID: 10608892. 10.1074/jbc.274.53.38189 10608892

[B261] Sharpless N. E. Ramsey M. R. Balasubramanian P. Castrillon D. H. DePinho R. A. (2004). The Differential Impact of p16INK4a or p19ARF Deficiency on Cell Growth and Tumorigenesis. Oncogene 23 (2), 379–385. PubMed PMID: 14724566. 10.1038/sj.onc.1207074 14724566

[B262] Shen J. P. Zhao D. Sasik R. Luebeck J. Birmingham A. Bojorquez-Gomez A. (2017). Combinatorial CRISPR-Cas9 Screens for De Novo Mapping of Genetic Interactions. Nat. Methods 14 (6), 573–576. 4225. PubMed PMID: 28319113; PubMed Central PMCID: PMCPMC5449203. 10.1038/nmeth10.1038/nmeth.4225 28319113 PMC5449203

[B263] Shieh S.-Y. Ikeda M. Taya Y. Prives C. (1997). DNA Damage-Induced Phosphorylation of P53 Alleviates Inhibition by MDM2. Cell 91 (3), 325–334. PubMed PMID: 9363941. 10.1016/s0092-8674(00)80416-x 9363941

[B264] Shvarts A. Steegenga W. T. Riteco N. van Laar T. Dekker P. Bazuine M. (1996). MDMX: a Novel P53-Binding Protein with Some Functional Properties of MDM2. EMBO J. 15 (19), 5349–5357. PubMed PMID: 8895579; PubMed Central PMCID: PMCPMC452278. 10.1002/j.1460-2075.1996.tb00919.x 8895579 PMC452278

[B265] Siegl C. Rudel T. (2015). Modulation of P53 during Bacterial Infections. Nat. Rev. Microbiol. 13 (12), 741–748. PubMed PMID: 26548915. 10.1038/nrmicro3537 26548915

[B266] Silva J. Domínguez G. Silva J. M. García J. M. Gallego I. Corbacho C. (2001). Analysis of Genetic and Epigenetic Processes that Influence p14ARF Expression in Breast Cancer. Oncogene 20 (33), 4586–4590. PubMed PMID: 11494155. 10.1038/sj.onc.1204617 11494155

[B267] Singh A. K. Chauhan S. S. Singh S. K. Verma V. V. Singh A. Arya R. K. (2016). Dual Targeting of MDM2 with a Novel Small-Molecule Inhibitor Overcomes TRAIL Resistance in Cancer. Carcin 37 (11), 1027–1040. PubMed PMID: 27543608; PubMed Central PMCID: PMCPMC6276916. 10.1093/carcin/bgw088 PMC6276916 27543608

[B268] Singh R. K. Iyappan S. Scheffner M. (2007). Hetero-oligomerization with MdmX Rescues the ubiquitin/Nedd8 Ligase Activity of RING finger Mutants of Mdm2. J. Biol. Chem. 282 (15), 10901–10907. PubMed PMID: 17301054. 10.1074/jbc.M610879200 17301054

[B269] Sinha S. Barbosa K. Cheng K. Leiserson M. D. M. Jain P. Deshpande A. (2021). A Systematic Genome-wide Mapping of Oncogenic Mutation Selection during CRISPR-Cas9 Genome Editing. Nat. Commun. 12 (1), 6512. PubMed PMID: 34764240; PubMed Central PMCID: PMCPMC8586238. 10.1038/s41467-021-26788-6 34764240 PMC8586238

[B270] Song X. Sturgis E. M. Huang Z. Li X. Li C. Wei Q. (2014). Potentially Functional Variants ofp14ARFare Associated with HPV-Positive Oropharyngeal Cancer Patients and Survival after Definitive Chemoradiotherapy. Carcin 35 (1), 62–68. PubMed PMID: 24104554; PubMed Central PMCID: PMCPMC3871940. 10.1093/carcin/bgt336 PMC3871940 24104554

[B271] Soragni A. Janzen D. M. Johnson L. M. Lindgren A. G. Thai-Quynh Nguyen A. Tiourin E. (2016). A Designed Inhibitor of P53 Aggregation Rescues P53 Tumor Suppression in Ovarian Carcinomas. Cancer Cell 29 (1), 90–103. PubMed PMID: 26748848; PubMed Central PMCID: PMCPMC4733364. 10.1016/j.ccell.2015.12.002 26748848 PMC4733364

[B272] Sorolla A. Wang E. Golden E. Duffy C. Henriques S. T. Redfern A. D. (2020). Precision Medicine by Designer Interference Peptides: Applications in Oncology and Molecular Therapeutics. Oncogene 39 (6), 1167–1184. PubMed PMID: 31636382; PubMed Central PMCID: PMCPMC7002299. 10.1038/s41388-019-1056-3 31636382 PMC7002299

[B273] Stambolic V. MacPherson D. Sas D. Lin Y. Snow B. Jang Y. (2001). Regulation of PTEN Transcription by P53. Mol. Cell 8 (2), 317–325. PubMed PMID: 11545734. 10.1016/s1097-2765(01)00323-9 11545734

[B274] Stewart-Ornstein J. Cheng H. W. Lahav G. (2017). Conservation and Divergence of P53 Oscillation Dynamics across Species. Cell Syst. 5 (4), 410–417. PubMed PMID: 29055670; PubMed Central PMCID: PMCPMC5687840. 10.1016/j.cels.2017.09.012 29055670 PMC5687840

[B275] Stindt M. H. Carter S. A. Vigneron A. M. Ryan K. M. Vousden K. H. (2011). MDM2 Promotes SUMO-2/3 Modification of P53 to Modulate Transcriptional Activity. Cell Cycle 10 (18), 3176–3188. PubMed PMID: 21900752; PubMed Central PMCID: PMCPMC3218624. 10.4161/cc.10.18.17436 21900752 PMC3218624

[B276] Stockwell B. R. Friedmann Angeli J. P. Bayir H. Bush A. I. Conrad M. Dixon S. J. (2017). Ferroptosis: A Regulated Cell Death Nexus Linking Metabolism, Redox Biology, and Disease. Cell 171 (2), 273–285. PubMed PMID: 28985560; PubMed Central PMCID: PMCPMC5685180. 10.1016/j.cell.2017.09.021 28985560 PMC5685180

[B277] Sugimoto M. Kuo M.-L. Roussel M. F. Sherr C. J. (2003). Nucleolar Arf Tumor Suppressor Inhibits Ribosomal RNA Processing. Mol. Cell 11 (2), 415–424. PubMed PMID: 12620229. 10.1016/s1097-2765(03)00057-1 12620229

[B278] Szybinska A. Lesniak W. (2017). P53 Dysfunction in Neurodegenerative Diseases - the Cause or Effect of Pathological Changes? Aging Dis. 8 (4), 506–518. PubMed PMID: 28840063; PubMed Central PMCID: PMCPMC5524811. 10.14336/AD.2016.1120 28840063 PMC5524811

[B279] Tago K. Chiocca S. Sherr C. J. (2005). Sumoylation Induced by the Arf Tumor Suppressor: a P53-independent Function. Proc. Natl. Acad. Sci. 102 (21), 7689–7694. PubMed PMID: 15897463; PubMed Central PMCID: PMCPMC1129025. 10.1073/pnas.0502978102 15897463 PMC1129025

[B280] Tago K. Funakoshi-Tago M. Itoh H. Furukawa Y. Kikuchi J. Kato T. (2015). Arf Tumor Suppressor Disrupts the Oncogenic Positive Feedback Loop Including C-Myc and DDX5. Oncogene 34 (3), 314–322. PubMed PMID: 24469041. 10.1038/onc.2013.561 24469041

[B281] Takatori H. Kawashima H. Suzuki K. Nakajima H. (2014). Role of P53 in Systemic Autoimmune Diseases. Crit. Rev. Immunol. 34 (6), 509–516. PubMed PMID: 25597313. 10.1615/critrevimmunol.2014012193 25597313

[B282] Tan B. X. Liew H. P. Chua J. S. Ghadessy F. J. Tan Y. S. Lane D. P. (2017). Anatomy of Mdm2 and Mdm4 in Evolution. J. Mol. Cell Biol 9 (1), 3–15. PubMed PMID: 28077607; PubMed Central PMCID: PMCPMC6372010. 10.1093/jmcb/mjx002 28077607 PMC6372010

[B283] Tang D. Kang R. Berghe T. V. Vandenabeele P. Kroemer G. (2019). The Molecular Machinery of Regulated Cell Death. Cell Res 29 (5), 347–364. PubMed PMID: 30948788; PubMed Central PMCID: PMCPMC6796845. 10.1038/s41422-019-0164-5 30948788 PMC6796845

[B284] Tang Y. Zhao W. Chen Y. Zhao Y. Gu W. (2008). Acetylation Is Indispensable for P53 Activation. Cell 133 (4), 612–626. PubMed PMID: 18485870; PubMed Central PMCID: PMCPMC2914560. 10.1016/j.cell.2008.03.025 18485870 PMC2914560

[B285] Tango Y. Fujiwara T. Itoshima T. Takata Y. Katsuda K. Uno F. (2002). Adenovirus-Mediated p14ARFGene Transfer Cooperates with Ad5CMV-P53 to Induce Apoptosis in Human Cancer Cells. Hum. Gene Ther. 13 (11), 1373–1382. PubMed PMID: 12162819. 10.1089/104303402760128595 12162819

[B286] Tao W. Levine A. J. (1999). P19ARF Stabilizes P53 by Blocking Nucleo-Cytoplasmic Shuttling of Mdm2. Proc. Natl. Acad. Sci. 96 (12), 6937–6941. PubMed PMID: 10359817; PubMed Central PMCID: PMCPMC22020. 10.1073/pnas.96.12.6937 10359817 PMC22020

[B287] Tarangelo A. Magtanong L. Bieging-Rolett K. T. Li Y. Ye J. Attardi L. D. (2018). p53 Suppresses Metabolic Stress-Induced Ferroptosis in Cancer Cells. Cell Rep. 22 (3), 569–575. PubMed PMID: 29346757; PubMed Central PMCID: PMCPMC5791910. 10.1016/j.celrep.2017.12.077 29346757 PMC5791910

[B288] Terzian T. Suh Y.-A. Iwakuma T. Post S. M. Neumann M. Lang G. A. (2008). The Inherent Instability of Mutant P53 Is Alleviated by Mdm2 or p16INK4a Loss. Genes Dev. 22 (10), 1337–1344. PubMed PMID: 18483220; PubMed Central PMCID: PMCPMC2377188. 10.1101/gad.1662908 18483220 PMC2377188

[B289] Tollini L. A. Jin A. Park J. Zhang Y. (2014). Regulation of P53 by Mdm2 E3 Ligase Function Is Dispensable in Embryogenesis and Development, but Essential in Response to DNA Damage. Cancer Cell 26 (2), 235–247. PubMed PMID: 25117711; PubMed Central PMCID: PMCPMC4369778. 10.1016/j.ccr.2014.06.006 25117711 PMC4369778

[B290] Uldrijan S. Pannekoek W.-J. Vousden K. H. (2007). An Essential Function of the Extreme C-Terminus of MDM2 Can Be provided by MDMX. EMBO J. 26 (1), 102–112. PubMed PMID: 17159902; PubMed Central PMCID: PMCPMC1782374. 10.1038/sj.emboj.7601469 17159902 PMC1782374

[B291] Unger T. Juven-Gershon T. Moallem E. Berger M. Vogt Sionov R. Lozano G. (1999). Critical Role for Ser20 of Human P53 in the Negative Regulation of P53 by Mdm2. EMBO J. 18 (7), 1805–1814. PubMed PMID: 10202144; PubMed Central PMCID: PMCPMC1171266. 10.1093/emboj/18.7.1805 10202144 PMC1171266

[B292] van Oosterwijk J. G. Li C. Yang X. Opferman J. T. Sherr C. J. (2017). Small Mitochondrial Arf (smArf) Protein Corrects P53-independent Developmental Defects of Arf Tumor Suppressor-Deficient Mice. Proc. Natl. Acad. Sci. USA 114 (28), 7420–7425. PubMed PMID: 28652370; PubMed Central PMCID: PMCPMC5514764. 10.1073/pnas.1707292114 28652370 PMC5514764

[B293] Vaseva A. V. Yallowitz A. R. Marchenko N. D. Xu S. Moll U. M. (2011). Blockade of Hsp90 by 17AAG Antagonizes MDMX and Synergizes with Nutlin to Induce P53-Mediated Apoptosis in Solid Tumors. Cell Death Dis 2, e156. e156PubMed PMID: 21562588; PubMed Central PMCID: PMCPMC3122118. 10.1038/cddis.2011.39 21562588 PMC3122118

[B294] Venkatesh D. O'Brien N. A. Zandkarimi F. Tong D. R. Stokes M. E. Dunn D. E. (2020). MDM2 and MDMX Promote Ferroptosis by PPARα-Mediated Lipid Remodeling. Genes Dev. 34 (7-8), 526–543. PubMed PMID: 32079652; PubMed Central PMCID: PMCPMC7111265. 10.1101/gad.334219.119 32079652 PMC7111265

[B295] Vivo M. Fontana R. Ranieri M. Capasso G. Angrisano T. Pollice A. (2017). p14ARF Interacts with the Focal Adhesion Kinase and Protects Cells from Anoikis. Oncogene 36 (34), 4913–4928. PubMed PMID: 28436949; PubMed Central PMCID: PMCPMC5582215. 10.1038/onc.2017.104 28436949 PMC5582215

[B296] Vogelstein B. Sur S. Prives C. (2010). p53: The Most Frequently Altered Gene in Human Cancers. Nat. Edu. 3, 6.

[B297] Waldman A. D. Fritz J. M. Lenardo M. J. (2020). A Guide to Cancer Immunotherapy: from T Cell Basic Science to Clinical Practice. Nat. Rev. Immunol. 20 (11), 651–668. PubMed PMID: 32433532; PubMed Central PMCID: PMCPMC7238960. 10.1038/s41577-020-0306-5 32433532 PMC7238960

[B298] Wang H. Ma X. Ren S. Buolamwini J. K. Yan C. (2011). A Small-Molecule Inhibitor of MDMX Activates P53 and Induces Apoptosis. Mol. Cancer Ther. 10 (1), 69–79. PubMed PMID: 21075910; PubMed Central PMCID: PMCPMC3058295. 10.1158/1535-7163.MCT-10-0581 21075910 PMC3058295

[B299] Wang H. Yan C. (2011). A Small-Molecule P53 Activator Induces Apoptosis through Inhibiting MDMX Expression in Breast Cancer Cells. Neoplasia 13 (7), 611–IN6. PubMed PMID: 21750655; PubMed Central PMCID: PMCPMC3132847. 10.1593/neo.11438 21750655 PMC3132847

[B300] Wang J. Chen Y. Huang C. Hao Q. Zeng S. X. Omari S. (2021). Valosin-Containing Protein Stabilizes Mutant P53 to Promote Pancreatic Cancer Growth. Cancer Res. 81 (15), 4041–4053. PubMed PMID: 34099490. 10.1158/0008-5472.CAN-20-3855 34099490

[B301] Wang L. Liu R. Ye P. Wong C. Chen G.-Y. Zhou P. (2015). Intracellular CD24 Disrupts the ARF-NPM Interaction and Enables Mutational and Viral Oncogene-Mediated P53 Inactivation. Nat. Commun. 6, 5909. PubMed PMID: 25600590; PubMed Central PMCID: PMCPMC4300525. 10.1038/ncomms6909 25600590 PMC4300525

[B302] Wang S.-P. Wang W.-L. Chang Y.-L. Wu C.-T. Chao Y.-C. Kao S.-H. (2009). p53 Controls Cancer Cell Invasion by Inducing the MDM2-Mediated Degradation of Slug. Nat. Cell Biol 11 (6), 694–704. PubMed PMID: 19448627. 10.1038/ncb1875 19448627

[B303] Wang W. Cheng J.-W. Qin J.-J. Hu B. Li X. Nijampatnam B. (2019). MDM2-NFAT1 Dual Inhibitor, MA242: Effective against Hepatocellular Carcinoma, Independent of P53. Cancer Lett. 459, 156–167. PubMed PMID: 31181320; PubMed Central PMCID: PMCPMC6650270. 10.1016/j.canlet.2019.114429 31181320 PMC6650270

[B304] Wang W. Qin J.-J. Voruganti S. Wang M.-H. Sharma H. Patil S. (2014). Identification of a New Class of MDM2 Inhibitor that Inhibits Growth of Orthotopic Pancreatic Tumors in Mice. Gastroenterology 147 (4), 893–902. PubMed PMID: 25016295; PubMed Central PMCID: PMCPMC4170027. 10.1053/j.gastro.2014.07.001 25016295 PMC4170027

[B305] Weber J. D. Jeffers J. R. Rehg J. E. Randle D. H. Lozano G. Roussel M. F. (2000). p53-independent Functions of the p19ARF Tumor Suppressor. Genes Dev. 14 (18), 2358–2365. PubMed PMID: 10995391; PubMed Central PMCID: PMCPMC316930. 10.1101/gad.827300 10995391 PMC316930

[B306] Weber J. D. Kuo M.-L. Bothner B. DiGiammarino E. L. Kriwacki R. W. Roussel M. F. (2000). Cooperative Signals Governing ARF-Mdm2 Interaction and Nucleolar Localization of the Complex. Mol. Cell Biol 20 (7), 2517–2528. PubMed PMID: 10713175; PubMed Central PMCID: PMCPMC85460. 10.1128/MCB.20.7.2517-2528.2000 10713175 PMC85460

[B307] Weber J. D. Taylor L. J. Roussel M. F. Sherr C. J. Bar-Sagi D. (1999). Nucleolar Arf Sequesters Mdm2 and Activates P53. Nat. Cell Biol 1 (1), 20–26. PubMed PMID: 10559859. 10.1038/8991 10559859

[B308] Wellenstein M. D. de Visser K. E. (2018). Cancer-Cell-Intrinsic Mechanisms Shaping the Tumor Immune Landscape. Immunity 48 (3), 399–416. PubMed PMID: 29562192. 10.1016/j.immuni.2018.03.004 29562192

[B309] Witkiewicz A. K. Chung S. Brough R. Vail P. Franco J. Lord C. J. (2018). Targeting the Vulnerability of RB Tumor Suppressor Loss in Triple-Negative Breast Cancer. Cell Rep. 22 (5), 1185–1199. PubMed PMID: 29386107; PubMed Central PMCID: PMCPMC5967622. 10.1016/j.celrep.2018.01.022 29386107 PMC5967622

[B310] Wu D. Prives C. (2018). Relevance of the P53-MDM2 axis to Aging. Cell Death Differ 25 (1), 169–179. PubMed PMID: 29192902; PubMed Central PMCID: PMCPMC5729541. 10.1038/cdd.2017.187 29192902 PMC5729541

[B311] Wu W. Xu C. Ling X. Fan C. Buckley B. P. Chernov M. V. (2015). Targeting RING Domains of Mdm2-MdmX E3 Complex Activates Apoptotic Arm of the P53 Pathway in Leukemia/lymphoma Cells. Cell Death Dis 6, e2035. e2035PubMed PMID: 26720344; PubMed Central PMCID: PMCPMC4720891. 10.1038/cddis.2015.358 26720344 PMC4720891

[B312] Wu Z. Zhou J. Zhang X. Zhang Z. Xie Y. Liu J. b. (2021). Reprogramming of the Esophageal Squamous Carcinoma Epigenome by SOX2 Promotes ADAR1 Dependence. Nat. Genet. 53 (6), 881–894. PubMed PMID: 33972779. 10.1038/s41588-021-00859-2 33972779 PMC9124436

[B313] Xia Y. Li X. Sun W. (2020). Applications of Recombinant Adenovirus-P53 Gene Therapy for Cancers in the Clinic in China. Cgt 20 (2), 127–141. PubMed PMID: 32951572. 10.2174/1566523220999200731003206 32951572

[B314] Xiao Y. Thakkar K. N. Zhao H. Broughton J. Li Y. Seoane J. A. (2020). The m6A RNA Demethylase FTO Is a HIF-independent Synthetic Lethal Partner with the VHL Tumor Suppressor. Proc. Natl. Acad. Sci. USA 117 (35), 21441–21449. PubMed PMID: 32817424; PubMed Central PMCID: PMCPMC7474618. 10.1073/pnas.2000516117 32817424 PMC7474618

[B315] Xiao Z. Dai Z. Locasale J. W. (2019). Metabolic Landscape of the Tumor Microenvironment at Single Cell Resolution. Nat. Commun. 10 (1), 3763. PubMed PMID: 31434891; PubMed Central PMCID: PMCPMC6704063. 10.1038/s41467-019-11738-0 31434891 PMC6704063

[B316] Xie M. Liu D. Yang Y. (2020). Anti-cancer Peptides: Classification, Mechanism of Action, Reconstruction and Modification. Open Biol. 10 (7), 200004. PubMed PMID: 32692959; PubMed Central PMCID: PMCPMC7574553. 10.1098/rsob.200004 32692959 PMC7574553

[B317] Xirodimas D. P. Chisholm J. Desterro J. M. Lane D. P. Hay R. T. (2002). P14ARF Promotes Accumulation of SUMO-1 Conjugated (H)Mdm2. FEBS Lett. 528 (1-3), 207–211. PubMed PMID: 12297306. 10.1016/s0014-5793(02)03310-0 12297306

[B318] Xirodimas D. P. Saville M. K. Bourdon J.-C. Hay R. T. Lane D. P. (2004). Mdm2-mediated NEDD8 Conjugation of P53 Inhibits its Transcriptional Activity. Cell 118 (1), 83–97. PubMed PMID: 15242646. 10.1016/j.cell.2004.06.016 15242646

[B319] Xu C. L. Sang B. Liu G. Z. Li J. M. Zhang X. D. Liu L. X. (2020). SENEBLOC, a Long Non-coding RNA Suppresses Senescence via P53-dependent and Independent Mechanisms. Nucleic Acids Res. 48 (6), 3089–3102. PubMed PMID: 32030426; PubMed Central PMCID: PMCPMC7102969. 10.1093/nar/gkaa063 32030426 PMC7102969

[B320] Xu J. Han M. Shen J. Guan Q. Bai Z. Lang B. (2016). 2-Methoxy-5((3,4,5-trimethosyphenyl)seleninyl) Phenol Inhibits MDM2 and Induces Apoptosis in Breast Cancer Cells through a P53-independent Pathway. Cancer Lett. 383 (1), 9–17. PubMed PMID: 27693458. 10.1016/j.canlet.2016.09.011 27693458

[B321] Xu X.-q. Pan X.-h. Wang T.-t. Wang J. Yang B. He Q.-j. (2021). Intrinsic and Acquired Resistance to CDK4/6 Inhibitors and Potential Overcoming Strategies. Acta Pharmacol. Sin 42 (2), 171–178. PubMed PMID: 32504067; PubMed Central PMCID: PMCPMC8027849. 10.1038/s41401-020-0416-4 32504067 PMC8027849

[B322] Yamada L. Saito M. Thar Min A. K. Saito K. Ashizawa M. Kase K. (2021). Selective Sensitivity of EZH2 Inhibitors Based on Synthetic Lethality in ARID1A-Deficient Gastric Cancer. Gastric Cancer 24 (1), 60–71. PubMed PMID: 32506298. 10.1007/s10120-020-01094-0 32506298

[B323] Yan C. Lu D. Hai T. Boyd D. D. (2005). Activating Transcription Factor 3, a Stress Sensor, Activates P53 by Blocking its Ubiquitination. EMBO J. 24 (13), 2425–2435. PubMed PMID: 15933712; PubMed Central PMCID: PMCPMC1173153. 10.1038/sj.emboj.7600712 15933712 PMC1173153

[B324] Yang L. Song T. Cheng Q. Chen L. Chen J. (2019). Mutant P53 Sequestration of the MDM2 Acidic Domain Inhibits E3 Ligase Activity. Mol. Cell Biol 39, 39. PubMed PMID: 30455251; PubMed Central PMCID: PMCPMC6362316. 10.1128/MCB.00375-18 PMC6362316 30455251

[B325] Yao C. H. Wang R. Wang Y. Kung C. P. Weber J. D. Patti G. J. (2019). Mitochondrial Fusion Supports Increased Oxidative Phosphorylation during Cell Proliferation. Elife 8, 8. PubMed PMID: 30694178; PubMed Central PMCID: PMCPMC6351101. 10.7554/eLife.41351 PMC6351101 30694178

[B326] Ye P. Mimura J. Okada T. Sato H. Liu T. Maruyama A. (2014). Nrf2- and ATF4-dependent Upregulation of xCT Modulates the Sensitivity of T24 Bladder Carcinoma Cells to Proteasome Inhibition. Mol. Cell Biol 34 (18), 3421–3434. PubMed PMID: 25002527; PubMed Central PMCID: PMCPMC4135628. 10.1128/MCB.00221-14 25002527 PMC4135628

[B327] Yetil A. Anchang B. Gouw A. M. Adam S. J. Zabuawala T. Parameswaran R. (2015). p19ARF Is a Critical Mediator of Both Cellular Senescence and an Innate Immune Response Associated with MYC Inactivation in Mouse Model of Acute Leukemia. Oncotarget 6 (6), 3563–3577. 2969. PubMed PMID: 25784651; PubMed Central PMCID: PMCPMC4414137. 10.18632/oncotarget10.18632/oncotarget.2969 25784651 PMC4414137

[B328] Yu G. W. Rudiger S. Veprintsev D. Freund S. Fernandez-Fernandez M. R. Fersht A. R. (2006). The central Region of HDM2 Provides a Second Binding Site for P53. Proc. Natl. Acad. Sci. 103 (5), 1227–1232. PubMed PMID: 16432196; PubMed Central PMCID: PMCPMC1360574. 10.1073/pnas.0510343103 16432196 PMC1360574

[B329] Yue X. Zhao Y. Liu J. Zhang C. Yu H. Wang J. (2015). BAG2 Promotes Tumorigenesis through Enhancing Mutant P53 Protein Levels and Function. Elife 4, 4. PubMed PMID: 26271008; PubMed Central PMCID: PMCPMC4561369. 10.7554/eLife.08401 PMC4561369 26271008

[B330] Zanjirband M. Rahgozar S. (2019). Targeting P53-MDM2 Interaction Using Small Molecule Inhibitors and the Challenges Needed to Be Addressed. Cdt 20 (11), 1091–1111. PubMed PMID: 30947669. 10.2174/1389450120666190402120701 30947669

[B331] Zeng Y. Kotake Y. Pei X.-H. Smith M. D. Xiong Y. (2011). p53 Binds to and Is Required for the Repression ofArfTumor Suppressor by HDAC and Polycomb. Cancer Res. 71 (7), 2781–2792. PubMed PMID: 21447739; PubMed Central PMCID: PMCPMC3227421. 10.1158/0008-5472.CAN-10-3483 21447739 PMC3227421

[B332] Zhang H. Gu L. Liu T. Chiang K.-Y. Zhou M. (2014). Inhibition of MDM2 by Nilotinib Contributes to Cytotoxicity in Both Philadelphia-positive and Negative Acute Lymphoblastic Leukemia. PLoS One 9 (6), e100960. PubMed PMID: 24968304; PubMed Central PMCID: PMCPMC4072773. 10.1371/journal.pone.0100960 24968304 PMC4072773

[B333] Zhang Q. Zeng S. X. Zhang Y. Zhang Y. Ding D. Ye Q. (2012). A Small Molecule Inauhzin Inhibits SIRT1 Activity and Suppresses Tumour Growth through Activation of P53. EMBO Mol. Med. 4 (4), 298–312. PubMed PMID: 22331558; PubMed Central PMCID: PMCPMC3376857. 10.1002/emmm.201100211 22331558 PMC3376857

[B334] Zhang Y. Xiong Y. (1999). Mutations in Human ARF Exon 2 Disrupt its Nucleolar Localization and Impair its Ability to Block Nuclear export of MDM2 and P53. Mol. Cell 3 (5), 579–591. PubMed PMID: 10360174. 10.1016/s1097-2765(00)80351-2 10360174

[B335] Zhao X. Li J. Liu Z. Powers S. (2021). Combinatorial CRISPR/Cas9 Screening Reveals Epistatic Networks of Interacting Tumor Suppressor Genes and Therapeutic Targets in Human Breast Cancer. Cancer Res. 81 (24), 6090–6105. PubMed PMID: 34561273. 10.1158/0008-5472.CAN-21-2555 34561273 PMC9762330

[B336] Zhou X. Singh M. Sanz Santos G. Guerlavais V. Carvajal L. A. Aivado M. (2021). Pharmacological Activation of P53 Triggers Viral Mimicry Response Thereby Abolishing Tumor Immune Evasion and Promoting Anti-tumor Immunity. Philadelphia: Cancer Discov. PubMed PMID: 34230007. 10.1158/2159-8290.CD-20-1741 PMC9414294 34230007

[B337] Zhou X. Smith Q. R. Liu X. (2021). Brain Penetrating Peptides and Peptide-Drug Conjugates to Overcome the Blood-Brain Barrier and Target CNS Diseases. Wiley Interdiscip. Rev. Nanomed Nanobiotechnol 13 (4), e1695. 1695. PubMed PMID: 33470550. 10.1002/wnan10.1002/wnan.1695 33470550

[B338] Zhu D. Xu S. Deyanat-Yazdi G. Peng S. X. Barnes L. A. Narla R. K. (2018). Synthetic Lethal Strategy Identifies a Potent and Selective TTK and CLK1/2 Inhibitor for Treatment of Triple-Negative Breast Cancer with a Compromised G1-S Checkpoint. Mol. Cancer Ther. 17 (8), 1727–1738. PubMed PMID: 29866747. 10.1158/1535-7163.MCT-17-1084 29866747

[B339] Zhu Z. Song H. Xu J. (2021). CDKN2A Deletion in Melanoma Excludes T Cell Infiltration by Repressing Chemokine Expression in a Cell Cycle-dependent Manner. Front. Oncol. 11, 641077. PubMed PMID: 33842347; PubMed Central PMCID: PMCPMC8027313. 10.3389/fonc.2021.641077 33842347 PMC8027313

[B340] Zhuang C. Miao Z. Zhu L. Dong G. Guo Z. Wang S. (2012). Discovery, Synthesis, and Biological Evaluation of Orally Active Pyrrolidone Derivatives as Novel Inhibitors of P53-MDM2 Protein-Protein Interaction. J. Med. Chem. 55 (22), 9630–9642. PubMed PMID: 23046248. 10.1021/jm300969t 23046248

[B341] Zindy F. Eischen C. M. Randle D. H. Kamijo T. Cleveland J. L. Sherr C. J. (1998). Myc Signaling via the ARF Tumor Suppressor Regulates P53-dependent Apoptosis and Immortalization. Genes Dev. 12 (15), 2424–2433. PubMed PMID: 9694806; PubMed Central PMCID: PMCPMC317045. 10.1101/gad.12.15.2424 9694806 PMC317045

